# Redox-modulating macrophage biohybrid nanoplatform for targeted RIPK1-PANoptosome suppression in ischemic stroke

**DOI:** 10.1016/j.redox.2025.103997

**Published:** 2025-12-24

**Authors:** Wenhui Jiang, Chundongqiu Xia, Zhimeng Cui, Lanhao Shi, Wei Feng, Yu Chen, Jun Zhang

**Affiliations:** a Department of Radiology, Huadong Hospital, Shanghai Key Laboratory of Clinical Geriatric Medicine, Shanghai Institute of Geriatrics and Gerontology, State Key Laboratory of Brain Function and Disorders, Fudan University, Shanghai, 200040, PR China; b Materdicine Lab, School of Life Sciences, Shanghai University, Shanghai, 200444, PR China

**Keywords:** Ischemic stroke, Live cell therapy, PANoptosis regulation, Cell tracking, Anti-inflammation

## Abstract

Disruption of redox homeostasis during reperfusion triggers a complex and dynamic neuroinflammatory cascade in ischemic stroke, posing a major barrier to precision therapy. Although cellular therapies have emerged as a promising strategy, their clinical translation is hindered by the lack of tools capable of simultaneously tracking cell delivery and modulating pathological redox imbalance in vivo. Here, we report an MRI-trackable engineered macrophage-derived biohybrid nanoplatform that integrates the inflammation-homing capacity of macrophages, the imaging functionality of ultrasmall superparamagnetic iron oxide nanoparticles, and the redox-regulating activity of 2,2,6,6-tetramethylpiperidine-1-oxyl-doped lipids to restore local redox homeostasis. Leveraging their innate tropism toward inflamed tissue, the engineered macrophages selectively accumulate at sites of post-ischemic neuroinflammation. Concurrently, their intrinsic reactive oxygen species-scavenging capability alleviates oxidative stress, thereby suppressing a redox-dependent network of programmed cell death PANoptosis. In vivo ischemic stroke models demonstrate that this redox-modulating biohybrid nanoplatform significantly inhibits oxidative stress-induced PANoptosis, leading to enhanced neuronal survival and improved neurological recovery. Notably, the engineered macrophages function as both redox state imagers and active modulators, enabling real-time visualization and spatiotemporal regulation of redox dynamics within the ischemic brain. Collectively, this work establishes a precision theranostic strategy to disrupt the oxidative stress-PANoptosis axis and highlights a broadly applicable platform for the treatment of oxidative stress and inflammation associated diseases.

## Highlights

• Engineered macrophage-derived MRI trackable biohybrid nanoplatform (MA@ULips) retained the homing ability toward inflammatory sites, precisely navigated to neuroinflammatory regions, enabling precisely targeted therapy for neuroinflammation.
• MA@ULips suppressed the PANoptosis pathway by inhibiting multiple cell death mechanisms, overcoming the limitations of traditional anti-inflammatory monotherapeutic strategies.
• MA@ULips enabled integrated theranostics for neuroinflammation in acute ischemic stroke by MRI-guided monitoring of macrophage distribution.

## Graphical abstract

**Figure:**
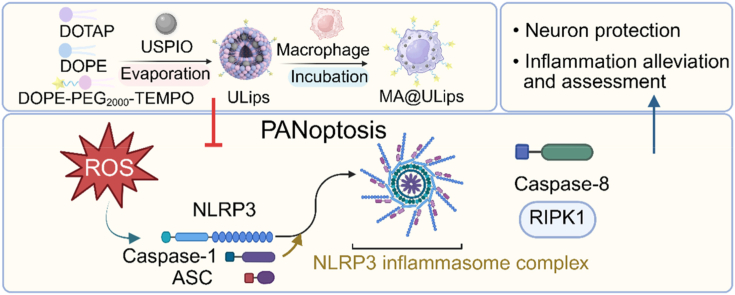

## 1 Introduction

Ischemic stroke remains one of the leading causes of disability worldwide, imposing significant public health burdens due to its high incidence and long-term effects on neurological function [[1], [2], [3]]. Although current therapeutic strategies such as thrombolysis and mechanical thrombectomy are effective at restoring blood flow by removing clots, less than 5 % of acute ischemic stroke patients receive timely intervention within the narrow therapeutic window required for these approaches [4,5]. Moreover, even in patients who undergo successful intervention, many continue to suffer from long-term neurological deficits [6]. This is primarily due to post-stroke neuroinflammation, which is closely associated with poor recovery outcomes [7]. The inflammatory cascade is driven by reactive oxygen species (ROS), activation of microglia and astrocytes, and the subsequent secretion of pro-inflammatory cytokines [8,9]. Although traditional anti-inflammatory drugs have been incorporated into treatment regimens, their clinical efficacy is often limited by poor blood-brain barrier (BBB) penetration, off-target effects, and suboptimal therapeutic outcomes [10].

Nanoparticle-based drug delivery systems have shown promise in disease treatment, but their utility is often hindered by short blood circulation lifetime and insufficient drug accumulation at target sites [11]. Recently, drug delivery systems based on living cells have emerged as promising alternatives for the targeted delivery of therapeutics, offering several advantages, including enhanced precision targeting, the ability to cross biological barriers such as BBB, and the potential for personalized treatment [[12], [13], [14]]. There are multiple approaches to exploit the delivery capability of natural cells, such as isolating the cell membrane for nanoparticle coating and using a diverse toolbox of bioengineering methodologies to achieve live cell engineering [15]. However, the cell membrane has only limited functionalities, and full surface covering of biomimetic nanoparticles is rare, which may reduce the intended delivery effect [16]. Membrane-coated systems merely serve as passive carriers, lacking metabolic activity and efficient active recruitment. In comparison, by engineering living cells, specific targeting capabilities and biological functionalities can be imparted, providing innovative solutions for the diagnosis and treatment of diseases [[17], [18], [19], [20]]. Among various cell types under investigation, immune cells have garnered significant attention due to their intrinsic ability to cross BBB, localize to inflamed tissue, and evade immune clearance [21,22]. Macrophages, in particular, are key players in the inflammatory response and tissue repair processes [23]. In response to inflammatory signals, they are recruited to sites of injury, where they mediate tissue repair through chemotaxis. By harnessing the natural targeting capability, engineered macrophages can serve as effective vehicles, enabling the precise delivery of therapeutic agents to ischemic brain tissue, thereby improving the efficacy of stroke treatments [24].

In the past decade, researchers have explored the targeting of various cell death mechanisms to enhance antioxidant and anti-inflammatory responses in the brain [[25], [26], [27], [28]]. However, traditional neuroprotective strategies that focus on single cell death pathways such as pyroptosis, apoptosis, or specific anti-inflammation mechanisms may be insufficient due to the complex, multifactorial nature of post-stroke neuroinflammation [[29], [30], [31]]. A promising approach for ischemic stroke therapy involves targeting PANoptosis, a recently defined form of programmed cell death that integrates pyroptosis, apoptosis, and necroptosis under the regulatory control of the PANoptosome complex [[32], [33], [34], [35], [36]]. PANoptosis offers a convergent point for multiple programmed cell death pathways and represents a novel strategy for managing inflammation and cell death in ischemic stroke [37,38]. By targeting the integrated PANoptosis pathway, it would be possible to mitigate neuroinflammation and improve outcomes following ischemic brain injury [38].

**Scheme 1:**
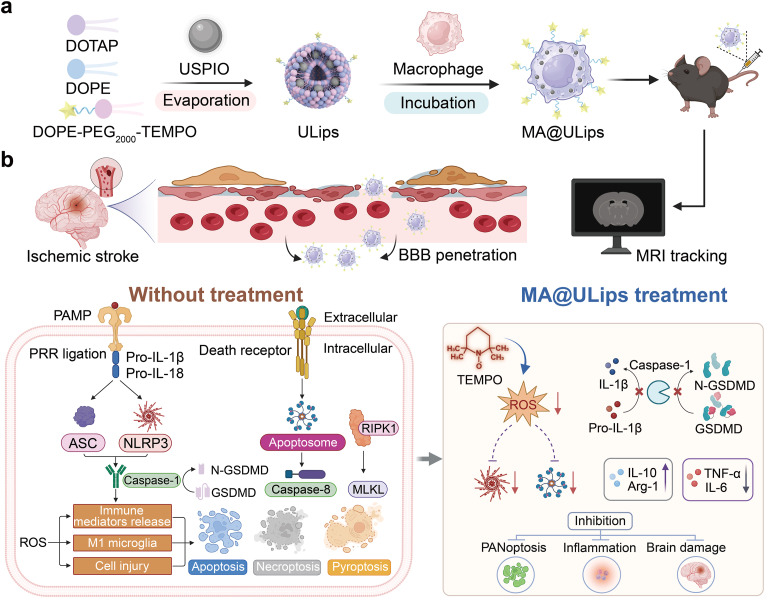
Schematic illustration of MA@ULips for ischemic stroke therapy and anti-inflammation assessment. (a) Preparation of MA@ULips: MA@ULips are synthesized through the membrane fusion of macrophages and ULips. (b) Neuroprotective effects and therapeutic monitoring: In a murine model of ischemia, MA@ULips cross the BBB. The incorporation of USPIO into the liposomes allows for real-time MRI tracking of macrophage migration. MA@ULips exert neuroprotective effects through direct scavenging of ROS and inhibition of PANoptosis.

Magnetic resonance imaging (MRI) remains a standard tool for the diagnosis of ischemic stroke [39,40]. Due to its non-invasive nature, high spatial resolution, deep tissue penetration, and relatively long retention times of contrast agents, MRI has become a valuable technique for cell tracking in preclinical and clinical studies [18,41,42]. However, the effects of contrast agent labeling on macrophages and their subsequent biological responses post-injection remain poorly understood [43]. Herein, we introduce an inflammation-activated engineered macrophage-derived MRI trackable biohybrid nanoplatform (MA@ULips) designed to regulate multi-pathway cell death inhibition, providing a precise therapeutic strategy for ischemic stroke. The MA@ULips is constructed by coincubating macrophages (MA) with membrane structured, magnetic nanoparticle-loaded liposomes (ULips). The system integrates the benefits of ultrasmall superparamagnetic iron oxide (USPIO) nanoparticles, which facilitate cell tracking via MRI, and 2,2,6,6-tetramethylpiperidine-1-oxyl (TEMPO), which serves both as a ROS scavenger and an inhibitor of receptor-interacting protein kinase 1 (RIPK1)-mediated PANoptosome complex formation. MA@ULips not only enhance accumulation at the ischemic site but also significantly reduces ROS levels, inhibits the PANoptotic cell death pathway, and promotes the polarization of microglia towards an anti-inflammatory phenotype (Scheme 1). These combined effects contribute to the regulation of neuroinflammation in the context of ischemia-reperfusion injury. By integrating multiple mechanisms of action, MA@ULips offer a multi-target approach to modulate cell death pathways after ischemic stroke. Moreover, this platform holds promise for advancing neuroprotective strategies applicable to a broad range of neurodegenerative conditions, thereby providing valuable insights into the development of therapeutic interventions for brain diseases.

## 2 Experimental section

### 2.1 Materials and reagents

N-hydroxysulfosuccinimide sodium salt (Sulfo-NHS) and N-(3-dimethylaminopropyl)-N′-ethyl carbodiimide hydrochloride (EDC) were purchased from Macklin Biochemical Co. Ltd. (Shanghai, China). 4-Amino-2, 2, 6, 6- tetramethylpiperidine 1-oxyl (TEMPO-NH_2_), 1, 2-dioleoyl-3-trimethylammonium-propane (DOTAP), 1,2-dioleoyl-*sn*-glycero-3-phosphoethanolamine (DOPE), 1,2-dioleoyl-*sn*-glycero-3-phosphoethanolamine-N-[carbonyl-methoxy (polyethylene glycol)-2000] (DOPE-mPEG_2000_) and 1,2-distearoyl-*sn*-glycero-3-phosphoethanolamine-N-[carboxy (polyethylene glycol)-2000] (DOPE-PEG_2000_-COOH) were purchased from Aladdin (Shanghai, China). 2-Morpholinoethanesulphonic acid (MES), superoxide dismutase (SOD) activity assay kit, Ca^2+^ fluorescence probe assay kit, ROS assay kit, terminal deoxynucleotidyl transferase dUTP nick-end labeling (TUNEL) cell apoptosis assay kit, calcein acetoxymethyl ester/propidium iodide (Calcein-AM/PI) cell double staining kit, cell apoptosis analysis kit, 5,5′,6,6′-tetrachloro-1,1′,3,3′-tetraethyl-imidacarbo-cyanine iodide (JC-1) mitochondrial membrane potential assay kit, the defatted milk powder, lipopolysaccharide (LPS), phenylmethanesulfonylfluoride (PMSF), 3,3′-dioctadecyloxacarbocyanine perchlorate (DiO), 1,1′-dioctadecyl-3,3,3′,3′-tetramethylindocarbocyanine perchlorate (Dil), Alexa Fluor 488 secondary antibody, Hoechst 33342 and 2-(4-Amidinophenyl)-6-indolecarbamidine dihydrochloride (DAPI) were purchased from Beyotime Biotechnology Co. Ltd. (Shanghai, China). The FITC-*anti*-mouse CD206 (141703) was purchased from Biolegend (Beijing, China). The tumor necrosis factor alpha (TNF-α), arginase 1 (Arg-1), interleukin 10 (IL-10) and interleukin 6 (IL-6) enzyme-linked immunosorbent assay (ELISA) Kit were purchased from Meimian Industrial Co., Ltd (Jiangsu, China). Edaravone (Eda) was purchased from MedChemExpress LLC. The 2,3,5-triphenyltetrazolium hydrochloride (TTC, 2 %) and anti-caspase 8 (K001600P) was purchased from Solarbio Science & Technology Co., Ltd (Beijing, China). The Smart PAGE Precast Protein Gel was purchased from Smart-Lifesciences Biotechnology Co., Ltd (Changzhou, China). Anti-β-actin (SB-AB0035) was obtained from Sharebio. Anti-caspase 1 (31020-1-AP), anti-RIPK1 (17519-1-AP) and PE-*anti*-mouse CD86^+^ (PE-65068) were obtained from Proteintech Group. Anti-NOD-like receptor thermal protein domain associated protein 3 (NLRP3) (A24294) and anti-TMS1/ASC (A22046) were obtained from ABclonal.

### 2.2 Synthesis of USPIO

USPIO nanoparticles were synthesized via thermal decomposition assay with minor modification [44]. A mixture containing FeCl_3_·6H_2_O (1.6 g, 6 mmol), sodium oleate (6.1 g, 20 mmol), ethanol (15 mL), D.I. water (10 mL) and hexane (20 mL) was heated at 70 °C for 4 h with vigorously stirring. The resulting organic layer was washed with D.I. water and then evaporated to obtain the iron-oleate complex. Iron-oleate complex (2.7 g, 3.0 mmol), oleic acid (0.8 g, 3.0 mmol), and oleyl alcohol (2.4 g, 9.0 mmol) were then dissolved in diphenyl ether (30 mL) and heated to 250 °C with a heating rate of 5 °C/min. The mixture was rapidly cooled down to room temperature. 5 mL chloroform and 10 mL acetone were added into the mixture and then centrifuged at 5000 rpm for 10 min to obtain USPIO with a size of 3–5 nm.

### 2.3 Synthesis of DOPE-PEG_2000_-TEMPO

TEMPO-NH_2_ was linked to the surface of liposomes using EDC and Sulfo-NHS. Then 50 mg DOPE-PEG_2000_-COOH was dispersed in 1 mL MES buffer (pH = 6). And then 1 mL MES solution including EDC (9.68 mg) and NHS (2.7 mg) was added to activate the DOPE-PEG_2000_-COOH at 37 °C for 1 h. Next, TEMPO-NH_2_ (31.26 mg) dissolving in MES was added to the mixture with further stirring for 24 h, followed by dialysis (MWCO 1000 Da) against distilled water to remove redundant TEMPO-NH_2_. The final solution in the dialysis bag was lyophilized and stored at 4 °C for future use. The product was characterized by proton nuclear magnetic resonance (^1^H NMR) spectroscopy on a Bruker AVANCE-III spectroscope with CDCl_3_ as a solvent.

### 2.4 Synthesis of ULips

Liposomes were prepared according to the method described by Zijian Zhou et al. with slight modification [45]. Briefly, lipid components DOPE, DOPE-PEG_2000_-TEMPO and positively charged lipid DOTAP and USPIO were mixed in chloroform, and a molar ratio of lipid components was 1:0.12:1. Chloroform was evaporated under a vacuum for 40 min. Then, lipids were dispersed in PBS at pH 7.4. The solution was vortexed for 2 min. After homogenization in an ultrasonic bath for 20 min at 4 °C, membrane structure of liposomes was formed. Lips, without USPIO, were prepared using the same method. Characterization of the size and zeta potential distribution of two liposomes was conducted by transmission electron microscopy, dynamic light scattering and electrophoretic light scattering.

### 2.5 Characterization of USPIO and ULips

Transmission electron microscopy (TEM, JEM-1200EX, Japan) was used to observe the structure and morphology of ULips. Scanning electron microscope (SEM, JEM-3200FS, JEOL, Japan) were utilized to obtain morphological images of MA@ULips. And the morphology of MA@ULips were also determined by a Bio-TEM (JEOL, JEM-1400 Flash, Japan). Elemental composition of USPIO was analyzed by X-ray photoelectron spectroscopy (XPS, Thermo Scientific, USA). The crystal phase of USPIO was analyzed by X-ray diffraction (XRD, Smartlab, Tokyo, Japan). The size distributions and zeta potentials of Lips and ULips were determined by dynamic light scattering (DLS, Malvern, NanoZS, U.K.).

### 2.6 Preparation and characterization of MA@ULips

MA@ULips was obtained by incubating RAW 264.7 cells with ULips. After macrophages adhered to the wall, the medium was exchanged with serum-free Dulbecco's Modified Eagle Medium (DMEM). ULips (200 μg/mL quantified by lyophilization and all used ULips concentrations are expressed in terms of total nanoparticle mass concentration) was added to macrophages for 2 h and then placed in a cell culture incubator. After washing, MA@ULips was obtained and used immediately for the subsequent study. To determine the optimal formulations of MA@ULips, cell viability and integration efficiency were used as indices to investigate the incubation concentration (400, 200, 100, 50 and 25 μg/mL quantified by ULips) and time by a single-factor study. Macrophages were treated by ULips with 5, 10, 20, 30, 45, 60 and 120 min by flow cytometry to observe the optimal incubation time. The cell viability of ULips was tested by cell counting kit-8 (CCK-8) assay. Macrophages were seeded in 96-well plates. DMEM medium without fetal bovine serum (FBS) was used to dilute ULips and the concentrations of ULips in each well were 400, 200, 100, 50 and 25 μg/mL. Plates were further incubated for 24 h. Then, excess ULips was washed away using DMEM without FBS. Then, 10 μL of CCK-8 and 100 μL of serum-free DMEM was added after incubation. A microplate reader was used to measure cell viability at 450 nm. For membrane fusion mechanism validation, RAW 264.7 cells were seeded in a confocal dish with approximately 80 % confluence, followed by pretreating with DMEM containing inhibitors as follows: thylisopropylamiloride (EIPA) (30 μM), methyl-β-cyclodextrin, (MβCD) (5 mM), and dynasore (80 μM) at 37 °C for 1 h. The PBS group was set as the control. After pretreatment, the cells of all groups were washed with PBS and further incubated with ULips-Dil for 2 h. The mean fluorescence for each group was analyzed using a flow cytometer. Additionally, DiO was used to stain the membrane of RAW 264.7 cells. Then the RAW 264.7 cells were washed with PBS and further incubated with ULips-Dil for 2 h. Next, cells were washed with PBS three times, while the nuclear was stained with DAPI. Confocal laser scanning microscopy (CLSM) images were captured using the excitation wavelengths of 405 nm for DAPI, 484 nm for DiO, and 549 nm for Dil.

### 2.7 SOD-like activity of ULips

The SOD-like activity of ULips was assessed using a total SOD activity assay kit based on the 2-(2-methoxy-4-nitrophenyl)-3-(4-nitrophenyl)-5-(2,4-disulfophenyl)-2H-tetrazolium (WST-8) method (Beyotime, China) following the manufacturer's instructions. Different concentrations of ULips (100–800 μg/mL) were added to the working solution, and absorbance at 450 nm was measured using a microplate reader.

### 2.8 Catalase (CAT)-like activity of ULips

The CAT-like ability was evaluated using a dissolved oxygen meter to monitor the concentration of O_2_ generated in solution. Kinetic assays were performed by adding 500 μL of ULips to 5 mL of _hydrogen peroxide (H2_O_2_) solutions at varying concentrations (31.25, 62.5, 125, 250 and 500 mM). The dissolved O_2_ concentration was continuously recorded over 600 s (n = 3 for each group).

### 2.9 •OH radical scavenging assay of ULips

The •OH-scavenging capacity of ULips was evaluated using the 3,3′,5,5′-tetramethylbenzidine (TMB) method with a microplate reader. •OH was generated by the classical Fenton reaction between H_2_O_2_ and Fe^2+^, which oxidizes TMB into oxidized TMB (oxTMB), exhibiting a characteristic absorption at 652 nm. Working solutions containing 500 μM TMB, 4 mM H_2_O_2_, 2 mM FeSO_4_ and different concentrations of ULips (100–800 μg/mL) in HAc/NaAc buffer (pH 4.5) were prepared in the dark and incubated for 5 min. Residual •OH levels were quantified by measuring absorbance at 652 nm.

### 2.10 2,2′-azino-di (3-ethylbenzthiazoline-6-sulfonic acid) (ABTS) radical scavenging activity assay of ULips

The ABTS working solution was prepared by mixing equal volumes of ABTS (3.84 mg/mL) and potassium persulfate (1.34 mg/mL). This solution was then co-incubated with varying concentrations of ULips (100–800 μg/mL), and absorbance at 734 nm was measured using a UV–vis spectroscopy and a microplate reader.

### 2.11 2,2-Diphenyl-1-picrylhydrazyl (DPPH) radical scavenging activity assay of ULips

The DPPH radical scavenging activity was evaluated using a DPPH decolorization assay. ULips at different concentrations were mixed with a 3 mM DPPH solution in anhydrous ethanol and incubated for 30 min. Absorbance at 517 nm was recorded, and the radical inhibition rate was calculated from the reduction in absorbance relative to the control.

### 2.12 2-Phenyl-4,4,5,5-tetramethylimidazoline-1-oxyl 3-oxide (PTIO) radical scavenging activity assay of ULips

The PTIO radical scavenging capacity of ULips was assessed by incubating varying concentrations of ULips with an aqueous PTIO solution (3 mg/20 mL) at 37 °C for 2 h. The total reaction volume was adjusted with distilled water, and absorbance was measured at 557 nm.

### 2.13 Cell culture

Phaeochromocytoma (PC12) cells, mouse microglia (BV2), mouse peritoneal macrophages (RAW 264.7) and mouse brain microvascular endothelial cells (bEnd.3) were acquired from Procell Life Technology Co., Ltd. All cell lines were authenticated by short tandem repeat (STR) profiling prior to the experiment, and mycoplasma testing confirmed that they were contamination-free. RAW 264.7, BV2 and bEnd.3 cells were cultured in DMEM supplemented with 10 % FBS and 1 % penicillin/streptomycin at 37 °C in a 5 % CO_2_ atmosphere. The PC12 cells were cultured in Roswell Park Memorial Institute (RPMI) 1640 with 10 % FBS and 1 % penicillin/streptomycin at 37 °C in a 5 % CO_2_ atmosphere.

### 2.14 Cell viability

To verify the cytotoxicity of MA@ULips, PC12, BV2 and bEnd.3 cells were planted in bottom chamber of 48-well plate and the culture was continued for 24 h. Then MA@ULips containing different concentrations of ULips were planted in upper chamber. Last, the medium in the plates were removed, and 500 μL of CCK-8/DMEM [1/9 (v/v)] solution was added. After 1 h of incubation, the absorbance was measured at the wavelength of 450 nm using a microplate reader, and cell viability was calculated.

### 2.15 Inflammatory migration in vitro

For inflammatory migration assay, bEnd.3 cells were seeded on the lower chamber of six-well transwell plates (3 μm pore size, Corning Incorporated). After incubation for 24 h, the bEnd.3 cells were exposed to H_2_O_2_ of 1 mM and these groups were used as treatment groups. The bEnd.3 cells exposing to fresh serum-free media were used as control group. After 6 h, 1 mL MA@ULips (1 × 10^6^ cells/mL) suspending in fresh serum-free media were placed in the upper chamber. After incubating for different time, cells that have not migrated to the lower chamber were removed using a cotton swab, whereas the cells that migrated to the lower membrane surface were fixed, stained with 0.1 % crystal violet and viewed by light microscope.

### 2.16 Detection of MA@ULips to cross BBB

First, bEnd.3 cells were cultured in a transwell system. When bEnd.3 cells adhered tightly together and the *trans*-endothelial electrical resistance reached 150 Ω•cm^2^, BBB formation was successful. H_2_O_2_ was used to stimulate the inflammation environment of brain cells. PC12 cells were cultured in the lower compartment of the culture plate and treated with H_2_O_2_ (1 mM). After conducting the H_2_O_2_ injury for 12 h, ULips and MA@ULips were added to the upper chamber of transwell. The fluorescence intensity of DiO (green) in PC12 cells was detected by CLSM after 6 h. The bEnd.3 cells were labeled with iFluor™ 488 phalloidin and nuclei of PC12 cells were labeled with DAPI (blue). The fluorescence intensity of the lower chamber of each group was observed by CLSM.

### 2.17 Evaluation in cellular protecting from oxidative damage

PC12 cells were cultured in Roswell Park Memorial Institute (RPMI) 1640 supplemented with 10 % FBS and 1 % penicillin/streptomycin at 37 °C in a 5 % CO_2_ atmosphere. Cells were seeded in the lower chamber of transwell-equipped 6-well plates (3 × 10^5^ cells/well). After 24 h, the medium was replaced with fresh medium containing H_2_O_2_ (1 mM), and MA@ULips (1 × 10^5^/well) were seeded into the upper chamber. The cells were incubated for an additional 24 h, after which cell viability was measured using the CCK-8 assay (n = 3 for each group).

### 2.18 Evaluation of ROS scavenging in PC12 cells

PC12 cells were seeded into a confocal cell culture dish at a density of 5 × 10^4^ cells per well and incubated overnight. Then, the cells were treated by the following three groups for 12 h: PBS, H_2_O_2_ (1 mM) and MA@ULips. Intracellular ROS levels of PC12 cells were analyzed with 2′,7′-dichlorodihydrofluorescein diacetate (DCFH-DA). Next, the cells were incubated with 10 μM DCFH-DA for 30 min and then washed three times with PBS. At last, intracellular DCF fluorescence images were acquired by CLSM. Furthermore, ROS was measured by flow cytometry and the flow data were analyzed by FlowJo software.

### 2.19 Cell apoptosis and mitochondrial membrane potential assays

PC12 cells were seeded in 12-well culture plates at a density of 3 × 10^4^ cells per well and incubated overnight. Then, the cells were treated by the following three groups for 12 h: PBS, H_2_O_2_ (1 mM) and MA@ULips. Then, cells were processed into suspension cells, stained with Annexin V and PI, and collected for flow cytometry to evaluate the cell apoptosis. Furthermore, TUNEL staining was also used for detecting cell apoptosis under the CLSM. For detection of mitochondrial membrane potentials, the cells were incubated with JC-1 reagent for 20 min and then tested by flow cytometry and CLSM.

### 2.20 Live/dead cell staining experiment and calcium staining

PC12 cells were seeded into a confocal cell culture dish at a density of 5 × 10^4^ cells per well and incubated overnight. Then, the cells were treated by the following three groups for 24 h: PBS, H_2_O_2_ (1 mM) and MA@ULips. After incubation, the medium was removed and the cells were washed three times with PBS. Finally, the cells were co-stained with Calcein-AM/PI, and CLSM was used to observe the cell survival. For calcium staining by Fluo-8 AM, the cells were collected and stained using Fluo-8 AM staining kit, and then observed by CLSM. At the same time, flow cytometry was used to detect intracellular calcium.

### 2.21 Western blot analysis

To evaluate the expression of critical differentially expressed genes in various groups, BV2 cells were activated by LPS (1 μg/mL, 24 h) or Oxygen Glucose Deprivation/Reoxygenation (OGD/R) (6 h) to obtain proinflammatory M1 microglia. LPS or OGD/R-stimulated BV2 cells were further treated with MA@ULips for 24 h to obtain anti-inflammatory microglia, with normally cultured BV2 cells as the control group. The total amount of protein in each sample was determined with a bicinchoninic acid (BCA) assay (QuantiPro BCA Assay Kit, Sigma-Aldrich). Equal amount of protein in each sample was separated by SDS-polyacrylamide gel electrophoresis and transferred to polyvinylidene fluoride membranes (PVDF). After being blocked with defatted milk powder, membranes were incubated with primary antibodies and stored at 4 °C overnight, followed by incubation for 1 h at room temperature with the anti-rabbit secondary antibodies (1:4000, Abclonal, Wuhan, China). Finally, immunoreactivity was measured using the Image Lab™ software.

### 2.22 In vitro evaluation of regulation of microglial polarization

BV2 cells were seeded into a confocal cell culture dish at a density of 5 × 10^4^ cells per well and incubated overnight. After the cells stuck to the wall, the cells were treated by the following three groups for 24 h: PBS, LPS (1 μg/mL) and MA@ULips. The cells were washed with PBS twice and fixed with 4 % paraformaldehyde for 15 min. Then, the cells were exposed to a membrane breaking fluid (1 % Triton) for 30 min, and washed with PBS three times (5 min each time). After the cells were blocked with blocking buffer (3 % bovine serum albumin in tris-buffered saline solution) at 37 °C for 1 h, then cells were incubated with CD206 primary antibody. They were incubated with the corresponding diluted antibody overnight in the dark at 4 °C. Then, the cells were washed with PBS three times and incubated with diluted fluorescent dye-linked secondary antibody for 1 h in the dark at 37 °C, followed by washing with PBS three times again, and immersing in DAPI (0.5 μg/mL) for 10 min. The fluorescence images were observed using CLSM after the cells were washed with PBS three time. For in vitro M1/M2 polarization of microglia, BV2 cells were seeded in six-well plates at a density of 2 × 10^6^ cells per well. Then, the cells were treated by the following three groups for 24 h: PBS, LPS (1 μg/mL) and MA@ULips. Finally, the cells were stained with antibodies (surface markers CD86 and CD206) for the flow cytometer. Morphological changes of BV2 cells were observed in a bright field by a Leica inverted fluorescence microscope. Last, after being cultured with three treatments for 24 h, the supernatant of cells was collected. IL-6, IL-10, TNF-α and Arg-1 were verified, using an ELISA assay of inflammatory cytokines. The specific processes were performed according to the manufacturer's protocols.

### 2.23 OGD/R model

The PC12 and BV2 cells were cultured at 37 °C for 24 h. The cells were then washed with PBS, and the culture medium was then replaced with glucose-free DMEM. The cells were cultured in a sealed chamber equipped with an AnaeroPack (Mitsubishi Gas Chemical, Tokyo, Japan) to maintain an anaerobic atmosphere. The cells were reoxygenated and supplemented with complete DMEM medium after OGD/R for 4 or 6 h.

### 2.24 Biosafety assay in vivo

C57BL/6 mice (male, 25 g) were obtained from the Shanghai Leigenda Technology Co., Ltd in China. All mice were housed under standard conditions (23 ± 2 °C; 55 % ± 5 % humidity) with a 12 h light-dark cycle. All experimental procedures were carried out in accordance with the guidelines in “the Animal Management Regulations” of the Ministry of Health of the People's Republic of China and were approved by the Experimental Animal Ethics Committee at Fudan University ( 202306001S). 20C57BL/6 mice were randomly divided into three groups (0, 7, 21 and 35 days) (n = 5). The MA@ULips (1 × 10^6^ cells/100 μL) was injected into each mouse. Then, all mice were sacrificed at predetermined time points. The blood samples of each mouse were collected for biochemistry assay and routine blood examination. The major organs (heart, liver, spleen, lung, kidneys and brain) of the mice were harvested and stained with hematoxylin and eosin (H&E) for histological analysis.

### 2.25 In vivo establishment of transient middle cerebral artery occlusion/reperfusion (MCAO/R) model

C57BL/6 mice (6–8 weeks, male) were used to establish a MCAO/R model according to the reported protocols. Mice were anesthetized with 4 % chloral hydrate. Mice were fixed to the operating table and the fur in their neck was shaved by a razor. The left common carotid artery (CCA), external carotid artery (ECA) and internal carotid artery (ICA) on one side were exposed and separated under sterile conditions. Next, the CCA was ligated to block the blood flow, then a small incision was cut at the distal end of the CCA. Then, a silicon-coated nylon monofilament (0.22 mm in diameter) was carefully inserted into the ICA from the small incision in the CCA to block the blood flow of left middle cerebral artery. After a certain period, the monofilament was pulled to allow reperfusion.

### 2.26 The inclusion and exclusion criteria

For this study, the inclusion criteria were defined as follows: Longa neurological function score ≥2 at 24 h postoperatively, and TTC staining confirming an infarct volume between 20 % and 50 % of the total brain volume. Exclusion criteria included death within 24 h post-surgery, neurological function score <2, or evidence of infection or abnormal spontaneous activity.

### 2.27 In vivo stability of MA@ULips

Cyanine 5.5 (Cy5.5)-NH_2,_ a fluorescent analogue, was employed as a surrogate for TEMPO-NH_2_. And DOPE-PEG_2000_-Cy5.5 was synthesized using the same conjugation method as that employed for DOPE-PEG_2000_-TEMPO. DOPE-PEG_2000_-Cy5.5 was intravenously administered to mice. Blood samples were collected at 1 h, 4 h, 8 h, and 12 h post-injection, followed by centrifugation to obtain plasma. Plasma samples were then subjected to ultrafiltration to retain DOPE-PEG_2000_-Cy5.5, with the filtrate containing free Cy5.5. Subsequently, the fluorescence intensity of the filtrate was quantified.

### 2.28 The targeting and distribution analysis in vivo

ULips and MA@ULips were labeled with Cy5.5 (denote as ULips-Cy5.5 and MA@ULips-Cy5.5, equivalent to 0.5 mg/kg of Cy5.5) and injected into mice. After 2, 4, 6, 8, 12 h after injection, mice of different treatments were imaged by a small animal imaging system. To more preciously observe the distribution of MA@ULips in cerebral ischemic lesions, MCAO/R mice were injected with fluorescent DiR-labeled MA@ULips (denote as MA@ULips-DiR, equivalent to 0.1 mg/kg of DiR) at 0.5 h post ischemia. At 6 h and 12 h post-injection, brains and other major organs including heart, liver, spleen, lung and kidneys were taken. For brain distribution, an in vivo animal imaging system was used to observe and quantify the fluorescence distribution in the brains and other major organs of mice.

### 2.29 The evaluation of therapeutic effect in MCAO/R mice model

Mice were randomly divided into five groups (sham, saline, Eda, ULips, and MA@ULips). For later four groups, mice were treated by MCAO/R process. MCAO/R mice subjected to 60 min ischemia were intravenously injected with normal saline, Eda (3 mg/kg), ULips (3 mg/kg) and MA@ULips (1 × 10^6^ cells/100 μL, 3 mg/kg ULips), respectively. To assess the treatment effect, the brain sections were stained by TTC (2 %) at 24 h post-reperfusion. Infarct volumes of the animal models were measured by ImageJ software. Neurological deficit scoring method was used for behavior evaluation at 24 h post-surgery as follows: 0, no deficit; 1, flexion of contralateral forelimb upon lifting of the whole animal by the tail; 2, circling to the contralateral side; 3, falling to contralateral side; 4, no spontaneous motor activity; 5, death. The body weight and survival rate were assessed every day after drug administration. Representative behavioral tests including the open field test, cylinder test and modified neurological severity score (mNSS) were conducted on the third day after the preparation of the MCAO/R animal model. The mNSS scores range from 0 (normal) to 18 (most severe) for movement, sensation, balance, and reflexes.

### 2.30 Open field test (OPT)

On the third day after conducting the MCAO/R animal model, OPT experiments were conducted, and mice were placed in square fields. These mice can freely walk inside, recording time and walking routes. The exercise time for each mouse was 10 min. All data during the testing period were recorded and analyzed through a video tracking system. There were three mice in each group.

### 2.31 MRI test

In order to test the degree of cerebral edema after different treatment, at day 1 after the surgery, T_2_-weighted sequences were detected in 3.0 T MRI apparatus (UIHMR870). After anesthesia, mice were fixed on an MRI scanner operating bed. T_2_ parameters were as follows: repetition time: 1000 ms, echo time: 97.5 ms, layer thickness: 3 mm, field of view: 120 × 120 mm, and image matrix: 96 × 128. Moreover, T_2_ weighted sequences were also used to observe the dynamic change of inflammation in ischemic brain. After MCAO/R model, mice were injected with MA@ULips, and at 0, 0.5, 1, 2 and 3 h, mice underwent T_2_-weighted scan. The scanning parameters were the same as above. And ImageJ software was used to evaluate the signal intensity around the ischemic site in T_2_-weighted images.

### 2.32 Immunofluorescence and immunohistochemistry staining

The brain tissue of mice was taken out after anesthesia and dealt with 10 % formalin and ethanol dehydration and then embedded in paraffin. Then brain slices were incubated with anti-CD86/CD206 antibody (1:400), anti-TUNEL antibody (1:400), anti-microtubule-associated protein 2 (MAP2) antibody (1:800), anti-neuronal nuclei (NeuN) antibody (1:400), anti-ionized calcium-binding adapter molecule 1 (Iba-1) antibody (1:200) at 4 °C overnight and washed with PBS for three times, and then incubated with Alexa Fluor-conjugated secondary antibody (1:400) for 2 h at room temperature. DAPI was used as a nuclear stain. And sections were stained with Nissl, H&E, caspase-1 and glial fibrillary acidic protein (GFAP). All sections at a similar coronal position are observed under a Virtual Digital Slice Scanning System.

### 2.33 Blinding and randomization

Experimental animals were randomized after acclimatization using a computer-generated sequence. Research assistant who was not involved in any subsequent procedures assigned eligible animals to each group based on this sequence, ensuring that baseline characteristics did not differ significantly between groups. For blinding during surgery, the researcher performing the MCAO/R operation was not involved in the randomization process and remained unaware of the group assignments of the animals. For blinding during MRI analysis, TTC quantification, immunohistochemistry and behavioral scoring, all quantitative image analyses, evaluations of tissue staining and assessments of behavioral videos were conducted by researchers blinded to the group information. All images and data files used for analysis were labeled with anonymous codes.

### 2.34 RNA-seq analysis

At 72 h after MCAO/R, brain tissue samples located at the ischemic stroke site were sent to Hangzhou cosmos wisdom Biotechnology Co. Ltd. (Hangzhou, China) for purification, reverse transcription, library construction and sequencing. Data quality was assessed by the FastQC tool after removal of adaptor sequences, ambiguous “N” nucleotides (proportion of “N” > 5 %), and low-quality sequences (quality score <10). Difference was statistically significant threshold for |log_2_ fold change| ≥ 1 or *P* < 0.05. GO functional enrichment and KEGG pathway analysis were carried out by Goatools and KOBAS, respectively.

### 2.35 Statistical analysis

All data were analyzed by Graphpad prism 8 (GraphPad Software). Data were expressed as the mean ± standard deviation (SD). The statistical significance of the data was assessed using one-way analysis of variance (ANOVA) with Tukey's multiple comparisons test. The level of significance in the statistical analyses was defined as ∗*p* < 0.05, ∗∗*p* < 0.01, ∗∗∗*p* < 0.001, and ∗∗∗∗*p* < 0.0001.

## 3 Results and discussion

### 3.1 Preparation and characterization of MA@ULips

**Fig. 1:**
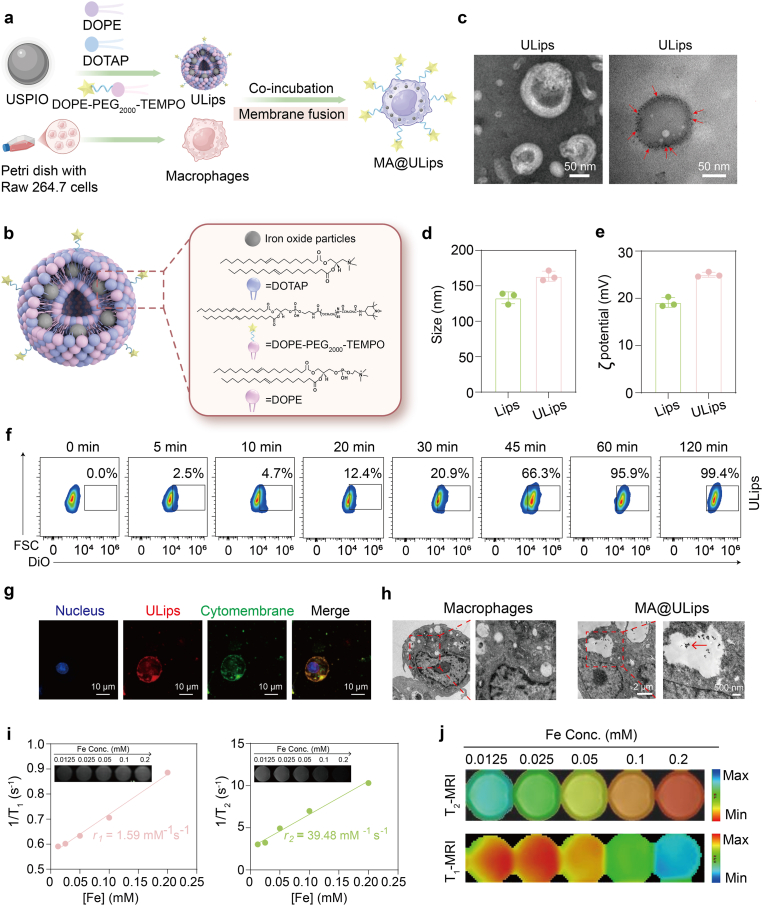
Preparation and characterization of MA@ULips. (a) Schematic illustration of the preparation of MA@ULips. (b) Schemes showing the composition and structural formula of ULips. (c) TEM images of ULips, in which the TEM image on the left shows negatively stained ULips using 1 % phosphotungstic acid and TEM image on the right shows positively stained ULips. (d) Size distributions of Lips and ULips (n = 3). (e) Zeta potentials of Lips and ULips (n = 3). (f) Flow cytometry of ULips internalization. (g) CLSM images of DiO labeled macrophages after incubation with Dil labeled ULips. (h) Bio-TEM images of macrophages and MA@ULips. (i) The longitudinal relaxation rate *r*_1_ and transverse relaxation rate *r*_2_ of the MA@ULips. (j) T_1_ and T_2_ color coded maps of MA@ULips. Data in (d) and (e) are presented as mean ± standard deviation (SD).

The USPIO nanoparticles were synthesized using thermal decomposition method with minor modification as the foundational component of our study. Following this, the USPIO nanoparticles were encapsulated with a fusogenic lipid shell comprising DOPE, DOTAP, DOPE-PEG_2000_-TEMPO (PEG2000) at a molar ratio of 1:1:0.12 using a film hydration method, resulting in the formation of USPIO-loaded liposomes (ULips) (Fig. 1a and b and S1). The engineered ULips were designed to fuse with the macrophage cell membranes, creating a construct termed MA@ULips. The USPIO nanoparticles are characterized by TEM, which confirms that the nanoparticles feature a uniform size range of 3–6 nm (Fig. S2). The XRD pattern exhibits distinct diffraction peaks that can be indexed to the γ-Fe_2_O_3_ crystal phase (Fig. S3), confirming the successful synthesis of phase-pure maghemite with high crystallinity. And the composition of the USPIO nanoparticles is further corroborated though XPS, which identifies the presence of iron (Fe), oxygen (O), and carbon (C) within the survey spectrum. Notably, the binding energies for Fe 2p_3/2_ and Fe 2p_1/2_ are determined to be 710.5 and 724.3 eV, respectively (Fig. S4), confirming the successful synthesis of γ-Fe_2_O_3_ nanoparticles.

Specifically, Fourier transform infrared (FT-IR) spectroscopy further supports the structural integration of the TEMPO moiety within the lipid conjugate. A newly observed characteristic absorption band at 1360 cm^−1^ corresponds to the N–O stretching vibration of the TEMPO group (Fig. S5), thereby confirming its successful conjugation. This finding is in agreement with ^1^H NMR results (Fig. S6), which collectively validate the formation of DOPE-PEG_2000_-TEMPO. ULips exhibit a well-defined spherical morphology, with negatively stained TEM image showing a distinct lipid bilayer surrounding the USPIO core. USPIO nanoparticles are incorporated within the phospholipid bilayer (Fig. 1c). The hydrodynamic diameter increases from 133.0 nm for control liposomes (Lips) to 163.6 nm for ULips (Fig. 1d and S7), indicating successful encapsulation. Concurrently, the zeta potential shifts from 19.1 mV for Lips to 25.0 mV for ULips (Fig. 1e), suggesting the enhanced stability.

To evaluate the cytotoxicity of ULips on macrophage cells, we assessed cell viability across a concentration range of 0–400 μg/mL, observing that cell viability remains above 90 %, indicating negligible toxicity (Fig. S8). In addition, viability staining with Calcein-AM and PI also confirms the biocompatibility of ULips with macrophages, as no obvious red fluorescence, which is characteristic of necrotic cells, is observed (Fig. S9). Fusion efficiency was evaluated over time, revealing a peak fusion rate of 99.4 % at 2 h, representing the highest uptake efficiency observed across comparative liposome concentrations (Fig. 1f and S10). The morphology of macrophages post-incubation with ULips was examined by SEM (Fig. S11). No apparent structural alterations were detected on the macrophage surface, indicating that the fusion process preserves cellular morphology. To assess phenotypic alterations following membrane fusion, flow cytometry was employed to analyze the distribution of M1 and M2 macrophages (Fig. S12). While ULips treatment does not significantly affect CD206 expression, a modest decrease in CD86 expression is observed, suggesting a slight tendency toward M2 polarization. However, these changes are not sufficient to trigger a full phenotypic switch or produce biologically meaningful effects, indicating that ULips exerts minimal influence on macrophage polarization.

Furthermore, to investigate the formation of engineered macrophages via membrane fusion, ULips were labeled with Dil, while macrophage membranes were stained with DiO. CLSM images demonstrate that the ULips (red) primarily localize to the macrophage cell membrane and cytoplasmic regions (Fig. 1g and S13). Furthermore, cellular uptake of ULips was evaluated in the presence of well-known cell uptake inhibitors, including EIPA, MβCD and dynasore. None of these inhibitors significantly reduced ULips internalization (Fig. S14), indicating that membrane fusion, rather than endocytic mechanisms, mediates ULips uptake. In addition, biological transmission electron microscopy (Bio-TEM) confirms the internalization of USPIO nanoparticles within macrophages (Fig. 1h), while electron paramagnetic resonance measurements further validated the presence of paramagnetic TEMPO moieties embedded in the cell membrane (Fig. S15). These observations collectively indicate that ULips interact with macrophages through a membrane fusion mechanism, enabling the incorporation of the TEMPO-modified lipid shell into the cell membrane and facilitating the concurrent delivery of USPIO into the phospholipid bilayer of macrophages. To assess the potential of MA@ULips for MRI, in vitro measurements were conducted using a 3T MRI scanner. T_2_-weighted MRI phantoms exhibit progressively enhanced dark contrast for the MA@ULips. Notably, MA@ULips manifest a high *r*_2_ relaxation rate of 39.48 mM^−1^ s^−1^ and an *r*_2_/*r*_1_ ratio of 24.83 (Fig. 1i and j). USPIO nanoparticles typically exhibit excellent T_1_ imaging capabilities, the observed enhancement in T_2_ imaging performance for MA@ULips is likely attributed to the aggregation of USPIO nanoparticles within the liposomal structure, resulting in a transition from T_1_ to T_2_ relaxation dynamic.

### 3.2 ROS scavenging activity of ULips

**Fig. 2:**
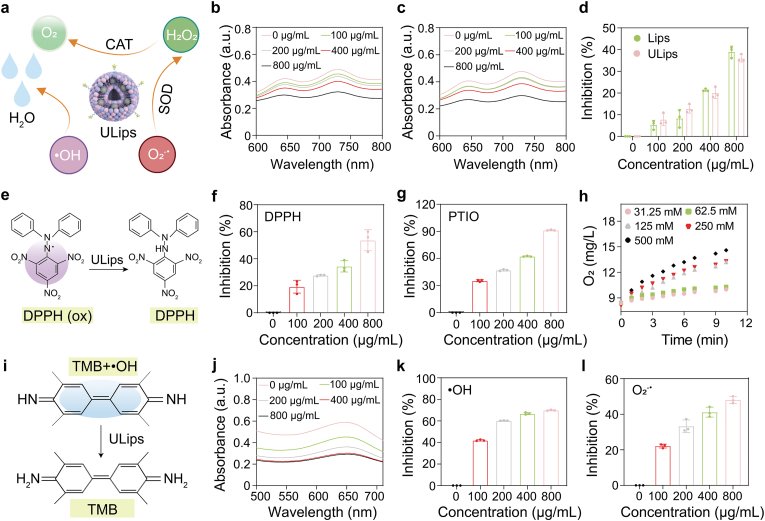
ROS scavenging ability of ULips. (a) Schematic illustration of multienzyme-like activities of ULips for ROS scavenging. Ultraviolet–visible (UV–vis) absorption spectra of ABTS in (b) Lips and (c) ULips groups. (d) Quantification analysis of antioxidative performance of ULips and Lips at different concentrations against ABTS^•+^ (n = 3). (e) Schematic representation depicting the reaction DPPH (ox) and ULips. (f) DPPH• scavenging ability of ULips at different concentrations (n = 3). (g) PTIO• scavenging ability of ULips at different concentrations (n = 3). (h) Time-dependent O_2_ generation in the presence of ULips at various H_2_O_2_ concentrations. (i) Mechanisms of TMB as a chromogenic indicator for •OH detection. (j) UV–vis absorption spectra of •OH elimination by ULips using TMB as chromogenic indicator. (k) •OH scavenging ability of ULips at different concentrations against (n = 3). (l) SOD-like activity of ULips (n = 3). Data in (d), (f), (g), (k) and (l) are presented as mean ± SD.

To comprehensively evaluate the antioxidant capabilities of ULips, the ROS-scavenging activities of ULips were explored (Fig. 2a). We first assessed their total antioxidant capacity using the ABTS assay, in which the ABTS radical ion (ABTS^•+^) exhibits a characteristic blue-green color (Fig. S16), which diminishes in the presence of antioxidants, indicating their high efficacy. The results indicate no significant difference in antioxidant performance between Lips and ULips (Fig. 2b–d), leading to focus subsequent experiments on ULips. The ROS-scavenging activity of ULips was further assessed using the DPPH assay, where the purple color of DPPH• gradually lightens upon exposure to an antioxidant (Fig. 2e). The results demonstrate a dose-dependent inhibition of DPPH•, with the scavenging rate increasing in relation to the concentration of ULips (Fig. 2f). To enhance the understanding of the ROS-scavenging capabilities of ULips, PTIO•, a utilized oxygen-centered radical, was utilized, indicating that ULips effectively eliminate over 90 % of free radicals at a concentration of 800 μg/mL (Fig. 2g).

In the context of inflammatory microenvironment, excessive ROS, particularly H_2_O_2_, pose significant threats due to their membrane permeability and detrimental effects on cellular and organelle functions [46]. CAT and CAT mimics exhibit exceptional capabilities in catalyzing the decomposition of H_2_O_2_ into O_2_ and H_2_O thereby protecting cells from oxidative damage [47]. We measured the CAT-like activity of ULips using a dissolved oxygen meter, which confirms an increase in O_2_ production correlating with elevated H_2_O_2_ concentrations (Fig. 2h and S17). Furthermore, the hydroxyl radical (•OH)-scavenging activity of ULips, employing TMB was the chromogenic substrate. The results reveal a significant decrease in the absorbance at 652 nm corresponding to oxTMB following treatment with ULips, underscoring the effective •OH scavenging capability of the liposomes (Fig. 2i–k). As a critical antioxidant enzyme, SOD plays a vital role in neutralizing superoxide anion radicals (•O_2_^−^) [48]. We subsequently investigated the SOD-like activities of ULips. In the experiment setup, •O_2_^−^ was generated by using xanthine (Xan) and xanthine oxidase (XOD), and the capacity of ULips to scavenge •O_2_^−^ was assessed through WST-8. The results demonstrate that approximately 50 % of the •O_2_^−^ is eliminated when treated with ULips at a concentration of 800 μg/mL (Fig. 2l). Collectively, these findings affirm that ULips possess a robust capacity for ROS scavenging, establishing a compelling foundation for their application in mitigating oxidative stress and conferring protective effects in biological systems.

### 3.3 Neuroprotective effects via ROS scavenging and inhibition of RIPK1-mediated PANoptosomes

**Fig. 3:**
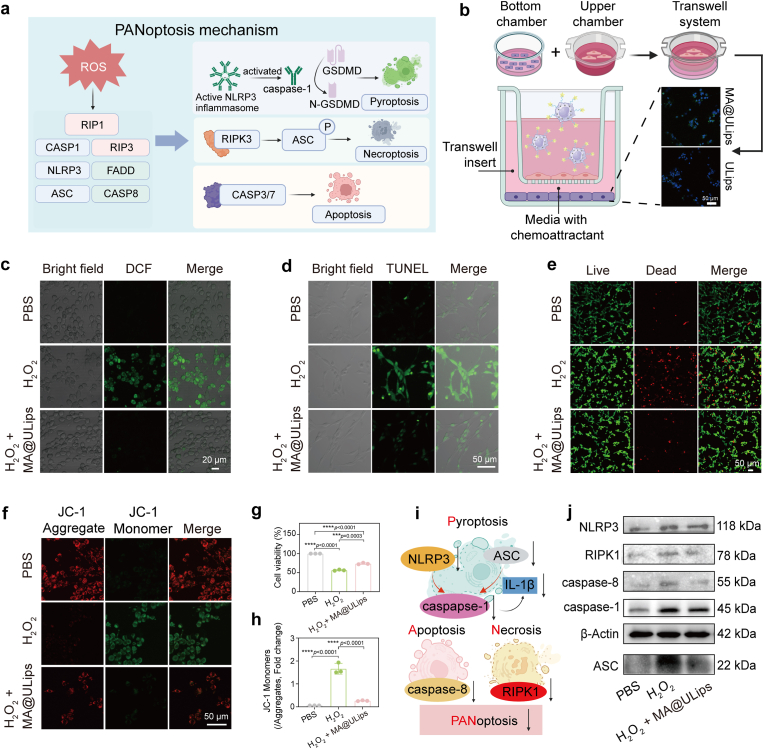
In vitro mechanism validation of MA@ULips-inhibited PANoptosis. (a) Diagram of PANoptosis pathway. (b) CLSM images of BBB penetration ability of MA@ULips. (c) CLSM images of intracellular ROS level in PC12 cells after different treatments. (d) CLSM images of cellular apoptosis in PC12 cells after various treatments. (e) CLSM images of live/dead staining PC12 cells after different treatments. (f) CLSM images of MMP in PC12 cells with various treatments staining by JC-1 probes. (g) The PC12 cell viability after various treatments (n = 3). (h) Corresponding flow cytometry quantification analysis of the JC-1 probe for detecting mitochondrial damage after various treatments in PC12 cells (n = 3). (i) Schematic diagram of MA@ULips-mediated inhibition of PANoptosis. (j) Western blotting analysis of PANoptosis-associated proteins in BV2 cells after various treatments. Data are presented as mean ± SD. Statistical significance was calculated by one-way ANOVA with Tukey's multiple comparisons test. ∗∗∗*p* < 0.001, ∗∗∗∗*p* < 0.0001.

Based on the desirable ROS-scavenging performance, MA@ULips are anticipated to provide protection to cells from oxidative damage. PANoptosis is generally considered to be an inflammatory programmed cell death regulated by the PANoptosome complex, which is characterized by activation of pyroptotic, apoptotic and necroptotic pathways [35] (Fig. 3a). To substantiate the inhibitory effects of PANoptosis of MA@ULips through ROS elimination, the in vitro coculture assay of MA@ULips and brain cells was employed for validation from multiple angles. Initially, the cytotoxicity of MA@ULips was assessed using CCK-8 assay. MA@ULips exhibit no significant toxicity across three different cell lines including PC12 cells, BV2 cells, and bEnd.3 cells, demonstrating the excellent cytocompatibility (Fig. S18), which is corroborated by Calcein-AM/PI staining (Fig. S19).

Following ischemic brain injury, lesion sites release abundant chemokines and inflammatory factors. Macrophages highly express receptors for these factors, including C–C chemokine receptor type 2 (CCR2) and C-X-C chemokine receptor type 4 (CXCR4) [49,50], and exhibit active chemotactic migration in an in vitro BBB model simulating ischemic stroke, enabling them to traverse the BBB and infiltrate ischemic brain tissues [51]. To verify the inflammation-targeting capability of MA@ULips, we established a transwell membrane system simulating an oxidative stress environment by treating bEnd.3 endothelial cells with H_2_O_2_ in the basolateral chamber. When the upper chamber contains fresh serum-free media, there is minimal migration of MA@ULips through the transwell membrane (Fig. S20). In contrast, the presence of H_2_O_2_ significantly increases the migration of macrophages, indicating that MA@ULips maintain their migratory and inflammatory response capabilities.

Furthermore, increased BBB permeability facilitates macrophage recruitment and promotes cellular migration. To further validate this hypothesis, a transwell model was used to simulate the BBB and assess its permeability. Under ischemic conditions, fluorescence is detectable in the lower chamber for both MA@ULips and dextran-FITC groups (Fig. S22a and b), indicating that MA@ULips can effectively exploit the transient increase in BBB permeability induced by ischemia. This enables BBB transport comparable to that of dextran-FITC, supporting effective brain targeting.

Subsequently, the transmigration and therapeutic efficiency of MA@ULips on neuronal cells were investigated. We constructed an in vitro BBB model using a transwell system, with bEnd.3 cell monolayer established in the upper chamber and PC12 cells in the lower chamber (Fig. 3b). After that, we stimulated an oxidative stress microenvironment with H_2_O_2_ and introduced ULips and MA@ULips into the upper chamber, respectively. CLSM was utilized to visualize BBB penetration, with bEnd.3 cells were stained with iFluor™ 488 phalloidin, to confirm barrier integrity (Fig. S21). The mean fluorescence intensity of DiO-labeled MA@ULips is higher than that of ULips, suggesting that MA@ULips effectively target inflammatory sites. In addition, colocalization analysis demonstrates that the green fluorescence in the MA@ULips group exhibits a more prominent distribution in the perinuclear region, whereas the fluorescence signal in the ULips group is remarkably attenuated (Fig. S22c and d).

To visualize intracellular ROS levels in PC12 cells, DCFH-DA was employed as a fluorescence probe (Fig. 3c and S23). Following H_2_O_2_ treatment, a pronounced increase in fluorescence is observed, indicative of elevated ROS production. However, pre-treatment with MA@ULips markedly reduces the intracellular ROS levels. Flow cytometric analysis confirms that MA@ULips treatment halves ROS levels in PC12 cells compared to the H_2_O_2_ group (Fig. S24). Cell death, especially apoptosis, represents a primary consequence of ROS-induced damage in ischemic conditions. We examined the anti-apoptotic effects of MA@ULips through flow cytometry, revealing a significant reduction in PC12 cell apoptosis induced by H_2_O_2_ (Fig. S25). TUNEL staining, which detects DNA fragmentation during apoptosis, further validates the anti-apoptotic capacity of MA@ULips, as evidenced by diminished green fluorescence post-treatment (Fig. 3d and S26). Additionally, viability staining with Calcein-AM and PI confirms a gradual increase in cell viability with MA@ULips treatment (Fig. 3e and S27). H_2_O_2_, as an inducer of oxidative stress, significantly reduce PC12 cell viability to 56.3 % (Fig. 3g). Conversely, treatment with MA@ULips could preserve cell viability above 73.7 %, demonstrating their protective effect against H_2_O_2_-mediated damage.

Given that mitochondria are critical for energy metabolism and highly susceptible to ROS imbalance, we assessed mitochondrial membrane potential (MMP) as a measure of mitochondrial health. Coculturing with MA@ULips significantly enhances the MMP of H_2_O_2_-treated PC12 cells, indicated by a transition from green to red fluorescence (Fig. 3f and S28). Flow cytometry analysis corroborates this finding, showing an increase in red fluorescence-positive PC12 cells from 39.2 % in H_2_O_2_ group to 78.4 % in the MA@ULips group (Fig. 3h and S29). Moreover, the concentration of ionized Ca^2+^ serves as an important indicator of ischemia-induced oxidative stress. Fluo-8 staining demonstrates a substantial increase in Ca^2+^ levels from 1.0 % to 28.8 % following H_2_O_2_ treatment. Remarkably, treatment with MA@ULips reduces Ca^2+^ levels to 3.8 % (Fig. S30). Corresponding CLSM images exhibit a reduction in Fluo-8 fluorescence, indicating neuroprotection conferred by the TEMPO moiety (Fig. S31).

Various forms of cell death, including apoptosis, pyroptosis and necroptosis, are implicated in ischemic stroke (Fig. 3i). Apoptosis is the most extensively studied form of cell death, characterized by a controlled process facilitated by caspase activation [52]. Notably, caspase-8 serve as a molecular marker of apoptosis. Treatment with MA@ULips effectively diminishes caspase-8 expression (Fig. 3j and S32), suggesting their protective role during early apoptotic stages. In addition to apoptosis, we investigated the expression of pyroptosis-associated proteins, noting significant upregulation of caspase-1, apoptosis-associated speck-like protein containing a caspase recruitment domain (ASC) and NLRP3 following H_2_O_2_ exposure. MA@ULips effectively inhibit the expression of these key indicators of pyroptosis. Furthermore, necroptosis, another form of cell death, is mediated by the activation of RIPK1, which along with RIPK3, and mixed lineage kinase domain-like protein (MLKL), forms necrotic bodies and contributes to inflammatory responses. Under hypoxic and ischemic conditions, the excessive accumulation of ROS in neural cells serves as a primary trigger for PANoptotic cell death. Specifically, elevated ROS levels activate RIPK1, which initiates the assembly of the RIPK1-associated PANoptosome. This multiprotein complex integrates central components of pyroptosis (NLRP3), apoptosis (caspase-8), and necroptosis (RIPK1/MLKL), thereby orchestrating the execution of PANoptosis. Our analysis of RIPK1 expression in BV2 cells demonstrates a significant downregulation in the MA@ULips group compared to the H_2_O_2_ group.

To further investigate the neuroprotective efficacy of MA@ULips, an OGD/R model was employed using PC12 cells. This model closely recapitulates key ischemic pathophysiology, including impaired energy metabolism, calcium overload, oxidative stress, and inflammation. To delineate the relative contributions of the therapeutic payload (TEMPO) and the liposomal carrier, we first introduced two additional in vitro control arms: free TEMPO and unloaded liposomes. PC12 neurons subjected to OGD/R exhibit a marked reduction in viability (Fig. S33a). Both MA@ULips and free TEMPO conferred significant cytoprotection relative to OGD/R and to the unloaded-liposome control, demonstrating that TEMPO is the principal mediator of the observed neuroprotective effect. Notably, MA@ULips preserve PC12 viability at levels exceeding 90 %, outperforming free TEMPO and indicating an added benefit of the integrated delivery system most likely attributable to improved cellular delivery and/or local retention of the antioxidant payload. These data establish the intrinsic cytoprotective activity of TEMPO, and the superior efficacy of the liposome-assisted, macrophage-engineered formulation. On the basis of these findings, subsequent experiments were streamlined to three groups including PBS, OGD/R, and MA@ULips. This focused design concentrates statistical power on the most relevant comparisons, reduces experimental complexity, and facilitates a clearer assessment of the comprehensive therapeutic performance of the MA@ULips platform.

Next, Calcein-AM/PI co-staining further confirms the improvement in cell viability, showing increased green fluorescence upon MA@ULips treatment (Fig. S33b). ROS levels were then evaluated using DCFH-DA staining. CLSM reveals strong green fluorescence in OGD/R-treated cells, indicative of ROS accumulation (Fig. S33c). MA@ULips treatment effectively suppressed ROS generation, confirming its antioxidative capacity. To further assess anti-apoptotic efficacy, we performed flow cytometric analysis of apoptosis. The apoptotic rate in OGD/R-injured cells reached 41.6 %, which was significantly reduced to 9.6 % following MA@ULips treatment (Fig. S33d and e), underscoring its potent anti-apoptotic effect. Given the high sensitivity of mitochondria to oxidative stress and their central role in ischemic injury, we evaluated mitochondrial membrane potential (ΔΨm) using the JC-1 probe. CLSM imaging demonstrates that control cells exhibit red fluorescence, indicative of intact ΔΨm (JC-1 aggregates), whereas OGD/R exposure leads to a shift toward green fluorescence (JC-1 monomers), reflecting mitochondrial depolarization (Fig. S33f and g). Notably, MA@ULips treatment reverses this depolarization, as evidenced by restored red fluorescence, indicating preservation of mitochondrial integrity.

To investigate the regulatory effects of MA@ULips on PANoptosis, Western blot analysis was performed for key markers including RIPK1, MLKL, caspase-1, caspase-3, NLRP3, and the cleaved N-terminal fragment of Gasdermin D (GSDMD-N). OGD/R significantly upregulates the expression of all these proteins (Fig. S33h), confirming PANoptotic activation. Treatment with MA@ULips significantly reduces the expression of these proteins. By concurrently inhibiting apoptotic, pyroptotic, and necroptotic pathways, MA@ULips achieve suppression of PANoptosis. Mechanistically, MA@ULips disrupt RIPK1-mediated PANoptosome assembly and inhibit the activation of the NLRP3 inflammasome complex (NLRP3, ASC, and caspase-1), thereby reducing cleavage of GSDMD and production of its pore-forming fragment GSDMD-N. This inhibition blocks interleukin-1 beta (IL-1β) and interleukin-18 (IL-18) maturation and release, effectively mitigating inflammatory cytokine efflux.

We further explored whether MA@ULips exert a direct regulatory effect on the RIPK1-PANoptosome axis. To test the hypothesis, we first performed co-immunoprecipitation analysis to evaluate the specific interactions among RIPK1, RIPK3 and ASC. RIPK3 and ASC are readily detected in anti-RIPK1 immunoprecipitates but are absent in the immunoglobulin G (IgG) control (Fig. S34a), confirming the formation of RIPK1-RIPK3-ASC complex. Notably, treatment with MA@ULips markedly attenuate these interactions. We then assessed the key activation marker phosphorylated RIPK1 (*p*-RIPK1, S166) and the downstream execution marker phosphorylated MLKL (*p*-MLKL). Western blot analysis reveals that MA@ULips treatment significantly reduces the levels of both *p*-RIPK1 (S166) and *p*-MLKL compared to the OGD/R group (Fig. S34b).

Necroptosis is typically triggered when the apoptotic pathway is inhibited. To provide more direct evidence supporting the induction of necroptosis, we pharmacologically blocked apoptosis using the caspase-8 inhibitor N-benzyloxycarbonyl-Ile-Glu (O-methyl)-Thr-Asp-[fmoc-Lys (Dabcyl)]-N-(4-methylcarbamate (Z-IETD-FMK). Upon treatment with Z-IETD-FMK, the expression of the necroptosis marker *p*-MLKL is significantly increased compared with the OGD/R group (Fig. S34c), indicating a shift of the cell death pathway toward necroptosis. Importantly, MA@ULips treatment significantly reduce the heightened *p*-MLKL levels even under OGD/R conditions potentiated by Z-IETD-FMK. These findings confirm the dynamic crosstalk between apoptotic and necroptotic pathways and demonstrate that MA@ULips can directly modulate downstream components of the necroptosis cascade to exert cytoprotective effects. Collectively, the results indicate that MA@ULips simultaneously suppress RIPK1 kinase activity and inhibit critical downstream effectors, supporting our hypothesis that MA@ULips target the RIPK1-PANoptosome complex and attenuate its pathological activity.

To further substantiate this functional dependency, PC12 cells were pretreated with Necrostatine-1s (Nec-1s), a selective RIPK1 inhibitor, followed by exposure to OGD/R conditions. The neuroprotective effect of MA@ULips was significantly diminished in the presence of RIPK1 inhibition, as evidenced by the absence of improvement in cell viability following treatment (Fig. S34d). These findings support the notion that MA@ULips can directly modulate the RIPK1-PANoptosome, thereby attenuating oxidative stress, suppressing neuroinflammation, and inhibiting the PANoptosis pathway. This mechanism underscores the potential of MA@ULips as a promising neuroprotective strategy for ischemic stroke therapy.

### 3.4 Microglia phenotype regulation and neuroinflammation reduction

**Fig. 4:**
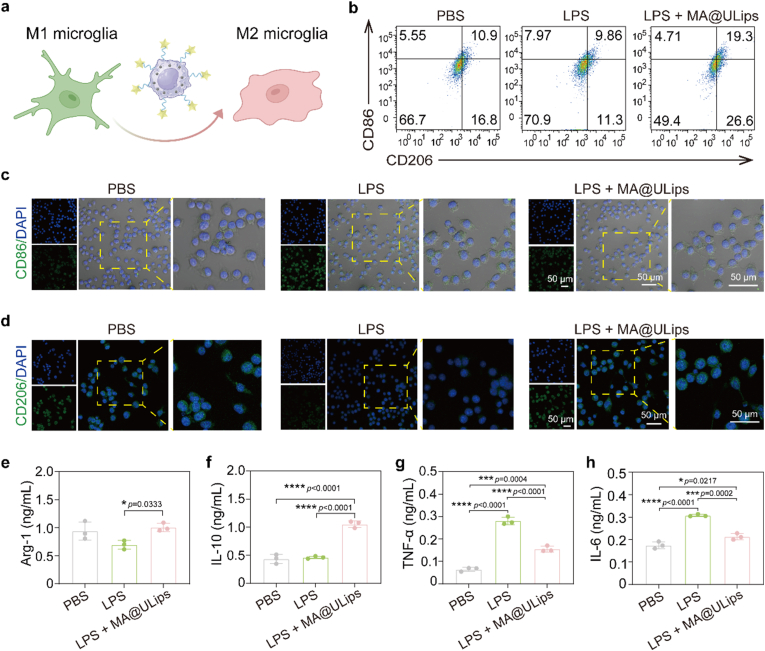
In vitro evaluation of microglia regulation of MA@ULips. (a) Scheme of microglia phenotype transition induced by MA@ULips. (b) Flow cytometry analysis of CD86 and CD206 in BV2 cells after diverse treatments. Representative immunofluorescence staining images of (c) CD86 and (d) CD206 in BV2 cells after various treatments. The level of (e) Arg-1, (f) IL-10, (g) TNF-α, and (h) IL-6 in the BV2 cells after different treatments (n = 3). Data are presented as mean ± SD. Statistical significance was calculated by one-way ANOVA with Tukey's multiple comparisons test. ∗*p* < 0.05, ∗∗∗*p* < 0.001, ∗∗∗∗*p* < 0.0001.

Microglia, the resident macrophages of the brain, play a crucial role in the response to ischemia injury, rapidly becoming activated and migrating toward the affected region following stroke. However, microglia exhibit a dual role in ischemic stroke, with their effects differing depending on the stage of the injury [53,54]. In the acute phase, microglia can exacerbate tissue damage through the release of pro-inflammatory cytokines, while in the recovery phase, they promote tissue repair and regeneration through anti-inflammatory responses [55]. Thus, modulating the phenotypic polarization of microglia may provide a strategy to alleviate ischemic injury and enhance recovery (Fig. 4a). To evaluate the potential of engineered macrophages for modulating microglial polarization, BV2 cells were exposed to LPS, a well-established inducer of inflammation. Flow cytometry was employed to assess the effects of MA@ULips on the phenotypic polarization of microglia by quantifying the proportions of M1 and M2 microglia (Fig. 4b). Treatment with MA@ULips leads to a remarkable increase in the ratio of M2-type microglia, with a nearly twofold increase in the proportion of CD206^+^ cells compared to the LPS group, whereas the proportion of M1-type microglia is significantly reduced, indicating MA@ULips treatment promotes the polarization of microglia from a pro-inflammatory M1 to a neuroprotective M2 phenotype, which is crucial for tissue repair following ischemic injury (Fig. S35).

Further investigation through immunofluorescence staining of pro-inflammatory marker CD86 and anti-inflammatory marker CD206 confirms these findings (Fig. 4c, d and Fig. S36a and b). In the LPS group, an increase in the green fluorescence intensity of CD86 and a decrease in CD206 fluorescence are observed, indicating a shift toward the pro-inflammatory M1 phenotype. Conversely, MA@ULips treatment leads to increased expression of CD206, suggesting that LPS induced inflammatory activation of microglia is effectively suppressed. This observation is consistent with the modulatory effects of MA@ULips on microglial polarization in the OGD/R-induced in vitro ischemia model (Fig. S36c and d). To further assess the anti-inflammatory effects of MA@ULips, ELISA was performed to measure the levels of key inflammatory factors. Pro-inflammatory cytokines, including TNF-α and IL-6, are significantly reduced in the MA@ULips group, while the levels of anti-inflammatory cytokines, such as IL-10 and Arg-1, are increased, indicating a shift toward a more favorable and reparative microglial phenotype (Fig. 4e–h). These findings suggest that MA@ULips not only facilitates the phenotypic transition of microglia from an ischemia-associated inflammatory state to a recovery-associated reparative state, but also enhances neuroprotection following ischemic reperfusion injury.

### 3.5 In vivo investigation of targeting ability, inflammation tracking and therapeutic effects of MA@ULips in ischemic stroke

**Fig. 5:**
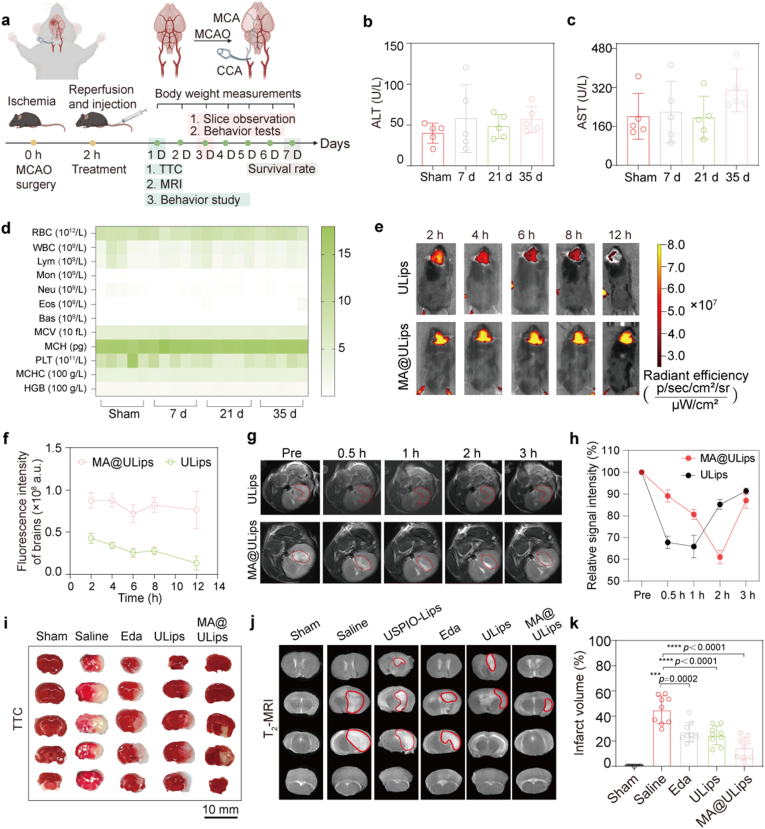
In vivo targeting and neuroprotection of MA@ULips. (a) Schematic representation of the MCAO/R model experiments. MCA: middle cerebral artery; CCA: common carotid artery. (b) Biochemical level of ALT at 7, 21 and 35 days post-injection (n = 5). (c) Biochemical level of AST at 7, 21 and 35 days post-injection (n = 5). (d) Hematological analysis (RBC, WBC, Lym, Mon, Neu, Eos, Bas, MCV, MCH, PLT, MCHC and HGB) (n = 5). (e) In vivo fluorescence imaging of Cy5.5-labeled ULips and MA@ULips in MCAO/R mice at 2, 4, 6, 8, and 12 h post-injection. (f) Quantitative analysis of brain fluorescence content of ULips and MA@ULips post-injection (n = 3). (g) T_2_-weighted MRI of MCAO/R mice before and after intravenous injection of ULips and MA@ULips. (h) Quantitative analysis of time dependence in T_2_-weighted signal intensities at ischemic site (n = 3). (i) Representative TTC staining images of brain slices after different treatments. (j) Representative T_2_-weighted MRI across different groups 24 h post-MCAO/R. (k) Quantification of infarct volume of brain slices treated with different formulations (n = 9). Note: Eda: Edaravone. Data are presented as mean ± SD. Significant differences were performed in (b), (c) and (l) using one-way ANOVA with Tukey's multiple comparisons test. Statistical significance in (f) was calculated via two-tailed Student's *t*-test. ∗∗∗*p* < 0.001, ∗∗∗∗*p* < 0.0001.

To assess the targeting ability, brain protection, and inflammation tracking of MA@ULips in the context of ischemia-reperfusion injury, a transient MCAO/R model was established (Fig. 5a). Briefly, MCAO/R was induced by inserting a filament into the middle cerebral artery for 60 min, followed by reperfusion. The mortality did not exceed 30 % in any group (Fig. S37). To evaluate the safety and blood compatibility of MA@ULips, routine blood tests and serum biochemical analyses were performed on days 7, 21, and 35 post-injection. Serum biochemical markers related to hepatic and renal functions, including aspartate aminotransferase (AST), alanine aminotransferase (ALT), and blood urea nitrogen (BUN), remain within normal ranges compared to the sham group, with only a slight increase in BUN observed on day 35 (Fig. 4b, c and Fig. S38). Hematological parameters, including red and white blood cell counts, hemoglobin, and platelet levels, also remain within physiological limits (Fig. 4d). Crucially, major immune cell populations, including leukocytes, monocytes, lymphocytes, neutrophils, eosinophils, and basophils, show no abnormal fluctuations, indicating minimal immunotoxicity. Furthermore, no significant hemolysis is detected in the MA@ULips-treated group (Fig. S39), confirming its excellent hemocompatibility. To further assess tissue-level biosafety, histological examination of major organs, including the heart, liver, spleen, lungs, spleen, lungs, kidneys, and brain, was conducted at multiple time points (days 7, 21 and 35 post-injection). H&E staining reveals no observable tissue damage inflammation, or morphological abnormalities in any of the examined organs (Fig. S40), suggesting the absence of systemic toxicity. Collectively, these data confirm that MA@ULips exhibit negligible immunotoxicity, excellent biocompatibility, and favorable biosafety profiles, suggesting its suitability for further development in the treatment of ischemic stroke.

Prior to evaluating the therapeutic effects of MA@ULips, we first investigated the in vivo stability of MA@ULips. Frankly speaking, direct detection of free TEMPO released from its amide-linked conjugate in circulation is hindered by the lack of sensitive and specific analytic probes. Cy5.5-NH_2_, a fluorescent analogue, was employed as a surrogate for TEMPO-NH_2_. A lipid conjugate, DOPE-PEG_2000_-Cy5.5, was synthesized via EDC/NHS-mediated amidation reaction between DOPE-PEG_2000_-COOH and Cy5.5-NH_2_. To assess in vivo release, free Cy5.5 levels in plasma samples following ultrafiltration were monitored after intravenous injection. Free Cy5.5 is not detected at 1, 4, 8, and 12 h post-injection (Fig. S41), suggesting that the amide linkage is robust and that DOPE-PEG_2000_-TEMPO exhibits high in vivo stability. This enhanced stability is expected to facilitate brain accumulation and contribute to the therapeutic efficacy of the nanoplatform. In vivo fluorescence imaging of ischemic brains at various time points post-injection demonstrates that the fluorescence signal of MA@ULips remains markedly stronger at 12 h compared to that of ULips, which shows comparatively weaker fluorescence. This enhanced signal can be attributed to the intrinsic targeting capabilities conferred by the macrophage membrane coating of MA@ULips (Fig. 5e and f), indicating superior accumulation at the site of cerebral ischemia. The brain-targeting efficiency of MA@ULips was assessed by evaluating their distribution across major organs, including the brain, heart, liver, spleen, lung, and kidneys. Fluorescence analysis reveals that MA@ULips achieved brain-targeting efficiencies of 40.5 % and 37.9 % at 6 h and 12 h post-intravenous administration, respectively (Fig. S42), while exhibiting reduced accumulation in non-target tissues. Notably, hepatic and splenic uptake was observed, consistent with reticuloendothelial system (RES) clearance, whereas lower fluorescence signals were detected in the heart, lungs, and kidneys. These results collectively indicate that MA@ULips demonstrate sustained and selective brain-targeting capability.

To further validate the brain-targeting ability of MA@ULips under inflammatory conditions, T_2_-weighted MRI was employed to evaluate their biodistribution and homing behavior in vivo. At 2 h postinjection, mice treated with MA@ULips exhibit pronounced contrast enhancement in the ischemic region, with a well-defined lesion boundary, in contrast to the partial signal recovery observed in the ULips group (Fig. 5g). This distinct enhancement is likely due to the prolonged circulation time and active targeting ability of the macrophage-derived delivery system. Furthermore, quantitative MRI analysis reveals that the relative signal intensity of MA@ULips in the ischemic region decreased by over 40 % within 2 h, reflecting efficient cellular homing and retention within inflamed brain tissue (Fig. 5h). Inductively coupled plasma mass spectrometry (ICP-MS) analysis demonstrates that the ischemic hemisphere of MA@ULips-treated mice exhibits a marked increase in iron content (668 μg/g) compared with the sham group (599 μg/g) (Fig. S43), consistent with the in vivo T_2_-weighted MRI findings. This provides quantitative chemical evidence supporting the MRI T_2_ signal changes, confirming that the observed signal alteration originates from exogenous iron delivery rather than endogenous sources.

Subsequently, the therapeutic efficacy of MA@ULips was evaluated by measuring infarct volume via TTC staining at 24 h post-reperfusion, with an injection dose of 1 × 10^6^ cells/100 μL and 3 mg/kg ULips (Fig. 5i). Significant cerebral infarction is observed in untreated MCAO/R mice, while MA@ULips treatment markedly reduces the infarct volume from approximately 44.8 %–14.5 % (Fig. 5k). To further dissect the contribution of each component, we introduced two additional groups, including the RAW 264.7 cells alone and a non-fused physical mixture of RAW 264.7 cells and ULips, quantified cerebral infarction by TTC staining (Fig. S44a). MA@ULips produce a pronounced reduction in infarct size, decreasing mean infarct area from 33.8 % in untreated MCAO/R mice to 11.8 %. By contrast, the Eda, ULips, MA, and MA + ULips groups exhibit mean infarct areas of 20.0 %, 20.9 %, 21.7 %, and 19.6 %, respectively (Fig. S44b). The superior neuroprotection afforded by MA@ULips is most plausibly explained by synergistic integration of the two functional components. ULips provide potent reactive-oxygen-species scavenging that mitigates oxidative injury, while macrophage-mediated inflammatory homing concentrates the therapeutic payload within the ischemic territory. Membrane-fusion functionalization yields a hybrid, cell nanocarrier construct that preserves macrophage biological activity while delivering the antioxidative capacity of the liposomal cargo, thereby producing greater efficacy than either single component or their simple admixture.

Ischemic stroke induced vascular occlusion and BBB disruption typically produce vasogenic cerebral edema, which appears as hyperintense regions on T_2_-weighted magnetic resonance imaging (T_2_WI). As shown in Fig. 5j, T_2_WI at 24 h post-reperfusion revealed extensive hyperintensity in saline-treated animals, occupying approximately 35.0 % of the ipsilateral (ischemic) hemisphere. Importantly, mice treated with USPIO-loaded blank liposomes (USPIO-Lips) exhibit comparable T_2_ hyperintensity, indicating that the intrinsic susceptibility of USPIO does not mask or materially confound detection of edema or compromise the evaluation of therapeutic efficacy. By contrast, treatments with Eda, ULips, and MA@ULips produce substantial reductions in T_2_ hyperintense volume. MA@ULips reduce edema area of 11.2 % of the ipsilateral hemisphere (Fig. S45), demonstrating the superior anti-edema efficacy of the macrophage-engineered nanoplatform. Furthermore, neurological deficit scores are significantly improved in the MA@ULips group compared to controls, indicating enhanced functional recovery (Fig. S46).

**Fig. 6:**
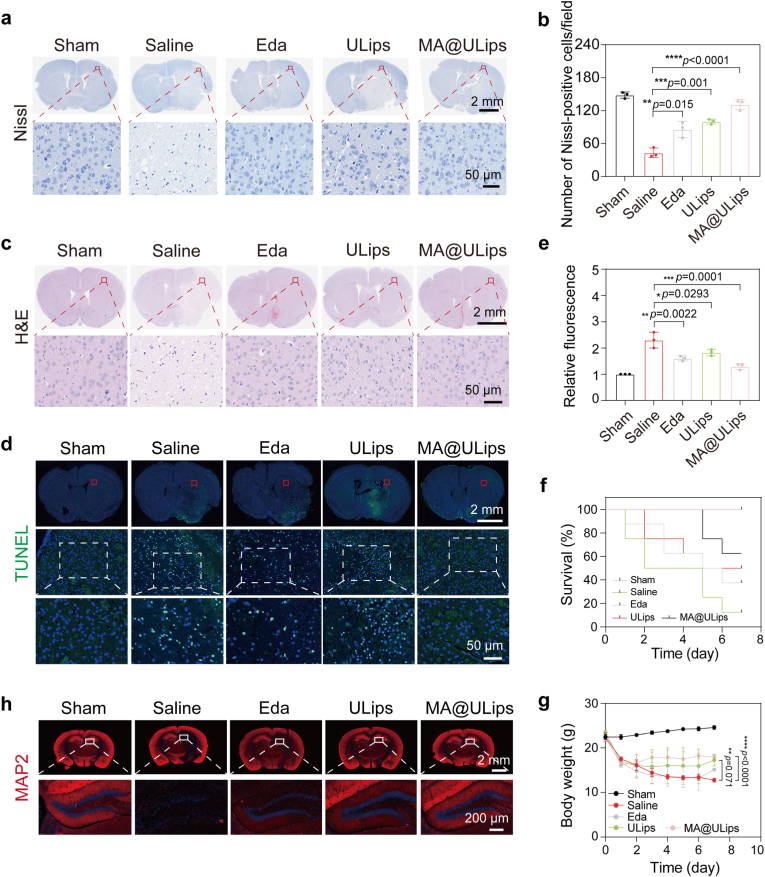
Therapeutic validation of MA@ULips for neuronal survival and antiapoptotic effects. (a) Representative images and (b) quantitative analysis of Nissl staining performed 3 days post- MCAO/R (n = 3). (c) Representative H&E-stained images of pathological brain tissues in MCAO/R mice after different treatments. (d) Immunofluorescence staining and (e) quantitative analysis for TUNEL in ischemic brain regions after various treatments (n = 3). (f) Survival curves of MCAO/R mice receiving different treatments (n = 8). (g) Body weight of MCAO/R mice receiving different treatments (n = 3). (h) Representative immunofluorescence images of MAP2 staining after different treatments. Data are presented as mean ± SD. Statistical significance was calculated by one-way ANOVA with Tukey's multiple comparisons test. ∗*p* < 0.05, ∗∗*p* < 0.01, ∗∗∗*p* < 0.001, ∗∗∗∗*p* < 0.0001.

To explore the neuroprotective effects of MA@ULips, brain tissue damage, neuronal necrosis, and apoptosis were assessed on day 3 after MCAO/R. Nissl staining reveals significant neuronal injury in the MCAO/R group compared to the sham group, with positive cell counts of 43.0 in the sham group and 148.3 in the MCAO/R group, respectively (Fig. 6a and b). In contrast, MA@ULips treatment significantly improves neuronal survival, with a positive cell count of 131.0. Additionally, H&E staining demonstrates that MA@ULips treatment alleviates the extent of necrosis and vacuolization in the brain tissues, further supporting its neuroprotective effect (Fig. 6c). To assess apoptosis, TUNEL staining was performed. The MCAO/R group shows abundant apoptotic cells, as evidence by extensive green fluorescence, while MA@ULips group exhibits a marked reduction in positive cells, indicating effective suppression of apoptosis (Fig. 6d and e).

Moreover, immunofluorescence staining of the neuron marker-NeuN shows that MA@ULips treatment results in a substantial increase in NeuN-positive cells, suggesting that MA@ULips effectively mitigates neuronal loss following ischemic stroke (Fig. S47). The survival rate of MCAO/R mice is significantly improved following MA@ULips treatment, reaching 62.5 %, in comparison to 12.5 %, 37.5 %, and 50.0 % for the saline, Eda, and ULips groups, respectively, by day 7 post-stroke (Fig. 6f). Body weight analysis further corroborates the neuroprotective effects, as mice treated with MA@ULips exhibit weight recovery post-stroke, whereas the saline group experiences significant weight loss (Fig. 6g). In addition, MAP-2 staining on day 7 reveals that neurons in the cortical region of the MA@ULips treated group recovers significantly, with those in the hippocampus show less recovery (Fig. 6h). This suggests that while cortical neurons benefit from MA@ULips treatment, hippocampal recovery, associated with higher cognitive functions such as learning and memory, is more challenging, highlighting the complexity of full functional recovery post-stroke.

### 3.6 Enhancing neurological recovery and modulating ischemic inflammatory microenvironment

**Fig. 7:**
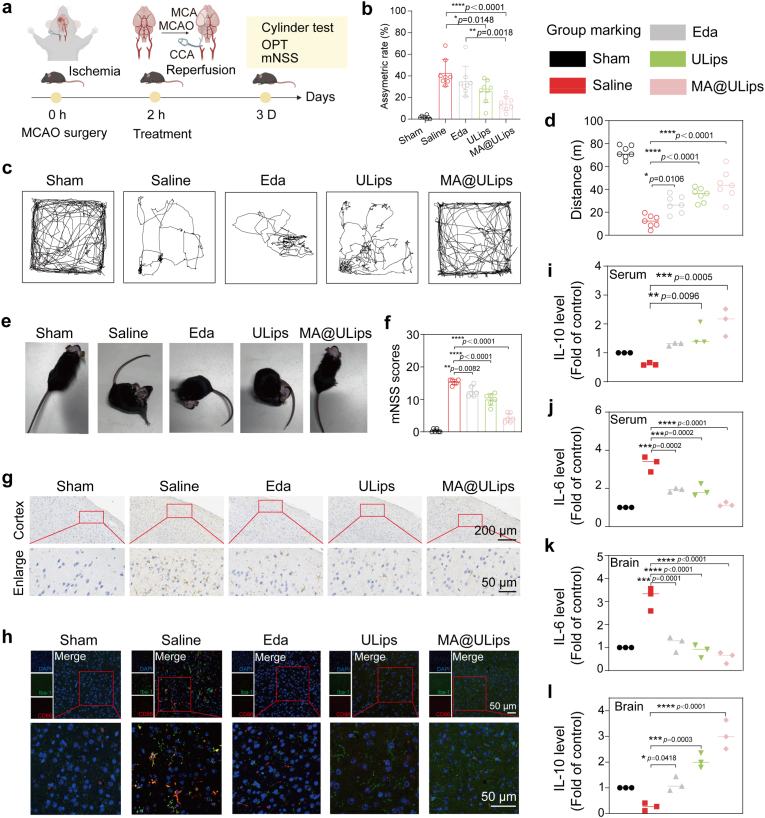
MA@ULips enhancing neurological functional recovery and modulating ischemic inflammatory microenvironment. (a) Experimental design involved multiple functional tests to evaluate the neurological outcomes of MA@ULips. (b) Forelimb asymmetry rate assessed through the cylinder test (n = 8). (c) Motion paths in the open field test. (d) Quantitative analysis of movement distance (n = 7). (e) Representative images of sham-operated mice and MCAO/R mice after different treatments. (f) Neurological deficit evaluation by the mNSS (n = 6). (g) Immunohistochemical staining for GFAP on brain tissue from the ischemic cortex area after different treatments. (h) Immunofluorescent staining of brain tissue for Iba-1 and CD86 after different treatments. (i–j) The levels of (i) IL-10 and (j) IL-6 in serum of ischemic mice after different treatments (n = 3). (k–l) The levels of (k) IL-6 and (l) IL-10 in brain tissues of ischemic mice after different treatments (n = 3). Data are presented as mean ± SD. Statistical significance was calculated by one-way ANOVA with Tukey's multiple comparisons test. ∗*p* < 0.05, ∗∗*p* < 0.01, ∗∗∗*p* < 0.001, ∗∗∗∗*p* < 0.0001.

Behavioral research using animal models plays a crucial role in evaluating the therapeutic effect and security, providing valuable insights into their potential clinical applications. We employed a combination of cylinder test, mNSS, and OPT to assess movement, sensory, and reflex functions following MCAO/R (Fig. 7a). In the cylinder test, normal mice typically take contact with the walls of the cylinder using both forelimbs, while ischemia-induced MCAO/R mice predominantly rely on their unaffected limbs, demonstrating a characteristic asymmetry in limb use (Fig. 7b). Mice treated with MA@ULips exhibit a significantly lower asymmetry rate compared to the MCAO/R group, suggesting improved limb function and recovery from ischemic injury, indicating that MA@ULips enhance motor coordination and sensory recovery after stroke. The OPT was used to further evaluate motor function, in which the sham group mice exhibit significantly longer walking distances compared to MCAO/R and treatment groups. Both ULips and MA@ULips-treated mice demonstrate a marked improvement in locomotor activity, walking significantly longer distances than MCAO/R group, highlighting the potential of MA@ULips in promoting motor recovery post-stroke (Fig. 7c and d).

Notably, ischemic mice typically display hemiplegic behavior, characterized by an inability to walk in a straight line or extend their tails (Fig. 7e). In contrast, the MA@ULips group exhibits improved motor coordination and reduced hemiplegic behavior, outperforming the MCAO/R group. In addition, the mNSS was employed to evaluate the overall neurological dysfunction in the mice (Fig. 7f). The results show that MA@ULips treatment significantly improves the neurological function of MCAO/R mice, with scores reflecting enhanced motor coordination, sensory integration, and reflex activity compared to untreated MCAO/R animals, underscoring the therapeutic potential of MA@ULips in mitigating the neurological deficits induced by ischemic stroke.

Following brain ischemia, microglia and astrocytes undergo rapidly activated, characterized by distinct morphological transformations in which microglia adopt an amoeboid shape, whereas astrocytes exhibit pronounced hypertrophy of the cell body. These structural changes reflect shifts in their functional states, corresponding to pro-inflammatory polarization and glial scar formation, respectively. To elucidate the mechanisms underlying the therapeutic effects of MA@ULips, we assessed neuroinflammation, a central pathological component of ischemic brain injury, by assessing glial activation. Immunohistochemical staining for GFAP, a canonical marker of reactive astrocytes (Fig. 7g), demonstrated a significant reduction in GFAP-positive cells in the cortical region of MA@ULips-treated MCAO/R mice compared with untreated controls. Similarly, microglial activation was assessed using Iba-1, a widely recognized pan-microglial marker (Fig. 7h). Immunostaining reveals a notable decrease in CD86 positive microglia in the MA@ULips group relative to the other groups, indicating attenuation of the pro-inflammatory M1-like phenotype. In addition, overall Iba-1 expression was substantially reduced in MA@ULips-treated mice, further supporting the suppression of microglial overactivation. These findings indicate that MA@ULips not only reverse pathological morphological changes in glial cells but also modulate their activation states, ultimately fostering a neuroprotective microenvironment conducive to central nervous system repair.

To quantify the effects of MA@ULips on inflammation, serum and brain tissue levels of key cytokines were measured. It has been found that MA@ULips treatment significantly reduces the levels of pro-inflammatory cytokines, such as IL-6, while promoting the expression of anti-inflammatory cytokines, such as IL-10, both in the serum and in brain tissues (Fig. 7i–l). In conclusion, these findings highlight the dual role of MA@ULips in both regulating glial cell functional states and reducing systemic and local neuroinflammation, thereby contributing to enhanced neurological recovery.

### 3.7 Therapeutic mechanism of MA@ULips on ischemic stroke

**Fig. 8:**
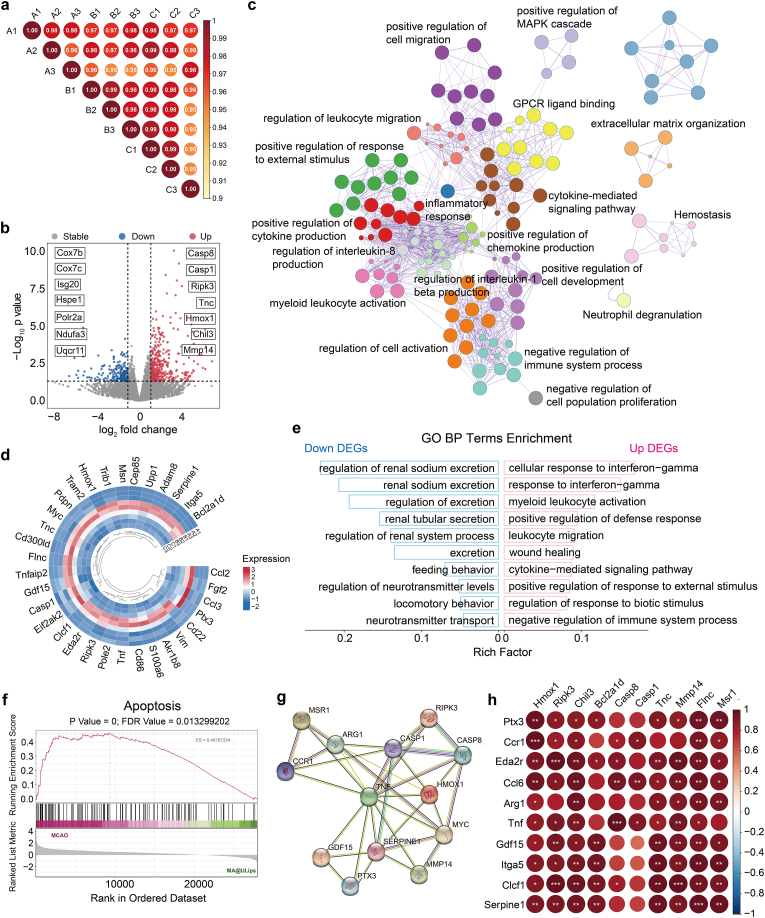
RNA-seq analysis of MA@ULips alleviating ischemia-induced neuroinflammation. (a) Correlation analysis of different groups. (A1-A3, control; B1–B3, MCAO/R; C1–C3, MA@ULips). (b) Volcano plot presenting the differentially expressed genes between MCAO/R and MA@ULips groups. (c) Gene enrichment pathway analysis through GO function analysis of differentially expressed genes. (d) Heatmap from RNA-seq analysis displaying the expression patterns of PANoptosis-related genes across different groups. (e) GO BP enrichment analysis of differentially expressed genes between MCAO/R and MA@ULips groups. (f) GSEA of apoptosis signaling pathway. (g) Protein and protein interaction network diagram including ROS and PANoptosis-related proteins. (h) Gene correlation analysis between MCAO/R and MA@ULips groups comprising ROS-related differentially expressed genes and PANoptosis-related differentially expressed genes.

To further elucidate the therapeutic mechanism underlying the effects of MA@ULips in ischemic stroke, RNA sequencing (RNA-seq) on brain tissues from mice was performed. Correlation coefficient analysis of the RNA-seq data reveals high correlation between the control and MA@ULips groups, preliminarily indicating negligible differences in gene expression between the control and MA@ULips groups (Fig. 8a). This analysis shows a significant upregulation of genes associated with RIPK1-PANoptosomes including RIPK1, NLRP3, and CASP8 in the MCAO/R group compared to the control group, underscoring the inflammatory response triggered by ischemic injury (Fig. S48). Further analysis of differential gene expression between the MCAO/R and MA@ULips-treated groups exhibits that 591 genes show altered expression following treatment, with 378 genes upregulated and 213 genes downregulated. Notably, the expression of RIPK1-PANoptosomes-related genes, such as RIPK3, CASP3, and CASP8, is significantly reduced in the MA@ULips-treated group, in contrast to the MCAO/R group (Fig. 8b). Gene enrichment pathway analysis through gene ontology (GO) function analysis of differentially expressed genes (DEGs) demonstrates that MCAO/R injury primarily promoted the activation of inflammatory response, especially interleukin-1 beta production and cytokine-mediated signaling pathway (Fig. 8c).

Additionally, a heatmap of 33 genes closely associated with PANoptosis is generated to further investigate the effects of MA@ULips on gene expression. The results from the heatmap are consistent with those observed in the volcano plot, confirming the therapeutic effects of MA@ULips in regulating PANoptosis-related genes (Fig. 8d). Similarly, GO biological process (BP) enrichment analysis demonstrates that MA@ULips treatment significantly downregulates processes such as leukocyte migration, cytokine-mediated signaling pathway and regulation of response to external stimulus, which are all central to the inflammatory response (Fig. 8e). Gene set enrichment analysis (GSEA) reveals significant enrichment of pathways related to apoptosis, necroptosis, and PI3K-Akt signaling in the MCAO/R group, all of which are normalized to baseline levels following MA@ULips treatment (Fig. 8f and S49).

Functional pathway enrichment analysis reveals that multiple key inflammatory and cell death-related pathways were significantly modulated following MA@ULips treatment. These include the TNF signaling pathway, apoptosis, necroptosis, Toll-like receptor signaling, and NF-κB signaling pathways. Notably, these pathways were markedly downregulated in the MA@ULips-treated group compared to the MCAO/R group, suggesting that MA@ULips exert therapeutic effects through coordinated suppression of pro-inflammatory and programmed cell death signaling cascades (Fig. S50). Moreover, the protein–protein interaction network of DEGs suggested that ischemic stroke mainly activated PANoptosis-associated functional pathways (Fig. 8g). In addition, Kyoto encyclopedia of genes and genomes (KEGG) pathway analysis displays activation of the apoptosis, necroptosis, NF-κB signaling, NOD-like receptor, and Toll-like receptor pathways following MCAO/R, with significant upregulation of inflammatory responses, including pyroptosis (Fig. S51). Among these pathways, NF-κB indirectly promotes pyroptosis by upregulating NLRP3 inflammasome assembly, while NOD-like receptor function as direct executors of pyroptosis.

Furthermore, the release of damage-associated molecular patterns (DAMPs) during cellular injury activates toll-like receptors (TLRs), which in turn enhance NF-κB signaling, thereby reinforcing the expression of NLRP3 and pro-inflammatory cytokines. Importantly, MA@ULips treatment leads to the downregulation of these pro-inflammatory pathways. Gene correlation analysis between MCAO/R and MA@ULips groups comprising ROS-related DEGs (e.g., Eda2r, Ccl6, Arg 1, Tnf, and Gdf15) and PANoptosis-related DEGs (e.g., Ripk3, Casp8, Casp 1, Tnc, and Mmp 14) further validates the involvement of PANoptosis (Fig. 8h). Furthermore, immunohistochemical staining for caspase-1 was performed on brain tissue sections from the cortex and hippocampus, which demonstrates a significant reduction in caspase-1 expression in the MA@ULips-treated group compared to MCAO/R controls, supporting the role of MA@ULips in inhibiting PANoptosis-related cell death and neuroinflammation (Fig. S52). These results provide compelling evidence that MA@ULips effectively relieves neuroinflammation and mitigates neuronal injury in ischemic stroke by inhibiting RIPK1-PANoptosomes and reducing ROS levels.

## 4 Conclusions

In summary, we present an MRI-trackable engineered macrophage biohybrid nanoplatform, designed to regulate multiple cell death pathways following ischemia-reperfusion injury. This advanced system leverages the combined advantages of precise inflammation targeting, ROS scavenging, PANoptosis inhibition, and inflammation visualization to effectively counteract the complex and dynamic pathological changes associated with reperfusion-induced neuroinflammation. These inflammatory processes often drive neural cells toward programmed or unprogrammed cell death pathways, including apoptosis, necrosis, and pyroptosis. By simultaneously regulating these diverse cell death pathways, MA@ULips promote enhanced neurofunctional recovery. Furthermore, MA@ULips demonstrate excellent biocompatibility, underscoring their potential for safe and effective application in clinical settings. As a multi-pathway cell death suppression strategy, it offers a promising approach for addressing the intricate neuroinflammatory milieu following stroke. Additionally, this work provides valuable insights for the development of future therapeutic interventions aimed at managing neuroinflammation in other central nervous system disorders, paving the way for broader applications in neuroprotective treatments.

## CRediT authorship contribution statement

**Wenhui Jiang:** Conceptualization, Investigation, Methodology, Writing – original draft. **Chundongqiu Xia:** Investigation, Methodology. **Zhimeng Cui:** Investigation, Methodology. **Lanhao Shi:** Formal analysis, Methodology. **Wei Feng:** Conceptualization, Resources, Writing – review & editing. **Yu Chen:** Funding acquisition, Methodology, Resources, Writing – review & editing. **Jun Zhang:** Funding acquisition, Writing – review & editing.

## Declaration of competing interest

The authors declare no interest conflict. They have no known competing financial interests or personal relationships that could have appeared to influence the work reported in this paper.

## Data Availability

Data will be made available on request.
