# Supplementary material for: Redox-modulating macrophage biohybrid nanoplatform for targeted RIPK1-PANoptosome suppression in ischemic stroke
Source: Redox Biol. 2025 Dec 24;89:103997. doi: 10.1016/j.redox.2025.103997 (PMC12808503; doi:10.1016/j.redox.2025.103997)
Supplement: Multimedia component 1 [file mmc1.docx]

**Supporting Information**

**Redox-Modulating Macrophage Biohybrid Nanoplatform for Targeted RIPK1-PANoptosome Suppression in Ischemic Stroke**

Wenhui Jiang ^a, 1^, Chundongqiu Xia ^a, 1^, Zhimeng Cui ^a, 1^, Lanhao Shi ^b^, Wei Feng ^b, *^, Yu Chen ^b, *^, and Jun Zhang ^a, *^

^a^ *Department of Radiology, Huadong Hospital, Shanghai Key Laboratory of Clinical Geriatric Medicine, Shanghai Institute of Geriatrics and Gerontology, State Key Laboratory of Brain Function and Disorders, Fudan University, Shanghai, 200040, P. R. China.*

^b^ *Materdicine Lab, School of Life Sciences, Shanghai University, Shanghai, 200444, P. R. China.*

^*^ Corresponding authors.

E-mail: fengw@shu.edu.cn (W. Feng); chenyuedu@shu.edu.cn (Y. Chen);

zhangjun_zj@fudan.edu.cn (J. Zhang)

^1^ These authors contributed equally to this work.


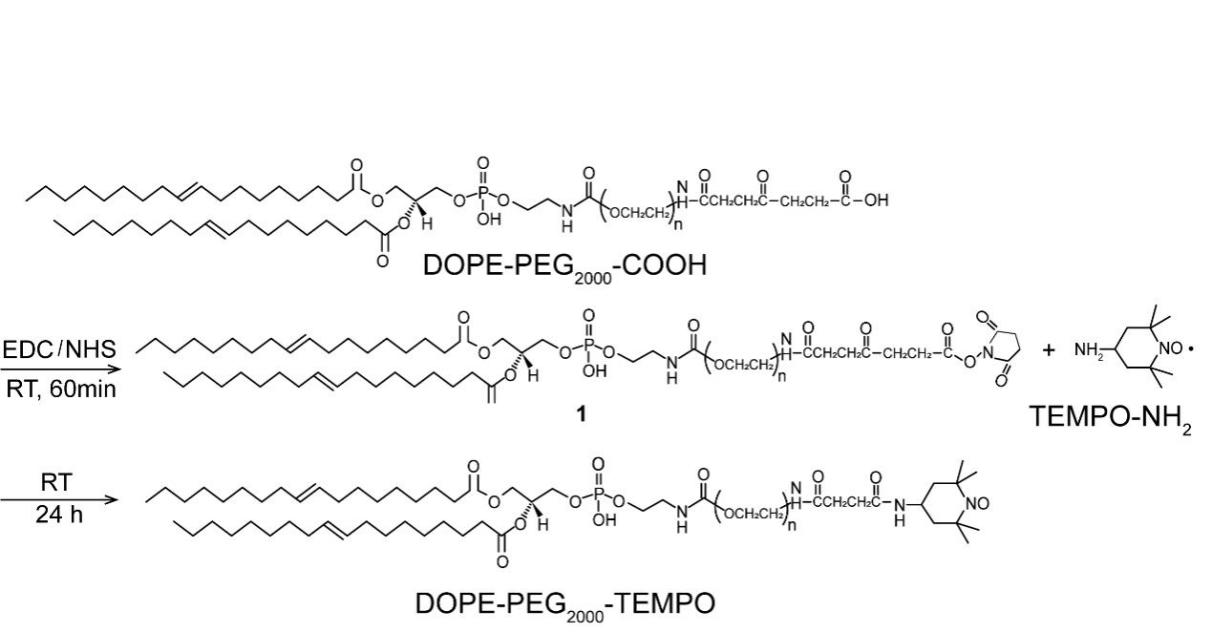


**Fig. S1. Synthetic route of DOPE-PEG_2000_-TEMPO.** The compound 1 was synthesized using DOPE-PEG_2000_-COOH, EDC and NHS as source materials. The DOPE-PEG_2000_-TEMPO was obtained by reacting TEMPO-NH_2_ with the compound 1.


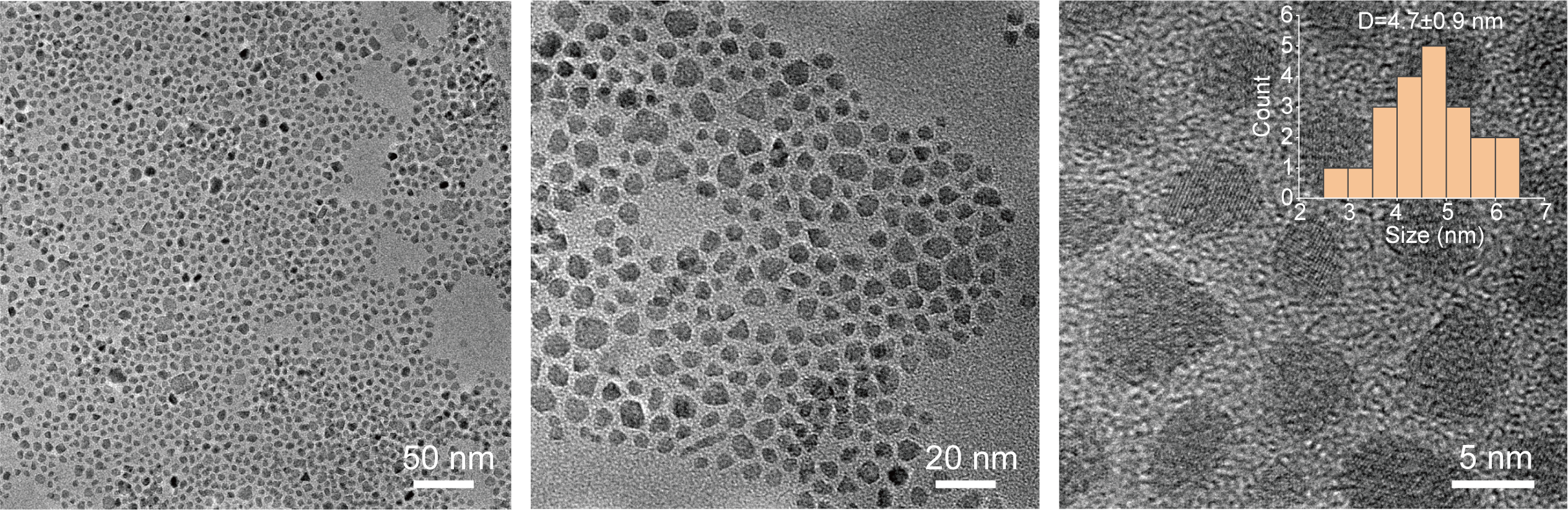


**Fig. S2**. TEM images of USPIO nanoparticles.

**
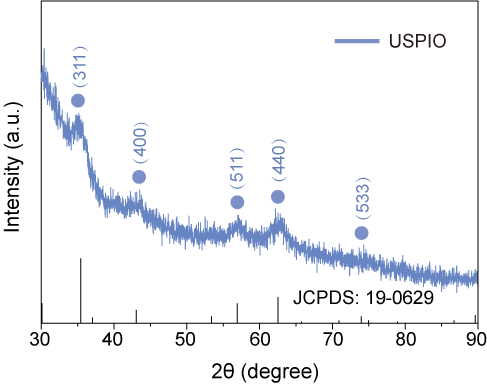
**

**Fig. S3.** XRD pattern of USPIO nanoparticles.


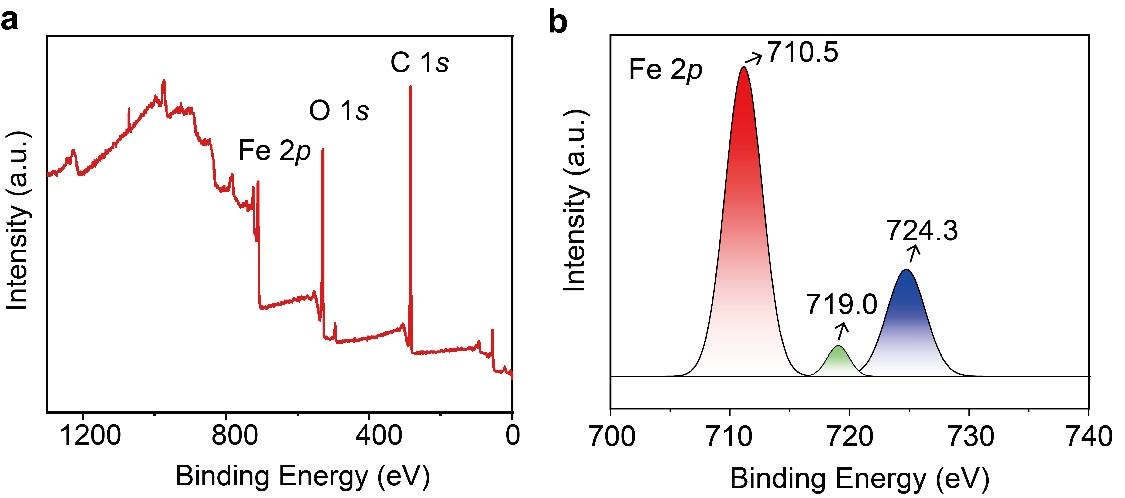


**Fig. S4. XPS analysis results of USPIO.** (a) Full XPS spectrum of USPIO. (b) XPS spectrum of Fe 2p.


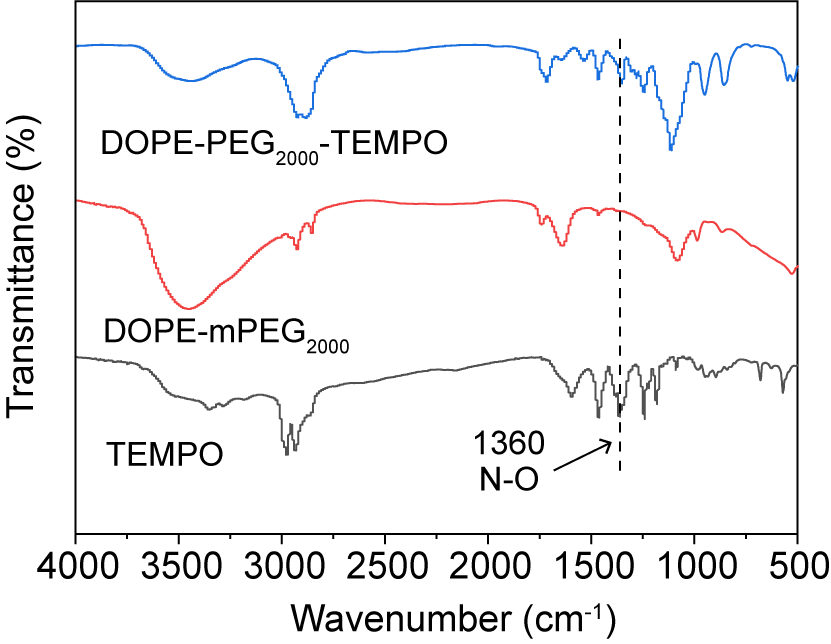


**Fig. S5.** FT-IR spectra of TEMPO, DOPE-mPEG_2000_, and DOPE-PEG_2000_-TEMPO.


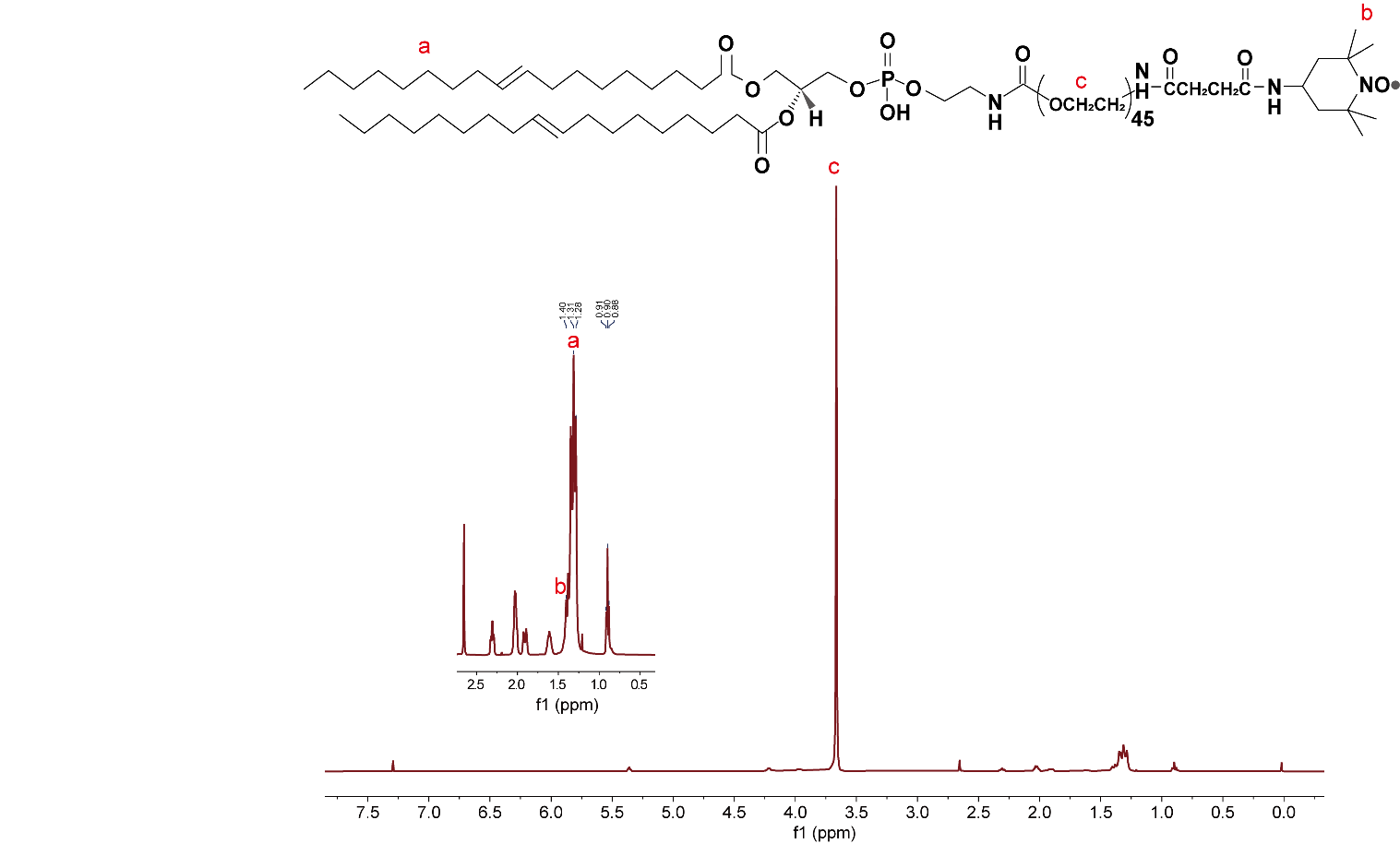


**Fig. S6. ^1^H NMR spectrum of the DOPE-PEG_2000-_TEMPO phospholipid.** The ^1^H NMR spectrum was recorded at 400 MHz using CDCl_3_ as a solvent.


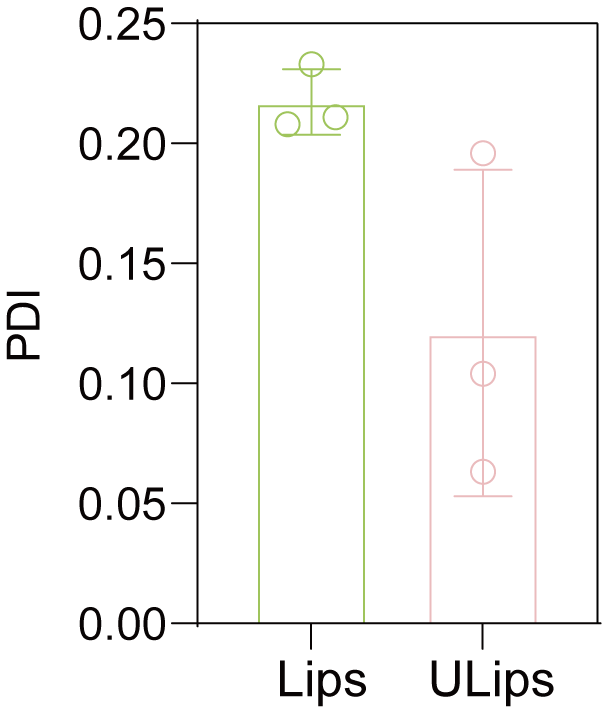


**Fig. S7.** PDI profiles of Lips and ULips.


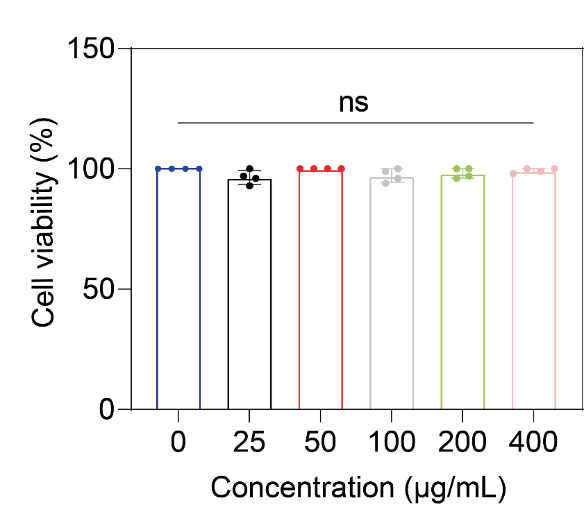


**Fig. S8. Cytotoxicity study of ULips to RAW 264.7 cells in vitro.** Quantification of the cell viability of RAW 264.7 cells after treatment with ULips at different concentrations (n = 4). Data are presented as mean ± standard deviation (SD). Statistical significance was calculated by one-way ANOVA with Tukey's multiple comparisons test. ns, no significance.


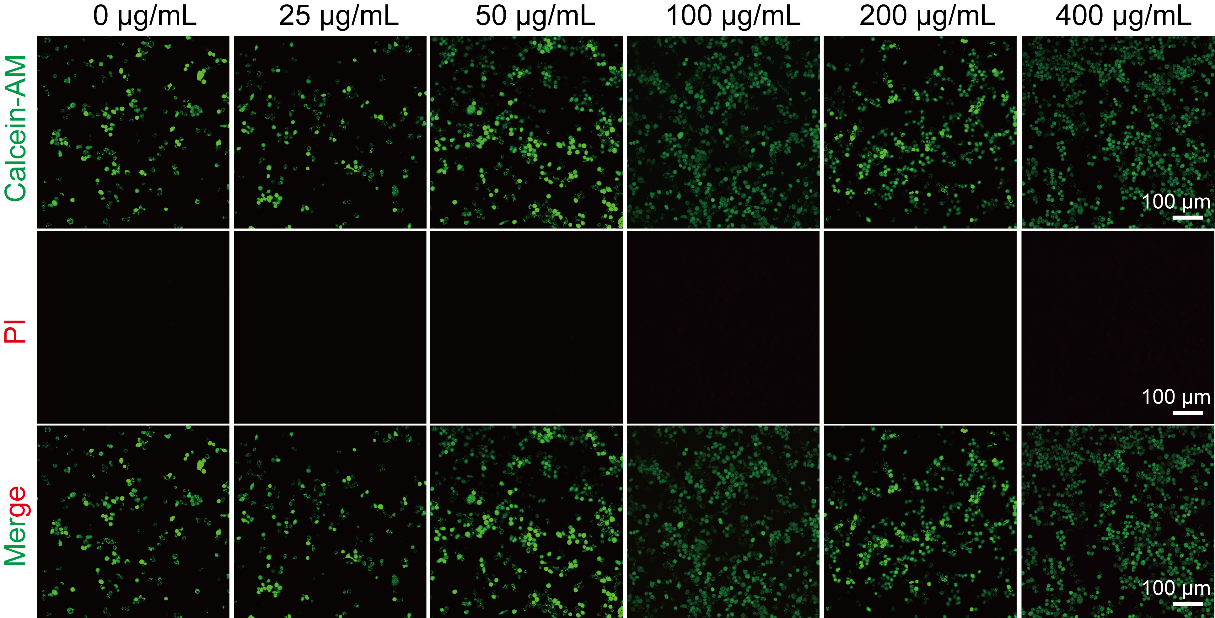


**Fig. S9.** Confocal microscope images of live/dead staining of RAW 264.7 cells after treatment with ULips at different concentrations.


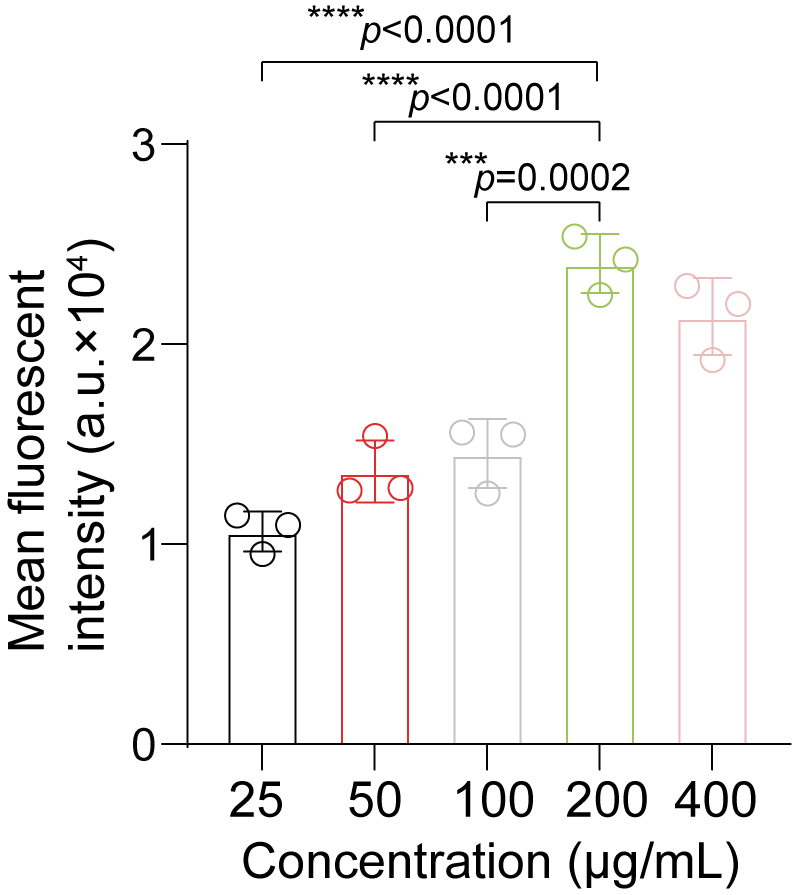


**Fig. S10.** Mean fluorescent intensity of macrophages after incubation with different concentrations of ULips-DiO (25, 50, 100, 200 and 400 μg/mL) analysed by flow cytometry (n = 3). Data are presented as mean ± SD. Statistical significance was calculated by one-way ANOVA with Tukey's multiple comparisons test. ****p* < 0.001, *****p* < 0.0001.


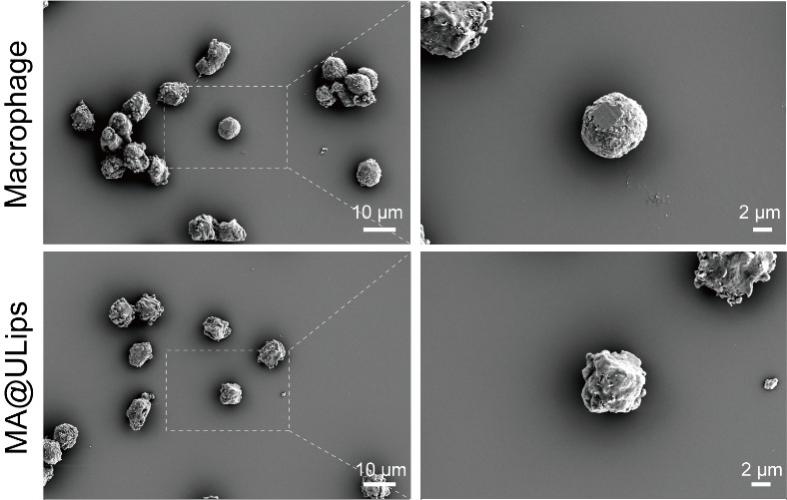


**Fig. S11.** SEM images of macrophages and MA@ULips.


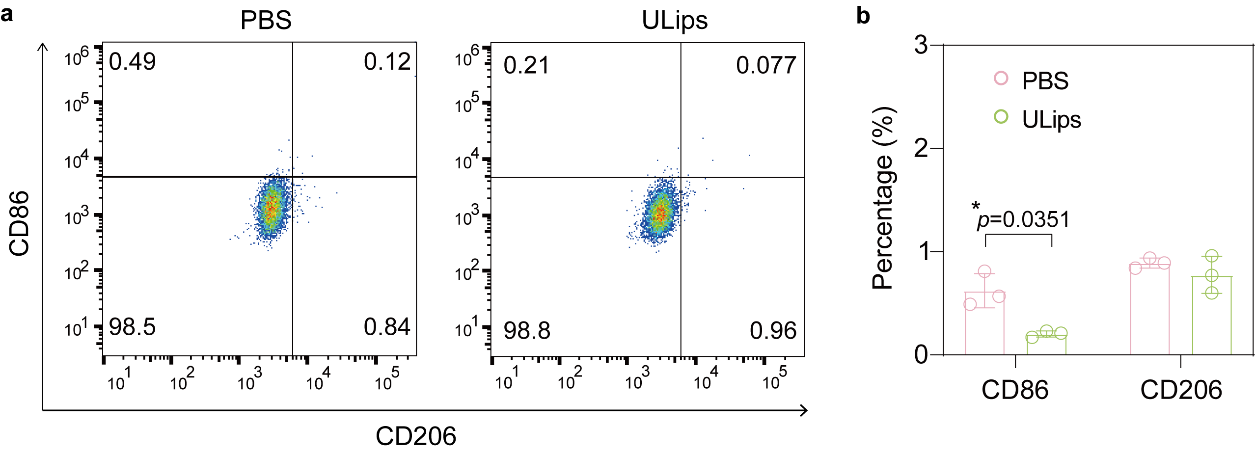


**Fig. S12.** (a) Flow cytometry and (b) corresponding quantification analysis of CD86 and CD206 in macrophages after incubation with ULips (n = 3). Data are presented as mean ± SD. Statistical significance was calculated by one-way ANOVA with Tukey's multiple comparisons test. **p* < 0.05.


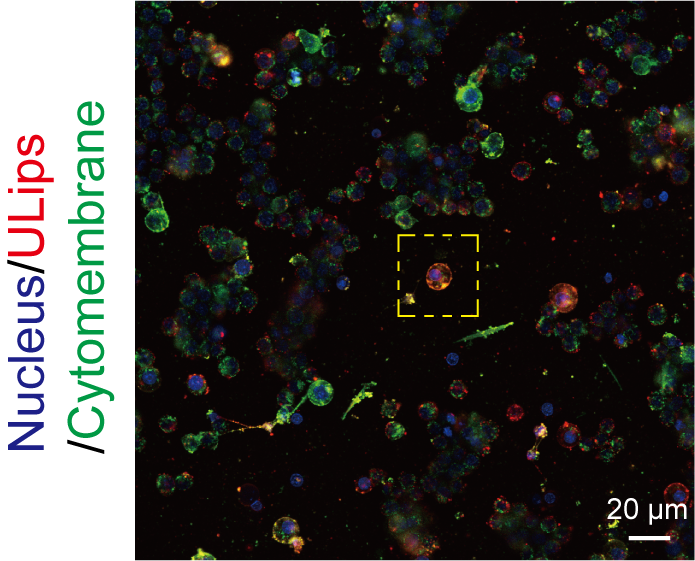


**Fig. S13.** CLSM images of DiO labeled macrophages after incubation with Dil labeled ULips.


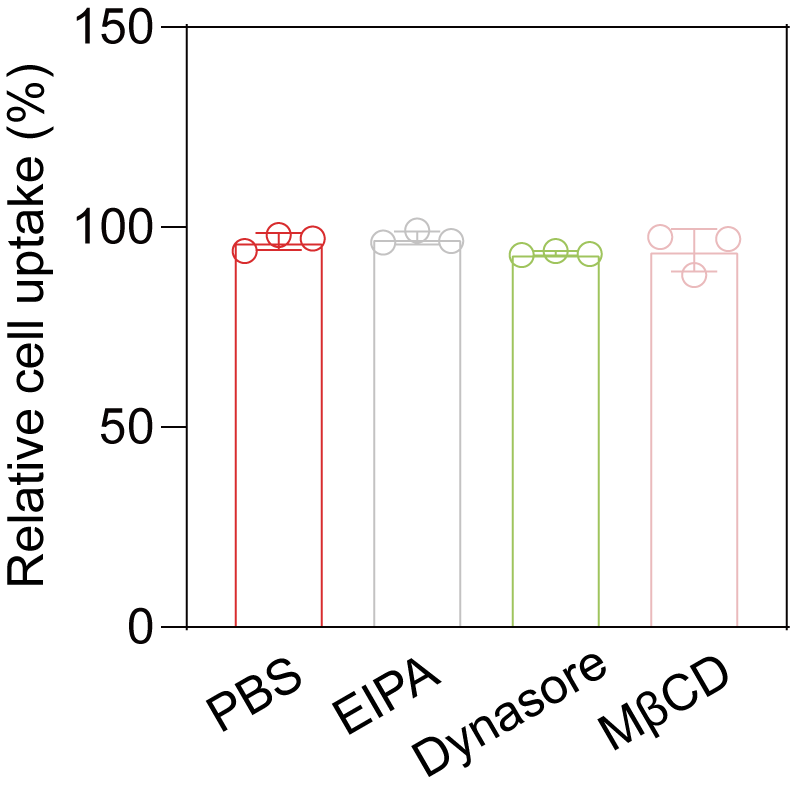


**Fig. S14.** Flow cytometry analysis of ULips uptake by RAW 264.7 cells after treatment with various inhibitors. (n = 3). Data are presented as mean ± SD. Statistical significance was calculated by one-way ANOVA with Tukey's multiple comparisons test.


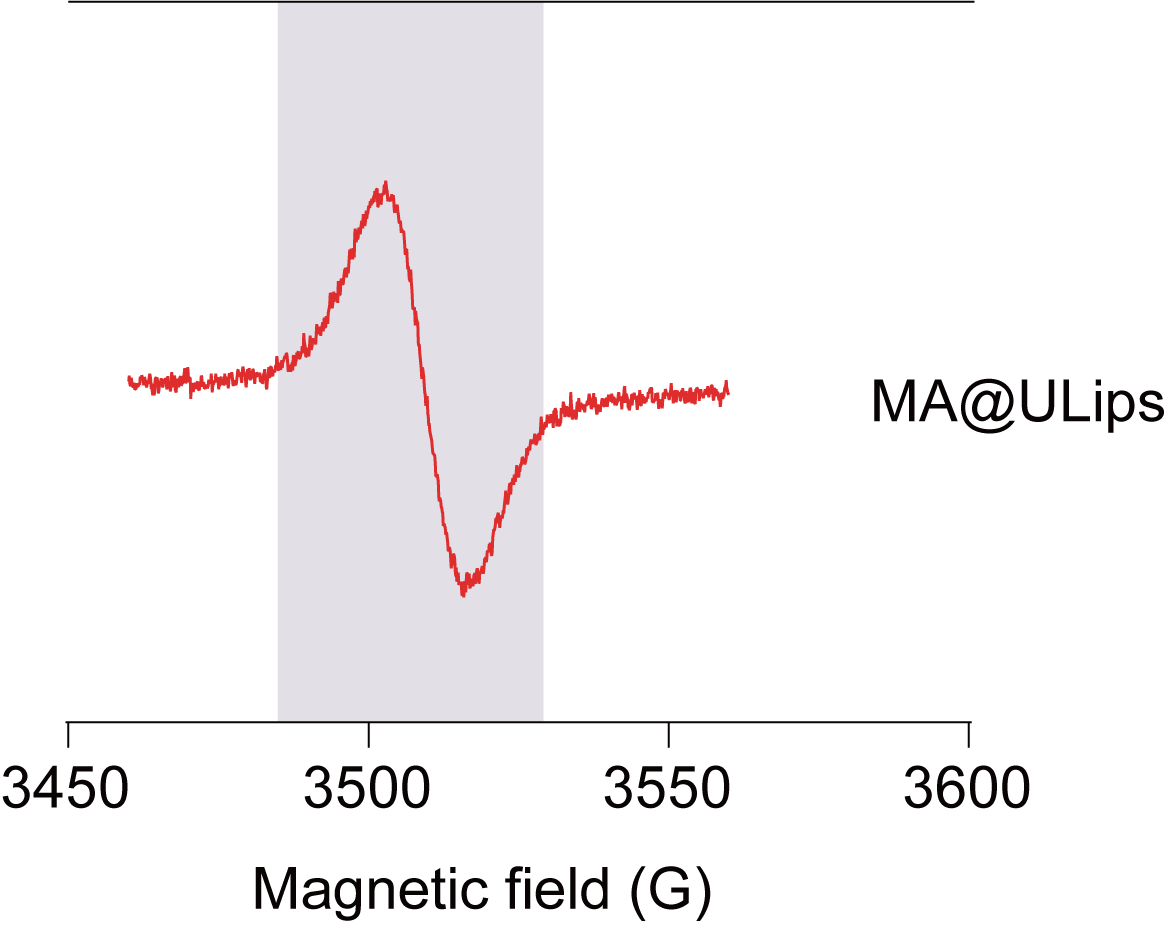


**Fig. S15.** The electron paramagnetic resonance detection of paramagnetic TEMPO radicals in MA@ULips. The light purple background shows the fraction of TEMPO signals.


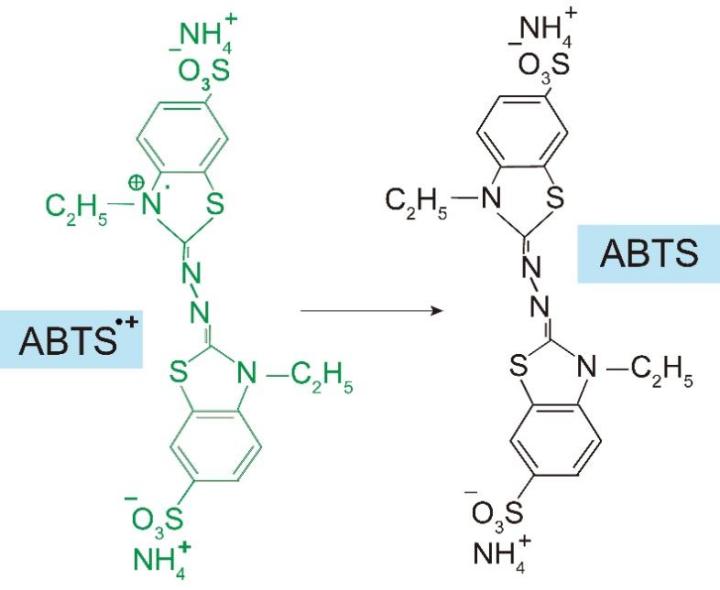


**Fig. S16.** Schematic representation depicting the reaction between ABTS^•+^ and ULips.


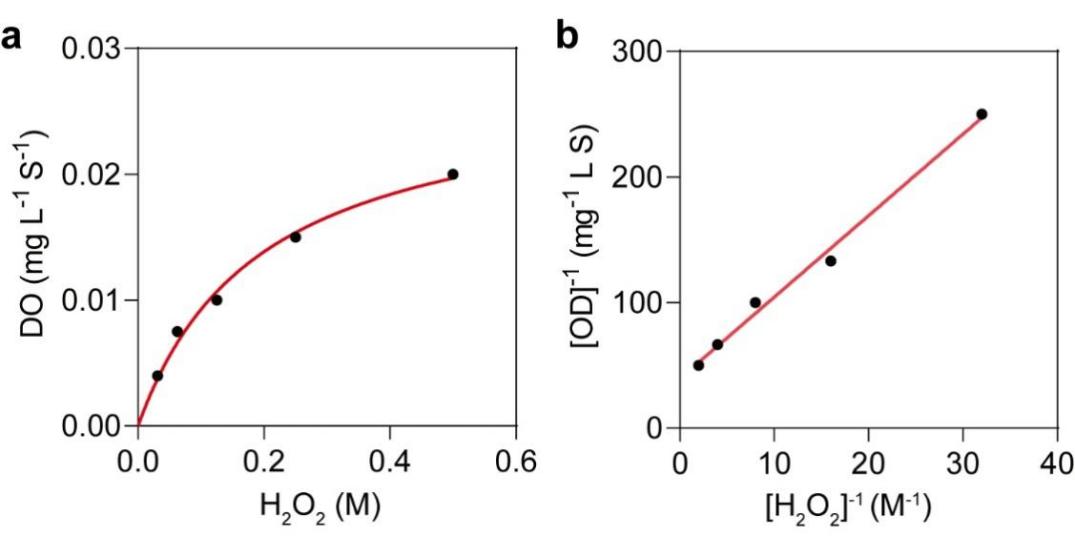


**Fig. S17.** (a) Michaelis-Menten curve and (b) Lineweaver-Burk plots of the CAT-like activity of ULips. The Km value was 0.58 M, and the Vmax value was 3.3 M/s.


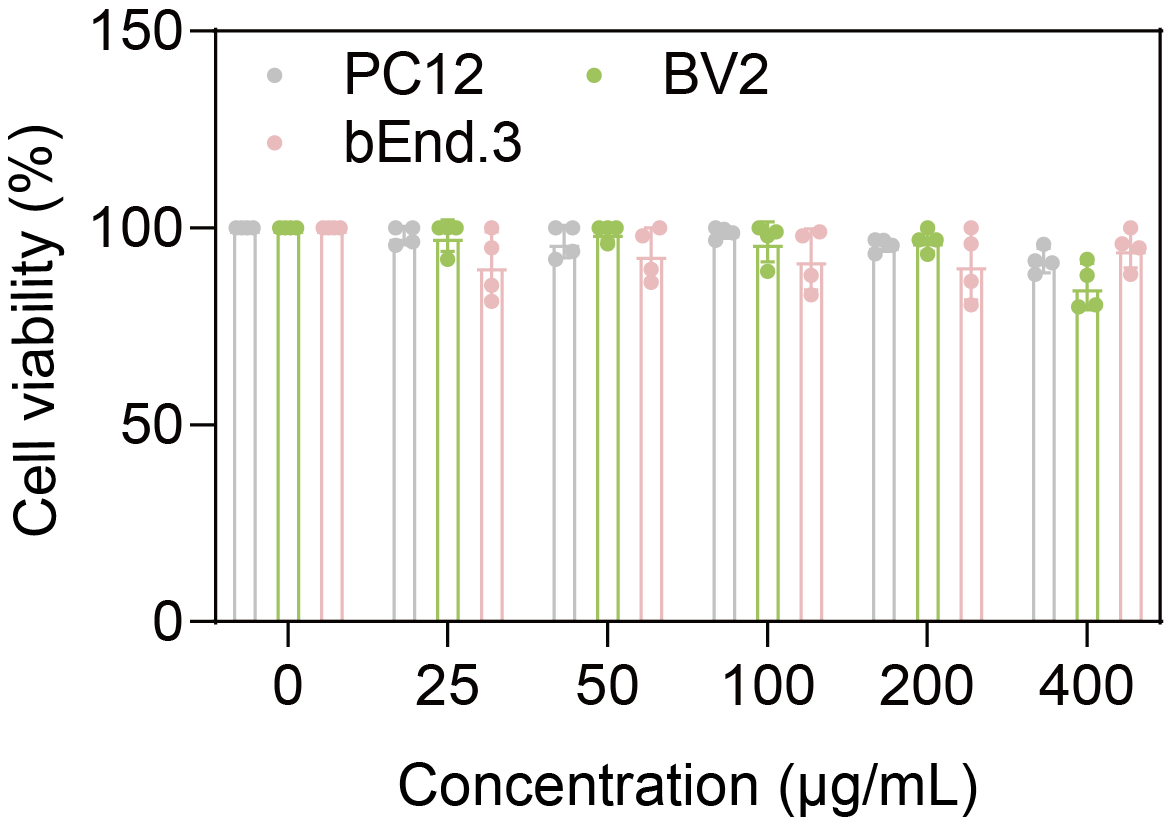


**Fig. S18.** The viability of PC12, BV2, and bEnd.3 cells after treatment with MA@ULips at different concentrations (n = 4). Data are presented as mean ± SD.

**
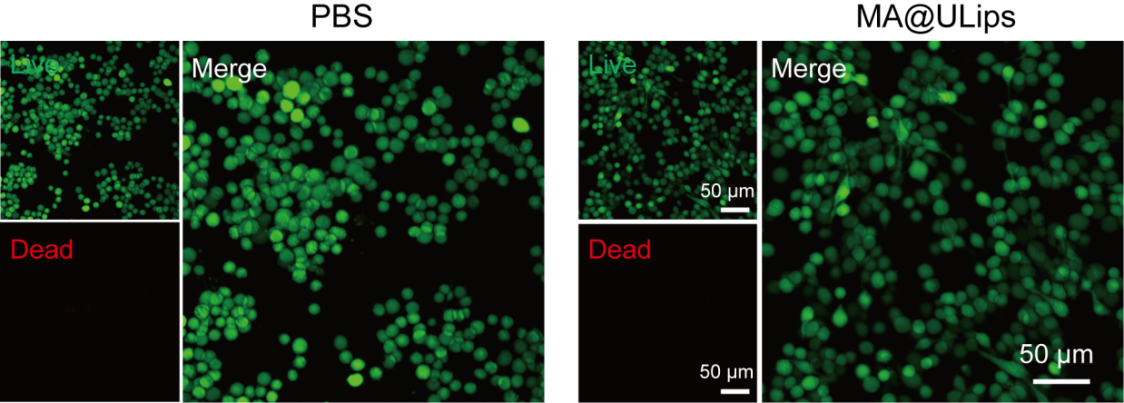
**

**Fig. S19.** Live/dead staining of PC12 cells after treatment with MA@ULips.


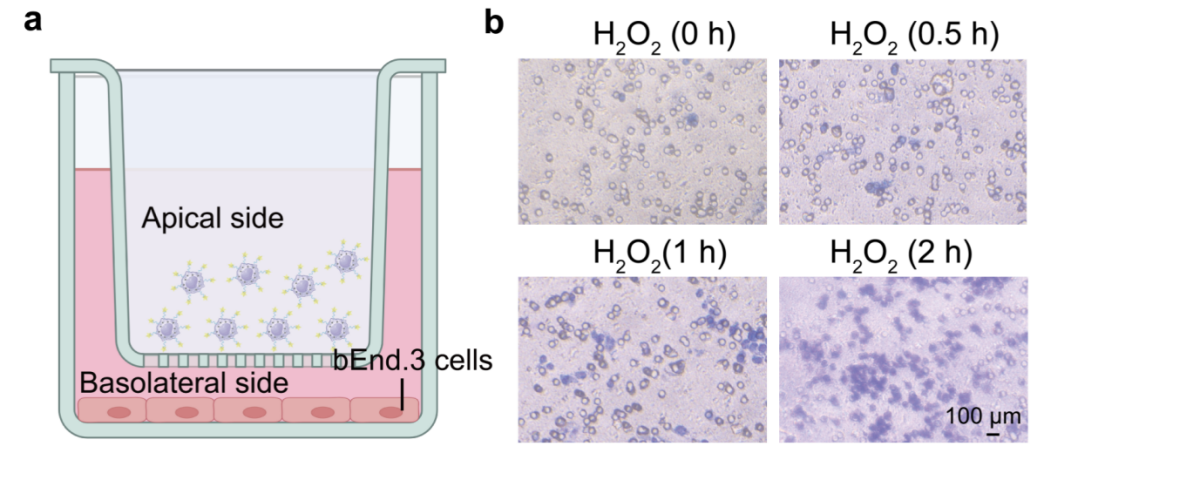


**Fig. S20. The inflammation-targeting capability of MA@ULips.** (a) Schematic illustration of the inflammation tendency of MA@ULips in vitro. (b) Representative images of the migration of MA@ULips after the bEnd.3 cells treatment with H_2_O_2_ at different timepoints.


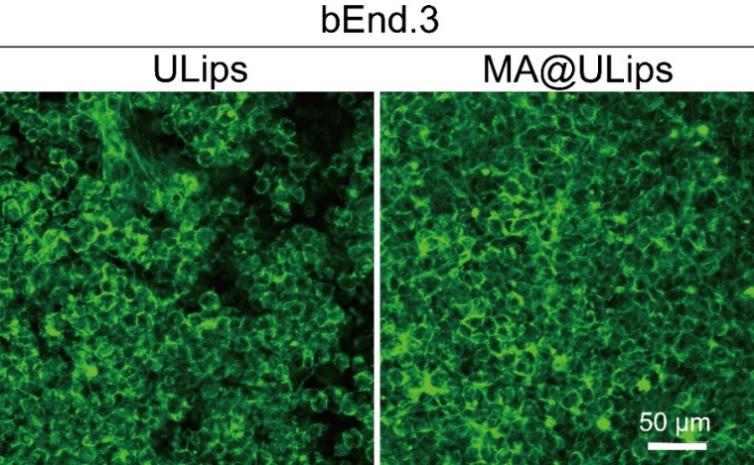


**Fig. S21.** CLSM images of bEnd.3 cell monolayer stained with iFluor™ 488 phalloidin.


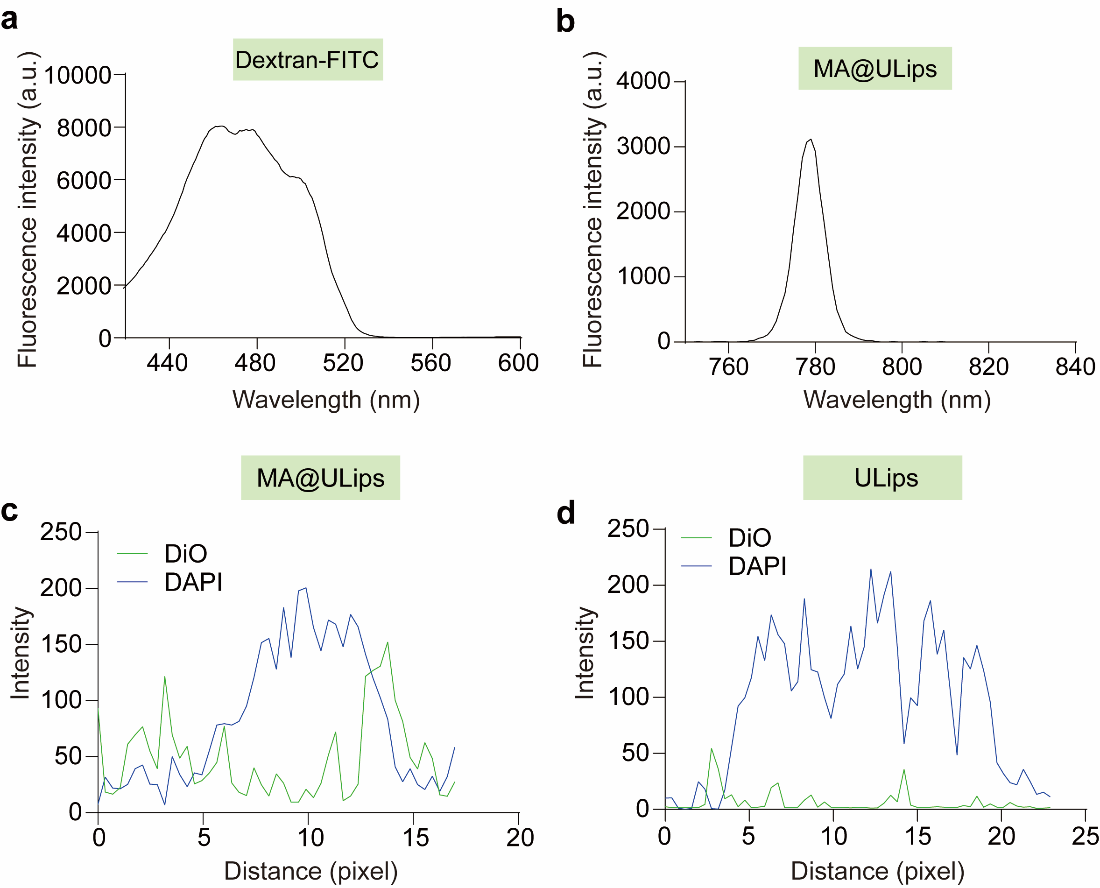


**Fig. S22.** (a, b) Fluorescence intensity in the lower transwell chamber medium after OGD/R exposure in two groups. (c, d) Colocalization analysis of the fluorescence signal changes by CLSM (green: DiO-labeled ULips, blue: DAPI-labeled cell nucleus).


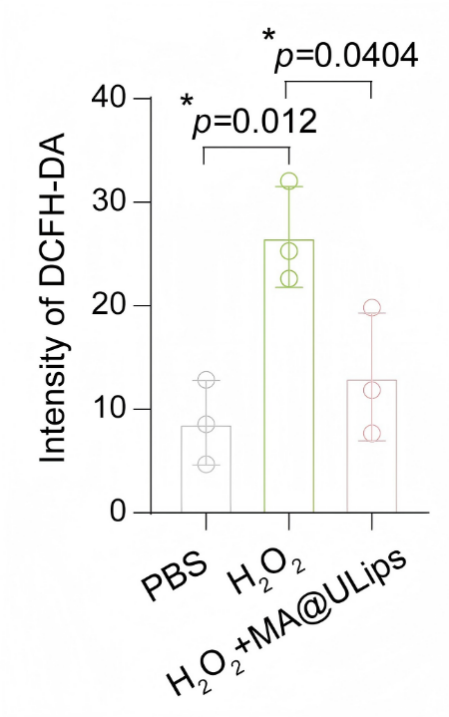


**Fig. S23.** The fluorescence intensities of the corresponding CLSM images of intracellular ROS level in PC12 cells after different treatments (n = 3). Data are presented as mean ± SD. Statistical significance was calculated by one-way ANOVA with Tukey's multiple comparisons test. **p* < 0.05.


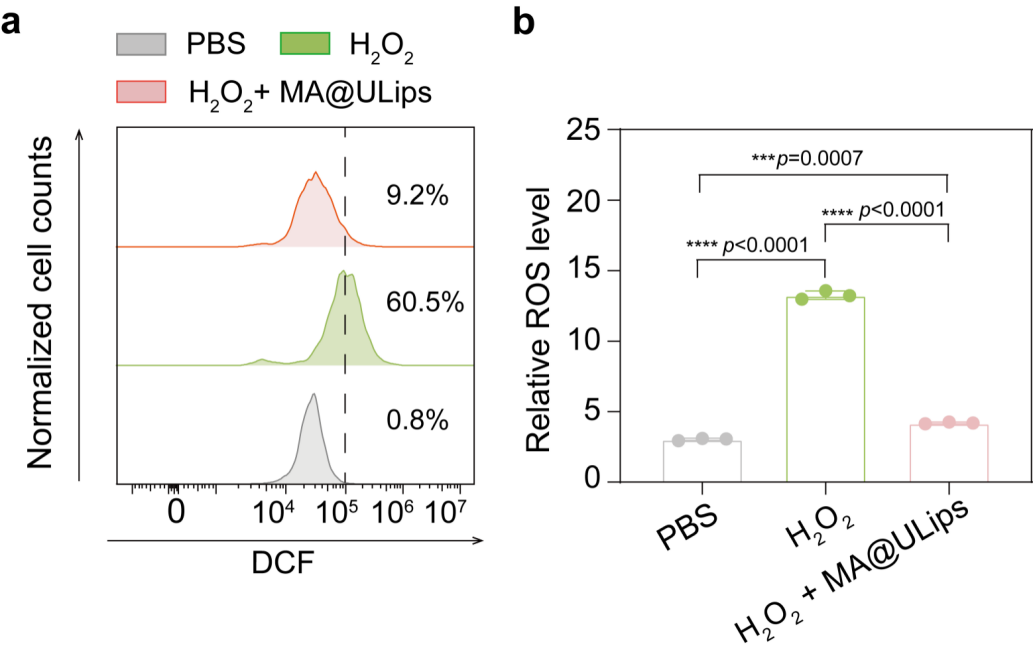


**Fig. S24. Effect of MA@ULips on regulation of intracellular ROS level in PC12 cells.** (a) Flow cytometry analysis and (b) corresponding quantification analysis of intracellular ROS level in PC12 cells after various treatments (n = 3). Data are presented as mean ± SD. Statistical significance was calculated by one-way ANOVA with Tukey's multiple comparisons test. ****p* < 0.001, *****p* < 0.0001.


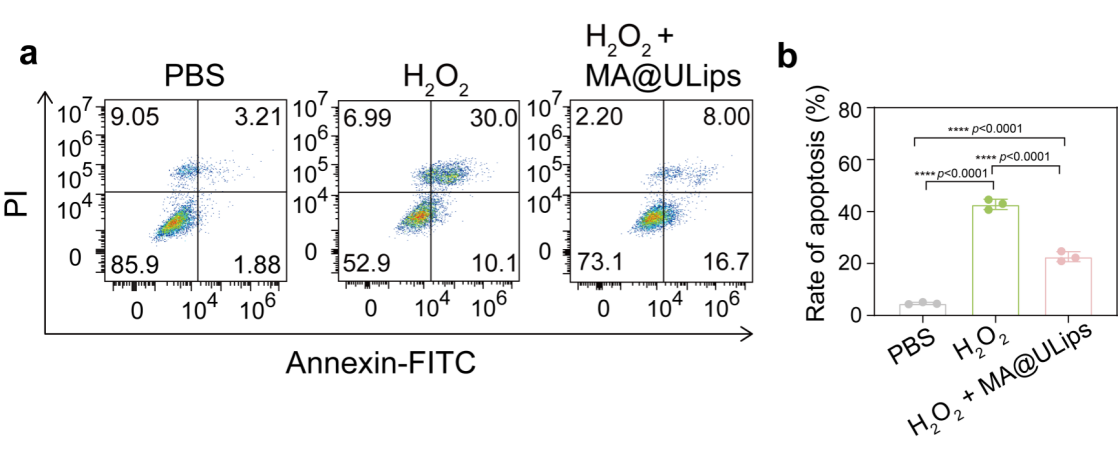


**Fig. S25. Effect of MA@ULips on regulation of rate of apoptosis in PC12 cells.** (a) Flow cytometry analysis and (b) corresponding quantification analysis of PC12 cells co-staining with Annexin V-FITC and PI (n = 3). Data are presented as mean ± SD. Statistical significance was calculated by one-way ANOVA with Tukey's multiple comparisons test. *****p* < 0.0001.


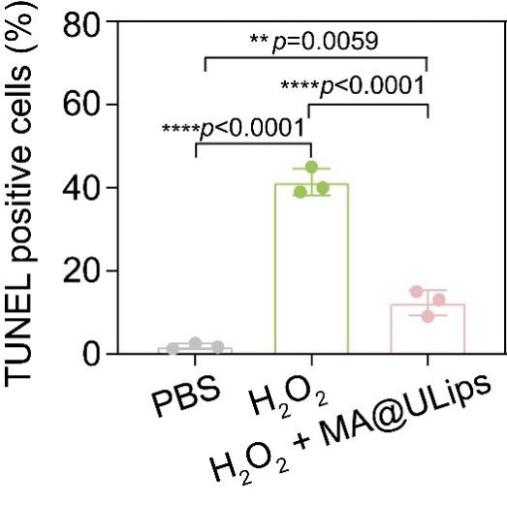


**Fig. S26.** TUNEL positive PC12 cells after various treatments (n = 3). Data are presented as mean ± SD. Statistical significance was calculated by one-way ANOVA with Tukey's multiple comparisons test. ***p* < 0.01, *****p* < 0.0001.


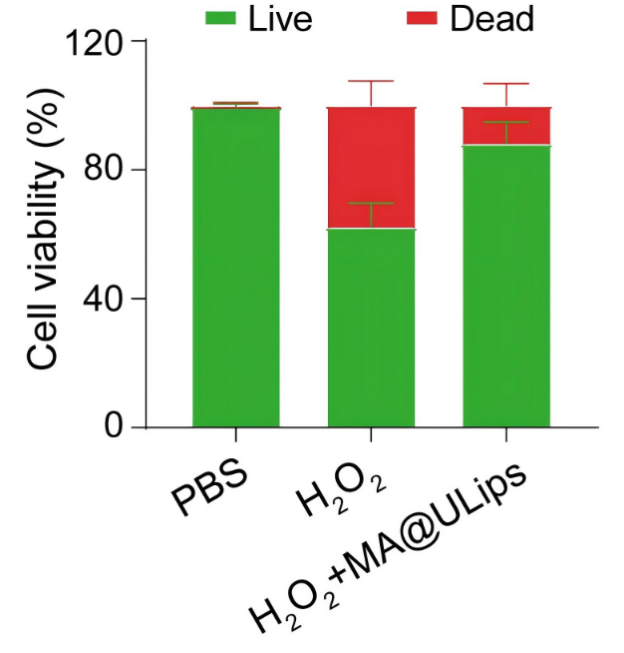


**Fig. S27.** Corresponding quantification analysis of PC12 cells co-staining with Calcein-AM and PI (n = 3). Data are presented as mean ± SD. Statistical significance was calculated by one-way ANOVA with Tukey's multiple comparisons test.


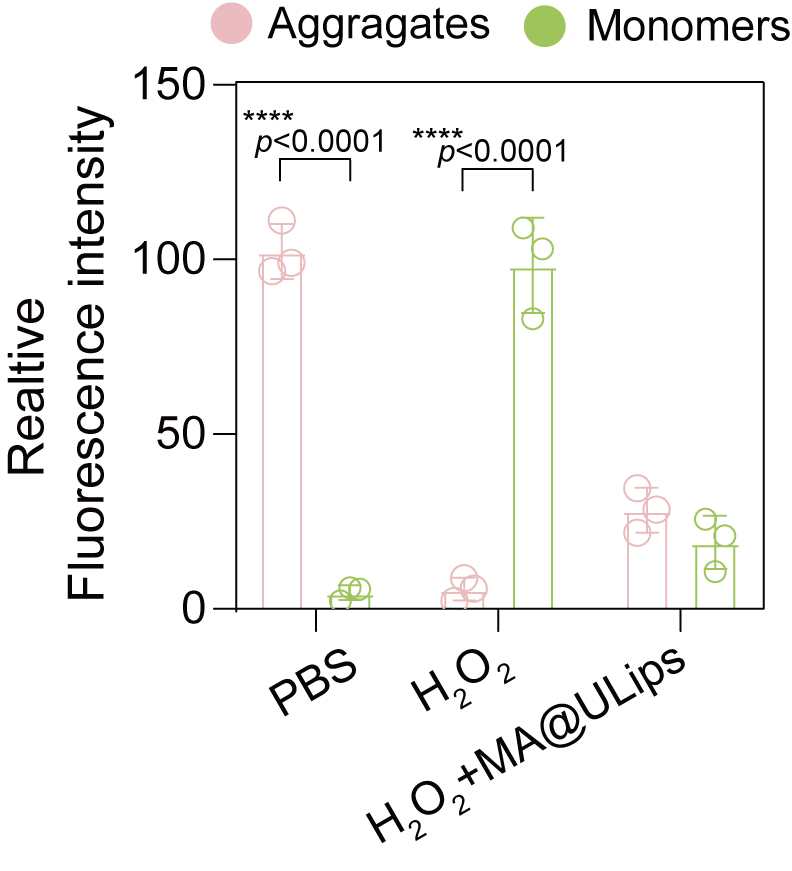


**Fig. S28.** The relative fluorescence intensities of the corresponding CLSM images of MMP changes (Monomers: % fold of OGD/R; Aggregates: % fold of PBS). (n = 3). Data are presented as mean ± SD. Statistical significance was calculated by one-way ANOVA with Tukey's multiple comparisons test. *****p* < 0.0001.


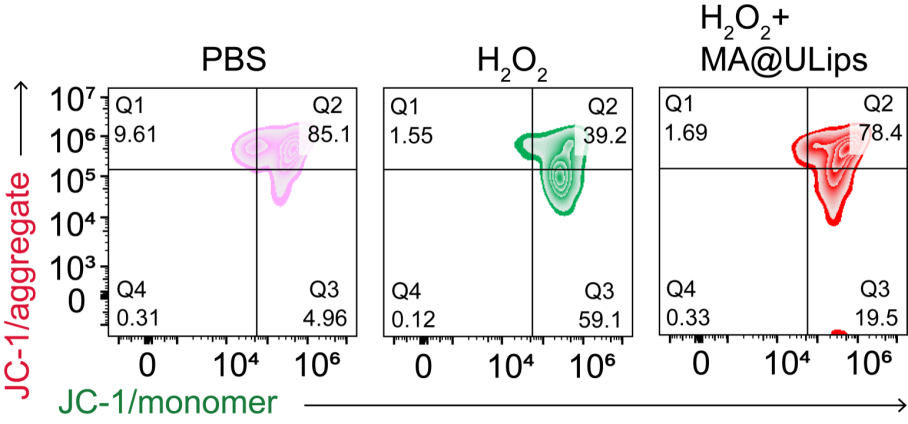


**Fig. S29.** Flow cytometry result of JC-1 monomers and aggregates in PC12 cells after different treatments.

**
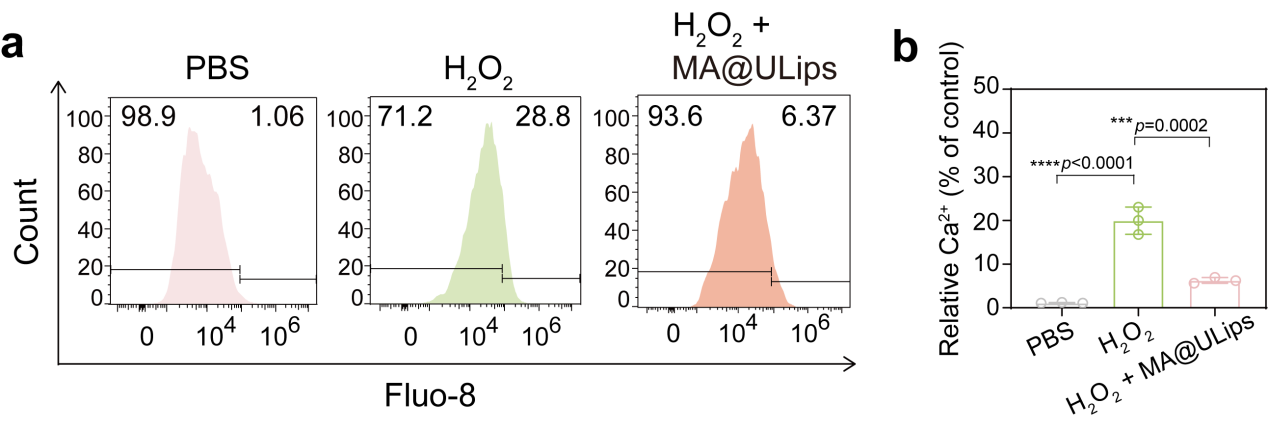
**

**Fig. S30. Effect of MA@ULips on regulation of intracellular calcium concentration in PC12 cells.** (a) Flow cytometry analysis and (b) corresponding quantification of PC12 cells of intracellular calcium concentration by Fluo-8 (n = 3). Data are presented as mean ± SD. Statistical significance was calculated by one-way ANOVA with Tukey's multiple comparisons test. ****p* < 0.001, *****p* < 0.0001.


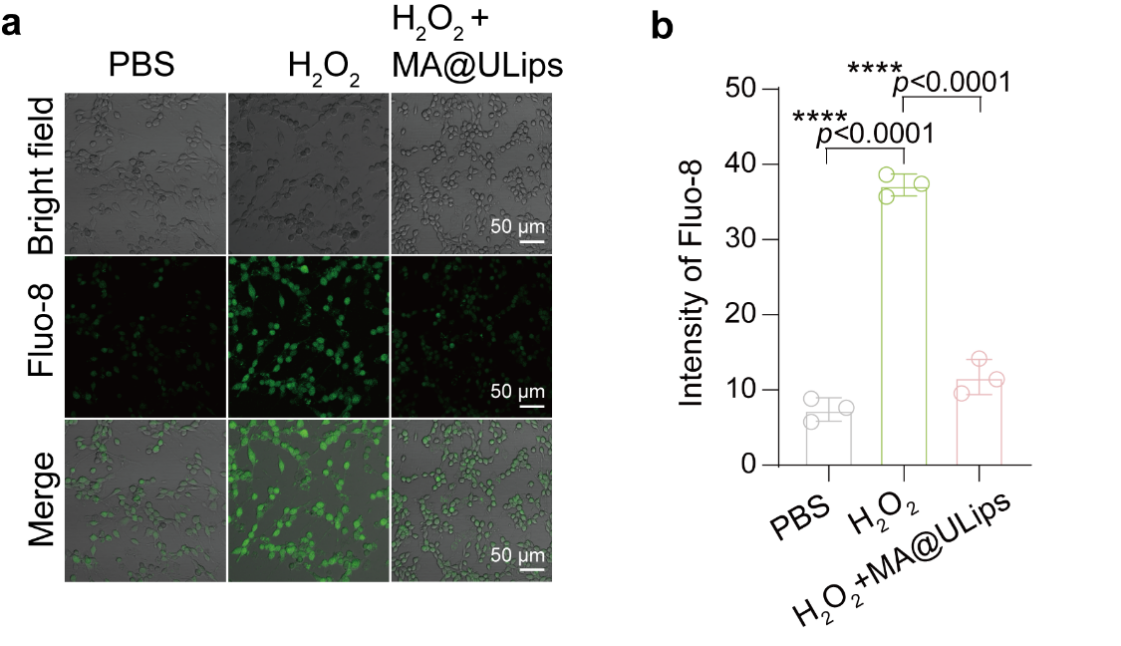


**Fig. S31. Effect of MA@ULips on regulation of intracellular calcium level in PC12 cells.** (a) Confocal images and (b) corresponding fluorescence intensities of CLSM images of intracellular calcium level (n = 3). Data are presented as mean ± SD. Statistical significance was calculated by one-way ANOVA with Tukey's multiple comparisons test. *****p* < 0.0001.


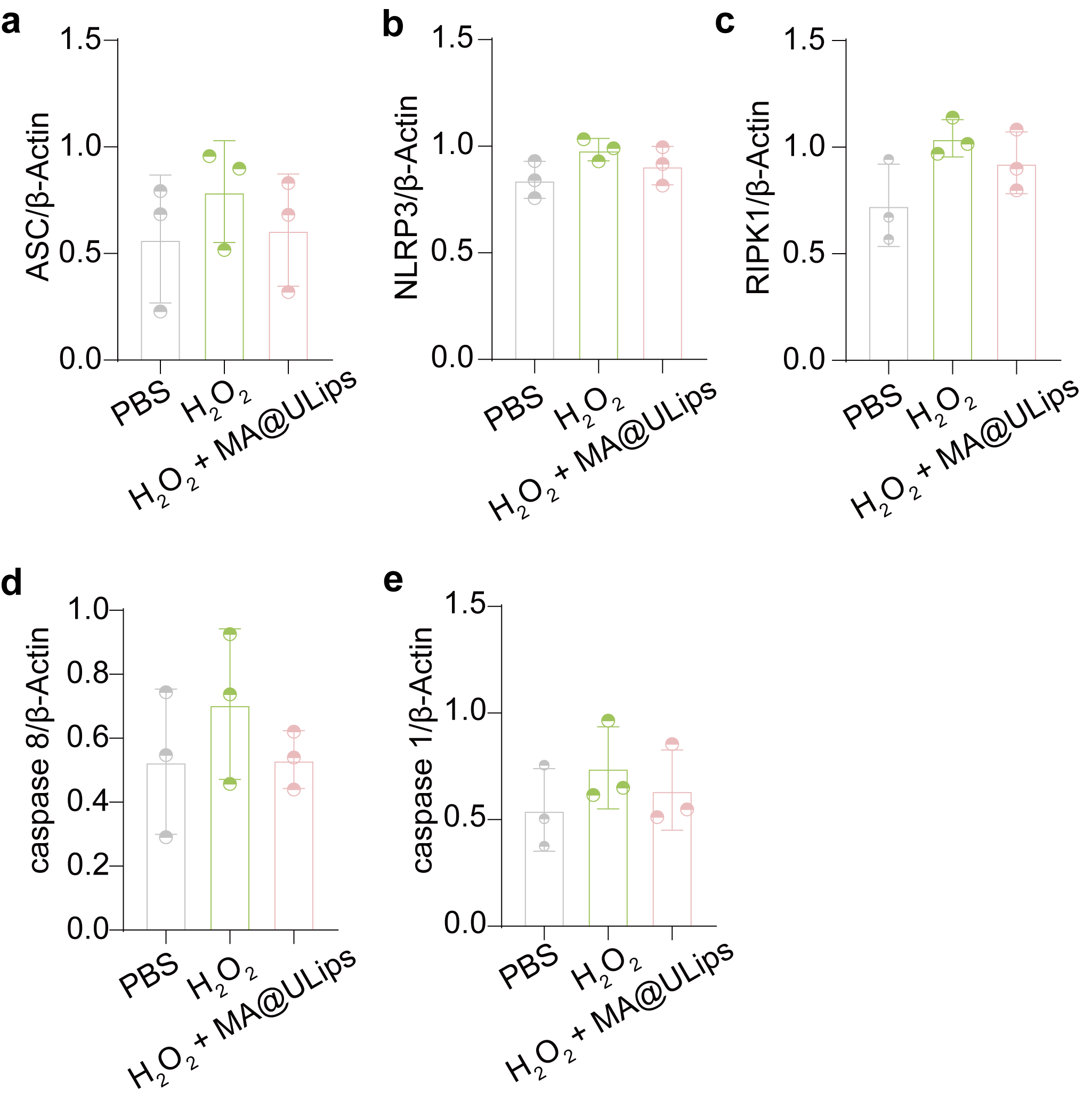


**Fig. S32.** Corresponding analysis of expression ratio of (a) ASC, (b) NLRP3, (c) RIPK1, (d) caspase 8 and (e) caspase 1 (n = 3 independent experiments). Data are presented as mean ± SD.


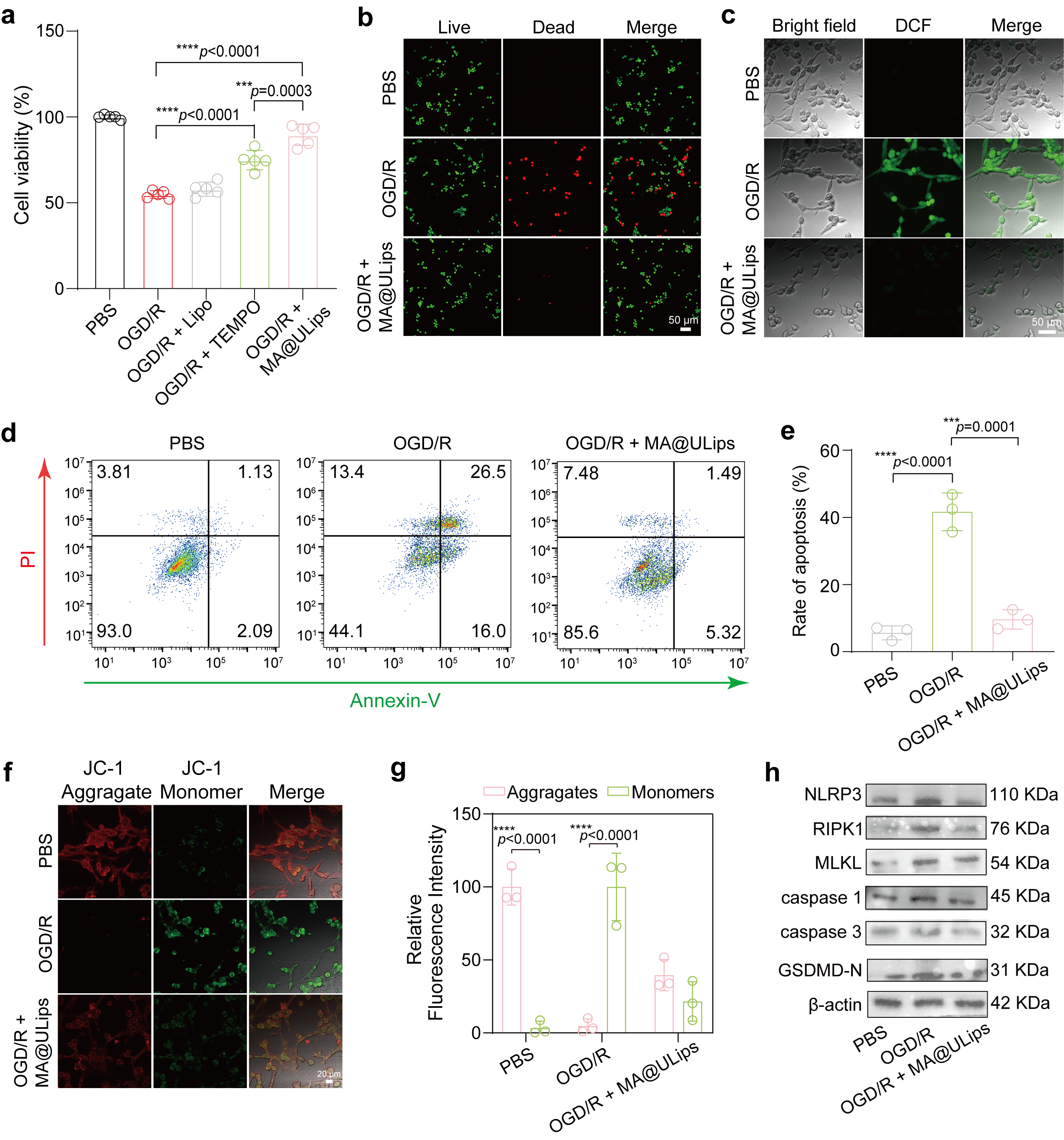


**Fig. S33. In vitro mechanism validation of MA@ULips-inhibited PANoptosis in an OGD/R model**. (a) The PC12 cell viability after various treatments (n = 5). (b) CLSM images of live/dead staining PC12 cells after different treatments. (c) CLSM images of intracellular ROS level in PC12 cells after different treatments. (d) Flow cytometry analysis and (e) corresponding quantification analysis of PC12 cells co-staining with Annexin V-FITC and PI (n = 3). (f) CLSM images of MMP in PC12 cells with various treatments staining by JC-1 probes. (g) The relative fluorescence intensities of the corresponding CLSM images of MMP changes (Monomers: % fold of OGD/R; Aggregates: % fold of PBS). (n = 3). (h) Western blotting analysis of PANoptosis-associated proteins in BV2 cells after various treatments. Data are presented as mean ± SD. Statistical significance was calculated by one-way ANOVA with Tukey's multiple comparisons test. ****p* < 0.001, *****p* < 0.0001.


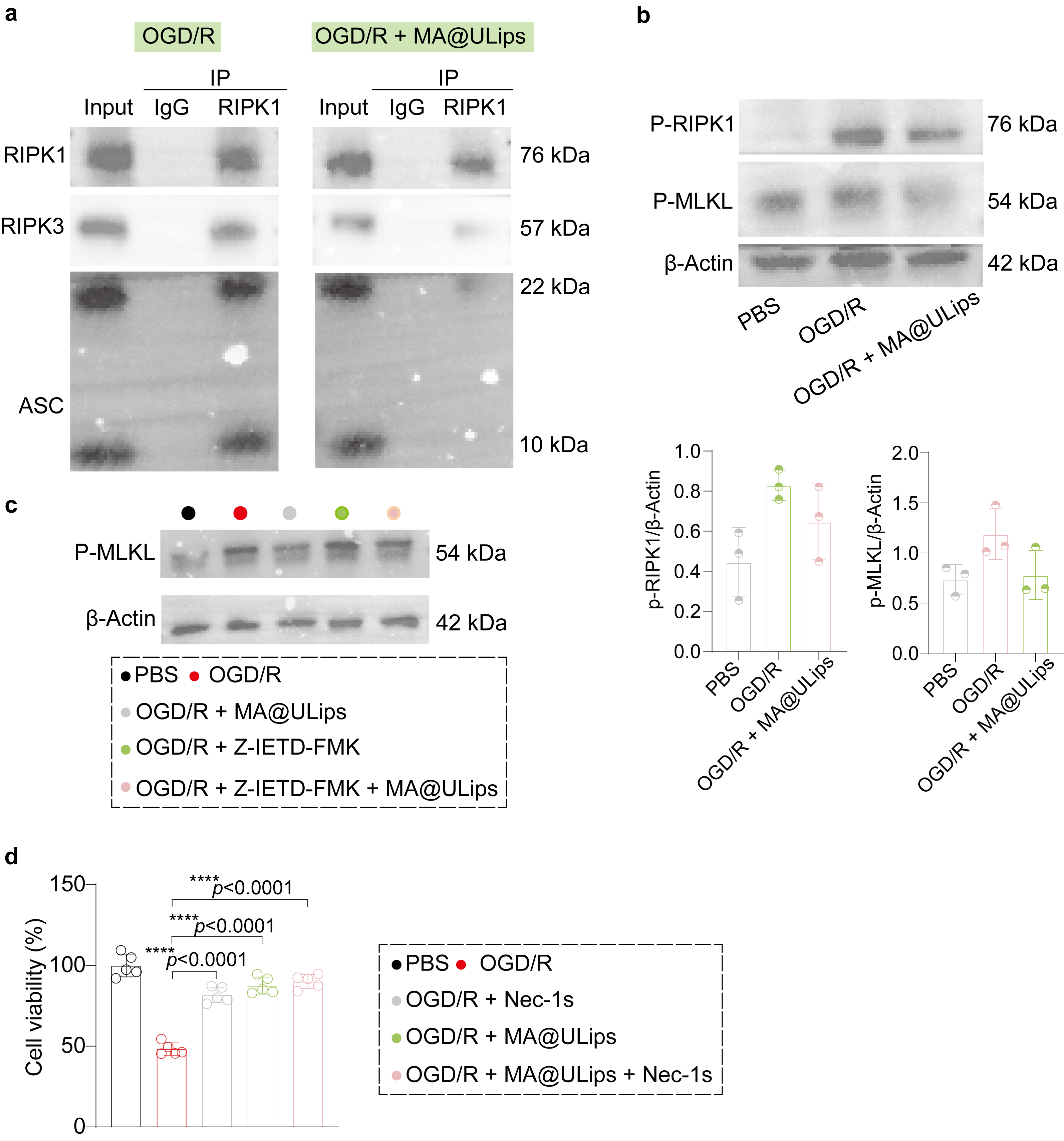


**Fig. S34. Mechanistic validation of RIPK1-PANoptosome targeting.** (a) Co-immunoprecipitation analysis of RIPK1, RIPK3 and ASC with or without MA@ULips treatment. (b) Western blotting analysis of p-RIPK1 (S166) and p-MLKL in BV2 cells after various treatments. (c) Detection of necroptosis in BV2 cells after various treatments. (d) The PC12 cell viability after various treatments (n = 5). Data are presented as mean ± SD. Statistical significance was calculated by one-way ANOVA with Tukey's multiple comparisons test. *****p* < 0.0001.


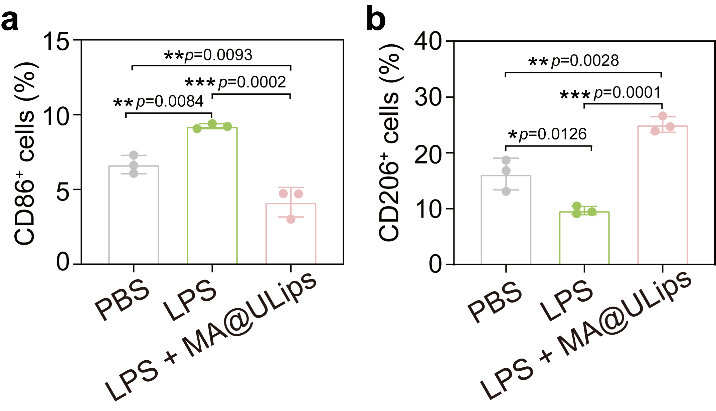


**Fig. S35.** Corresponding quantification analysis of (a) M1 microglia (CD86^+^) and (b) M2 microglia (CD206^+^) after different treatments, followed by stimulation of LPS (n = 3). Data are presented as mean ± SD. Statistical significance was calculated by one-way ANOVA with Tukey's multiple comparisons test. **p* < 0.05, ***p* < 0.01, ****p* < 0.001.


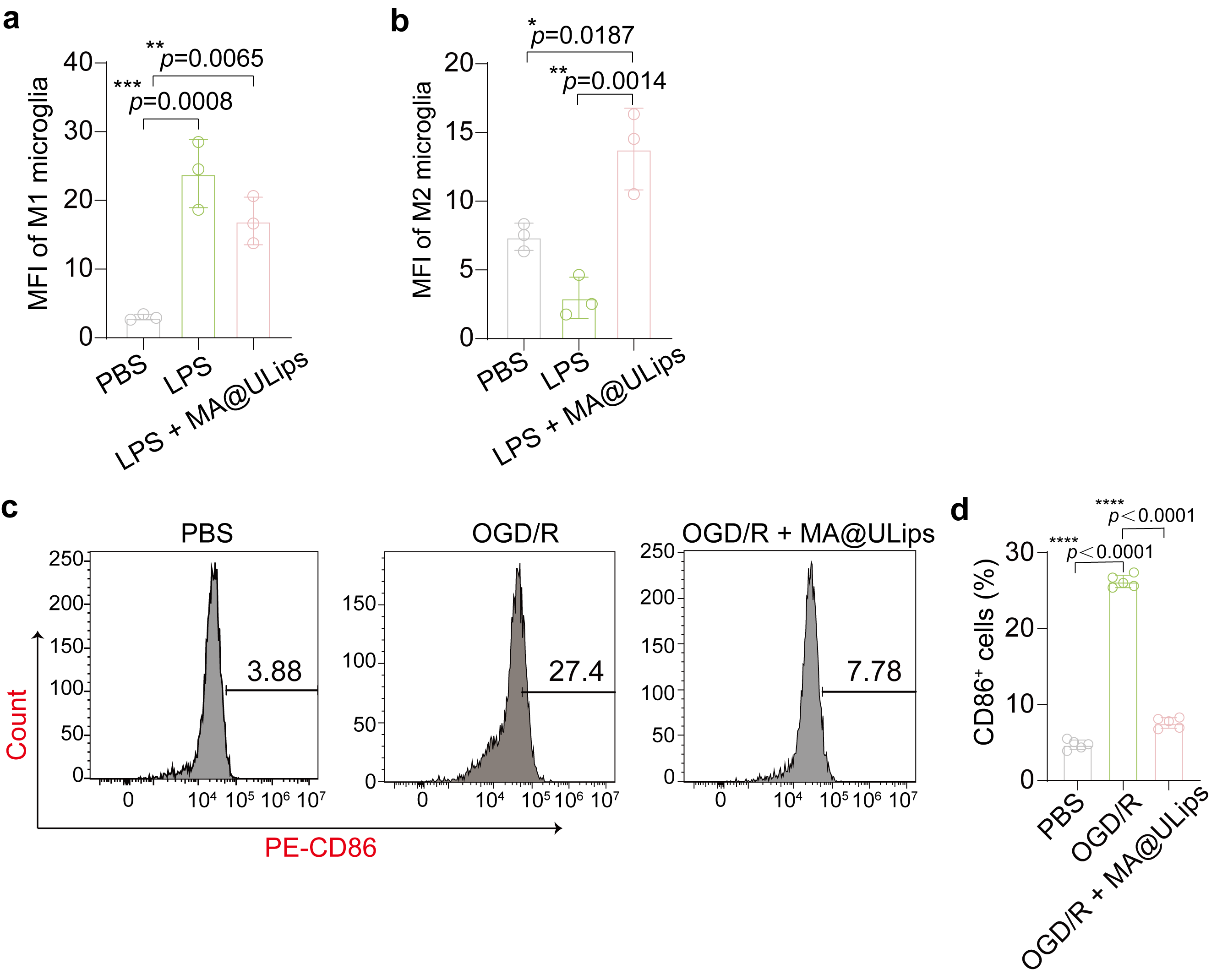


**Fig. S36.** Quantification of the mean fluorescence intensity (MFI) of (a) M1 microglia and (b) M2 microglia after different treatments using Image J software (n = 3). (c) Flow cytometry analysis of BV2 cellular CD86 levels after different treatments. (d) Corresponding quantification analysis of M1 microglia (CD86^+^) after different treatments (n = 5). Data are presented as mean ± SD. Statistical significance was calculated by one-way ANOVA with Tukey's multiple comparisons test. **p* < 0.05, ***p* < 0.01, ****p* < 0.001, *****p* < 0.0001.

**
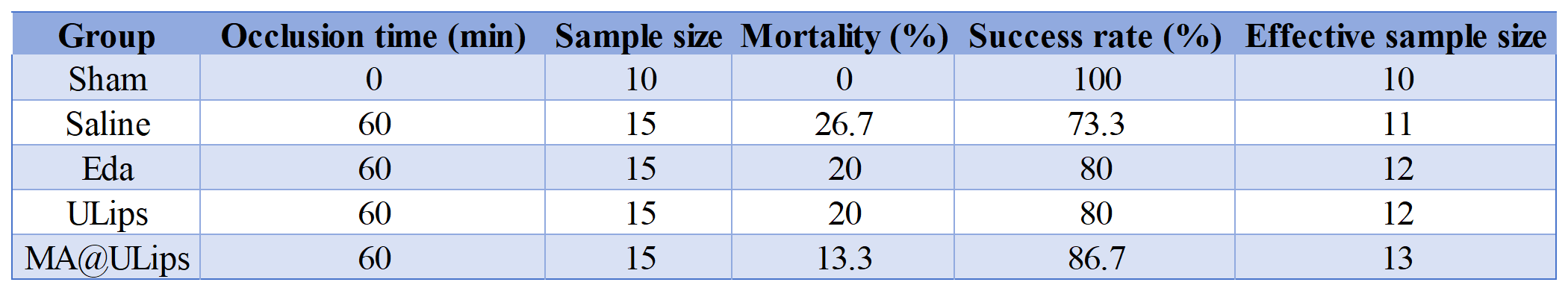
**

**Fig. S37.** The specific mortality and success rates for modeling in each group of mice.

**
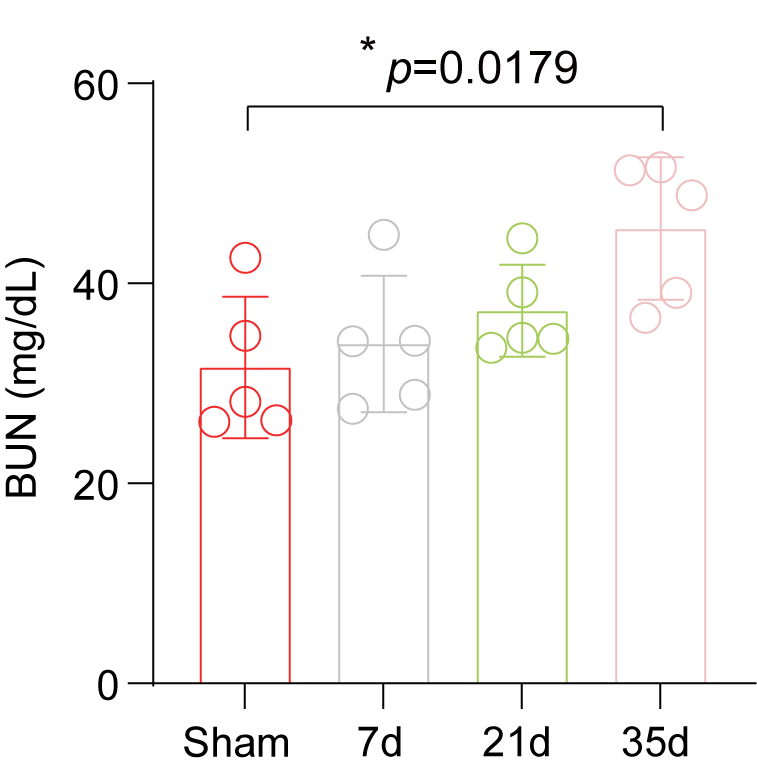
**

**Fig. S38.** Biochemical level of BUN at 7, 21 and 35 days post-injection (n = 5). Data are presented as mean ± SD. Statistical significance was calculated by one-way ANOVA with Tukey's multiple comparisons test. **p* < 0.05.


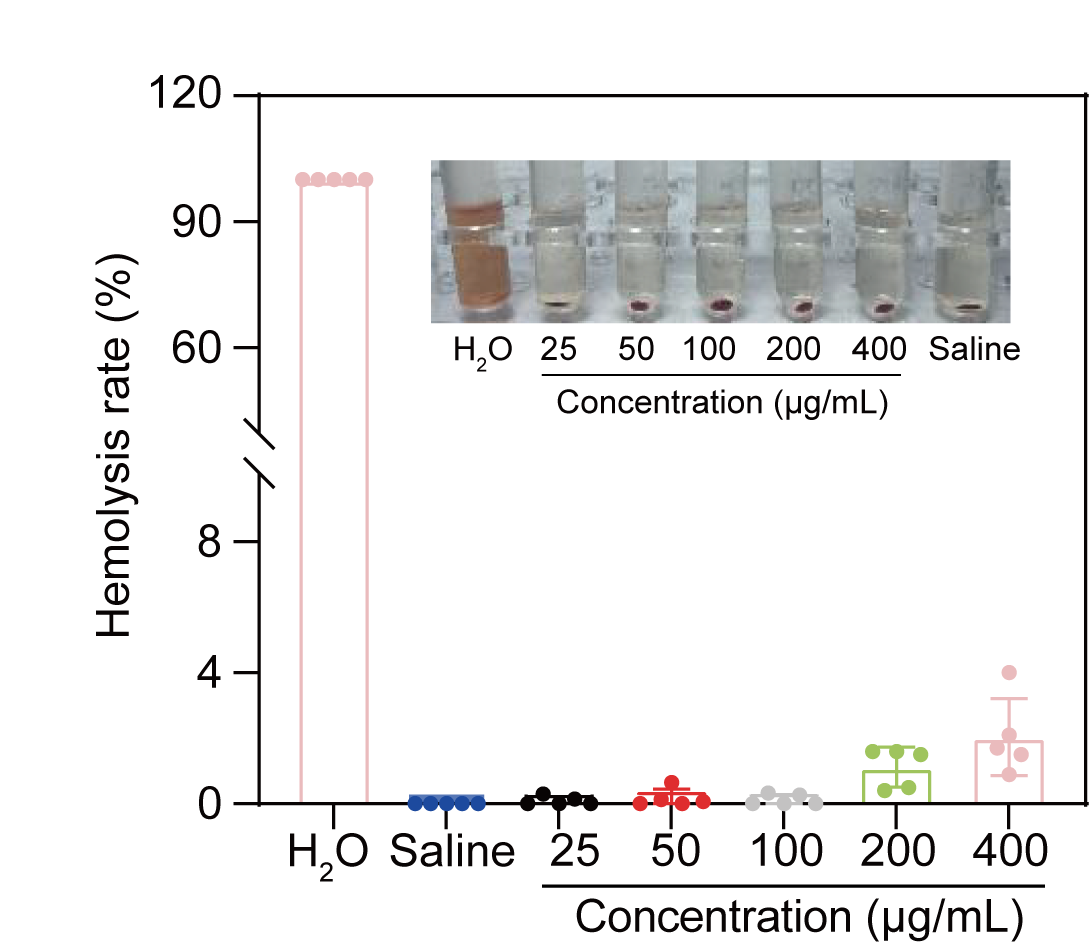


**Fig. S39.** In vitro hemolysis test of MA@ULips (n = 5). Data are presented as mean ± SD.


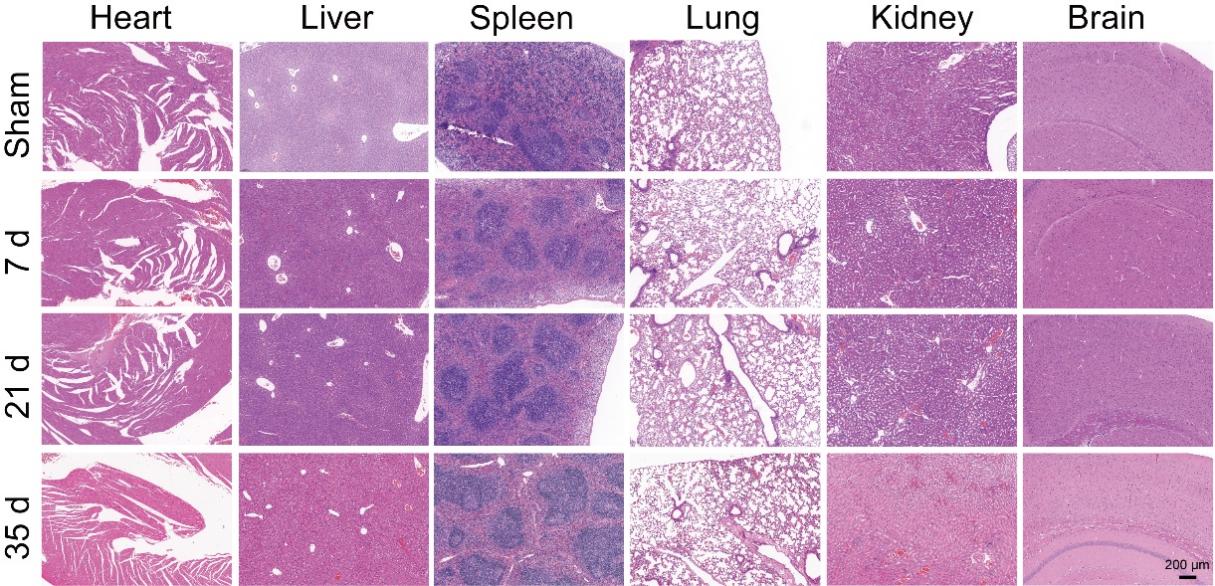


**Fig. S40.** H&E staining of the heart, liver, spleen, lung, kidney, and brain from mice at different timepoints following intravenous injection of MA@ULips.


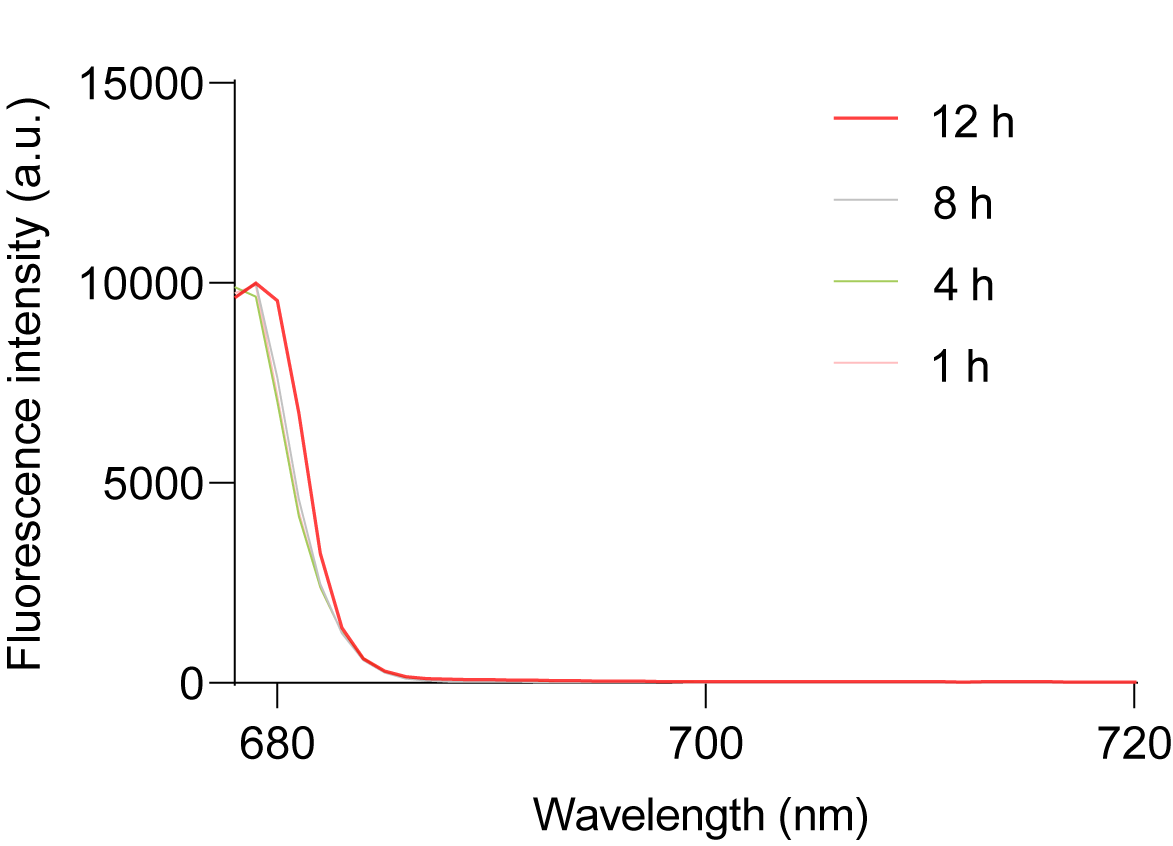


**Fig. S41.** Fluorescence intensity of free Cy5.5 at different timepoints.


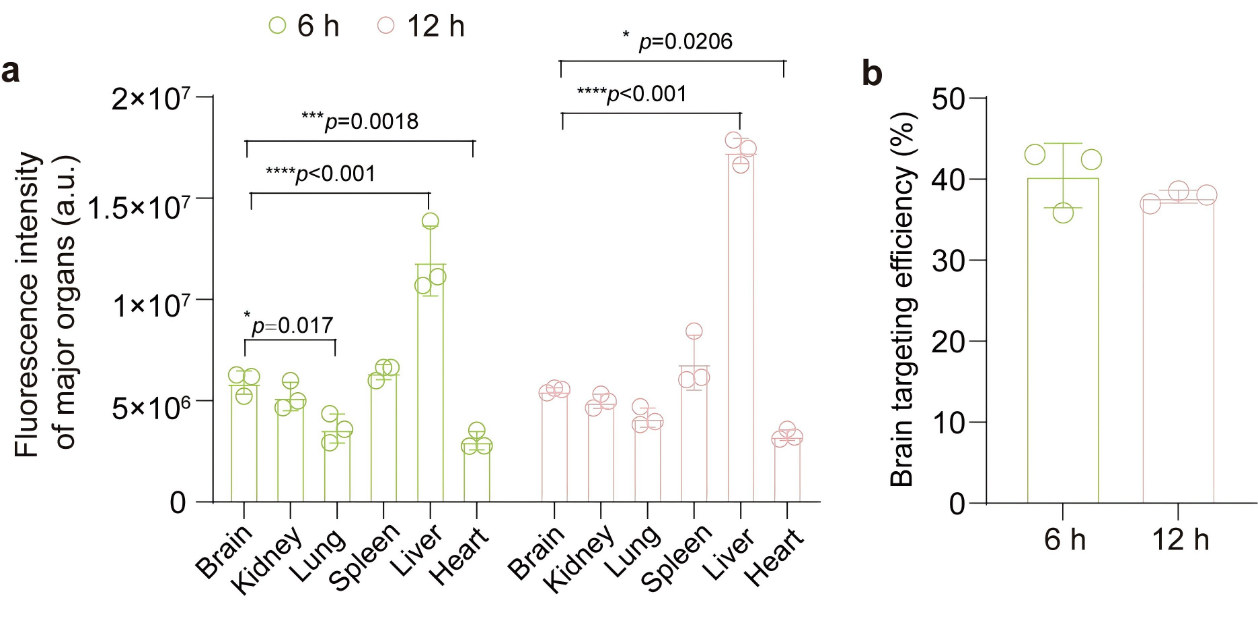


**Fig. S42.** **Evaluation of brain-targeting efficiency.** (a) Fluorescence intensity of major organs at 6 and 12 h after injection of MA@ULips-DiR. (b) Brain targeting efficiency at 6 and 12 h. (n = 3). Data are presented as mean ± SD. Statistical significance was calculated by one-way ANOVA with Tukey's multiple comparisons test. **p* < 0.05, ****p* < 0.001, *****p* < 0.0001.


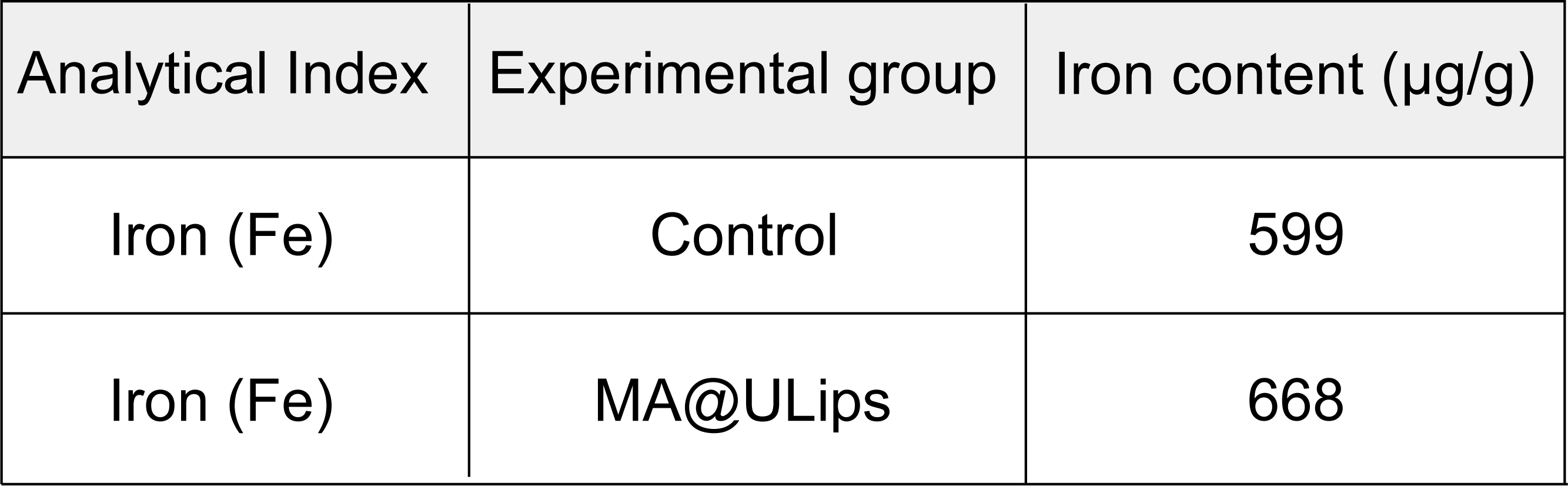


**Fig. S43.** Inductively coupled plasma mass spectrometry (ICP-MS) for iron quantification in control and MA@ULips groups.


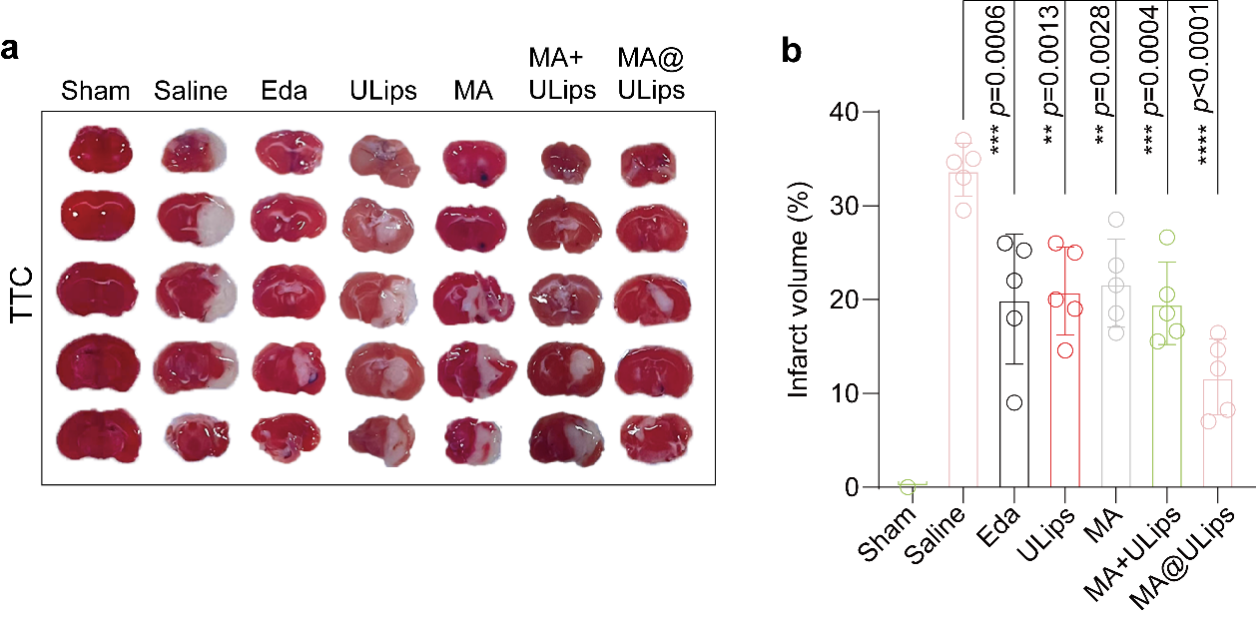


**Fig. S44.** (a) Representative TTC staining images of brain slices after different treatments and (b) corresponding quantification of infarct volume of brain slices treated with different formulations (n = 5). Data are presented as mean ± SD. Statistical significance was calculated by one-way ANOVA with Tukey's multiple comparisons test. ***p* < 0.01, ****p* < 0.001, *****p* < 0.0001.


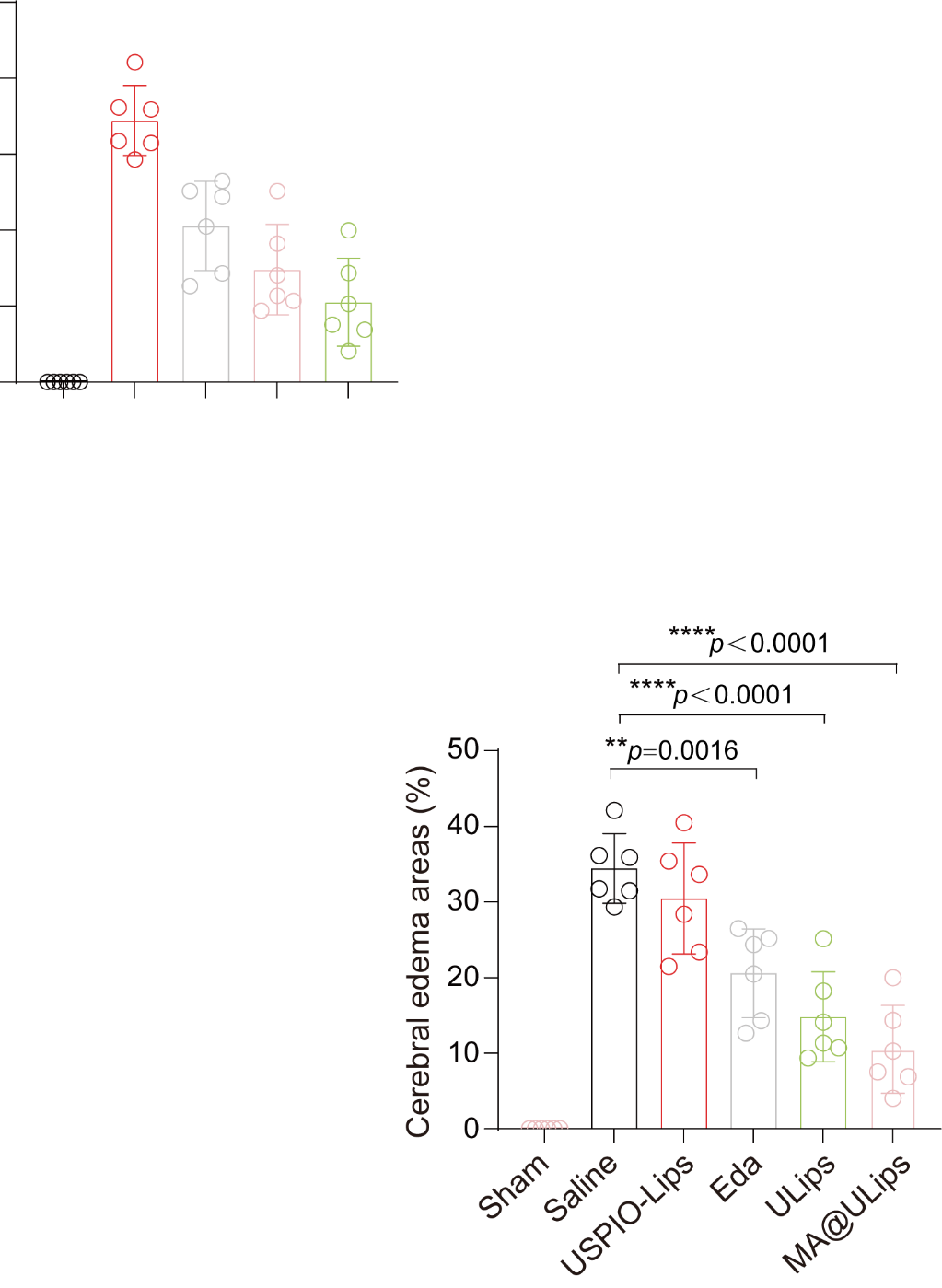


**Fig. S45.** Quantification of cerebral edema areas under different formulations (n = 6). Data are presented as mean ± SD. Statistical significance was calculated by one-way ANOVA with Tukey's multiple comparisons test. ***p* < 0.01, *****p* < 0.0001.


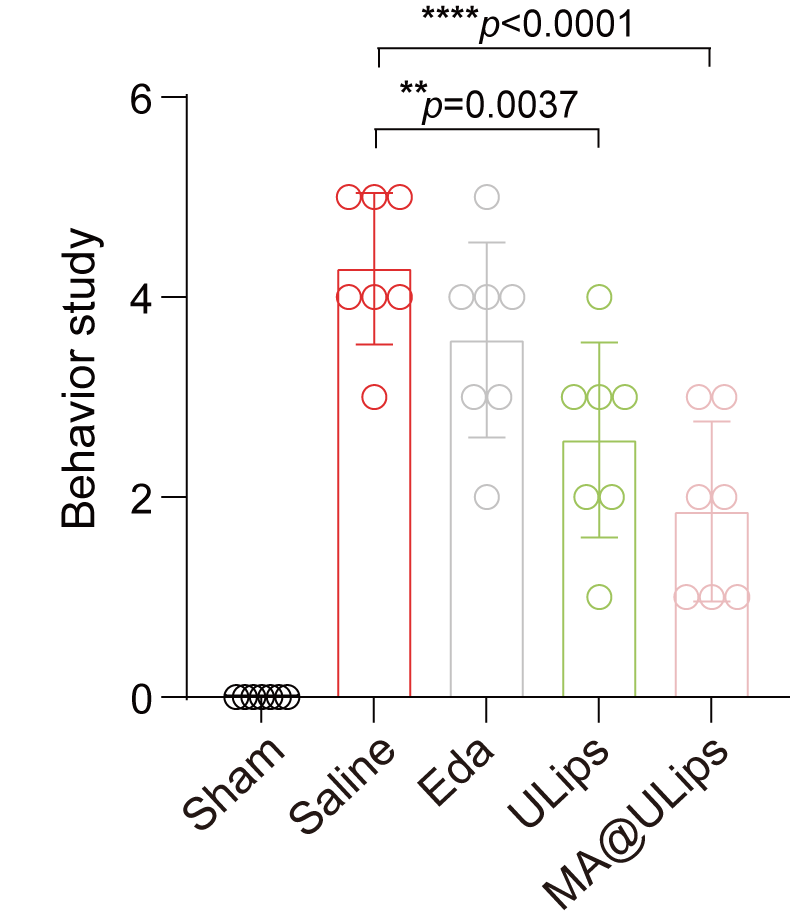


**Fig. S46.** Neurological deficit scores after different treatments (n = 7). Data are presented as mean ± SD. Statistical significance was calculated by one-way ANOVA with Tukey's multiple comparisons test. ***p* < 0.01, *****p* < 0.0001.


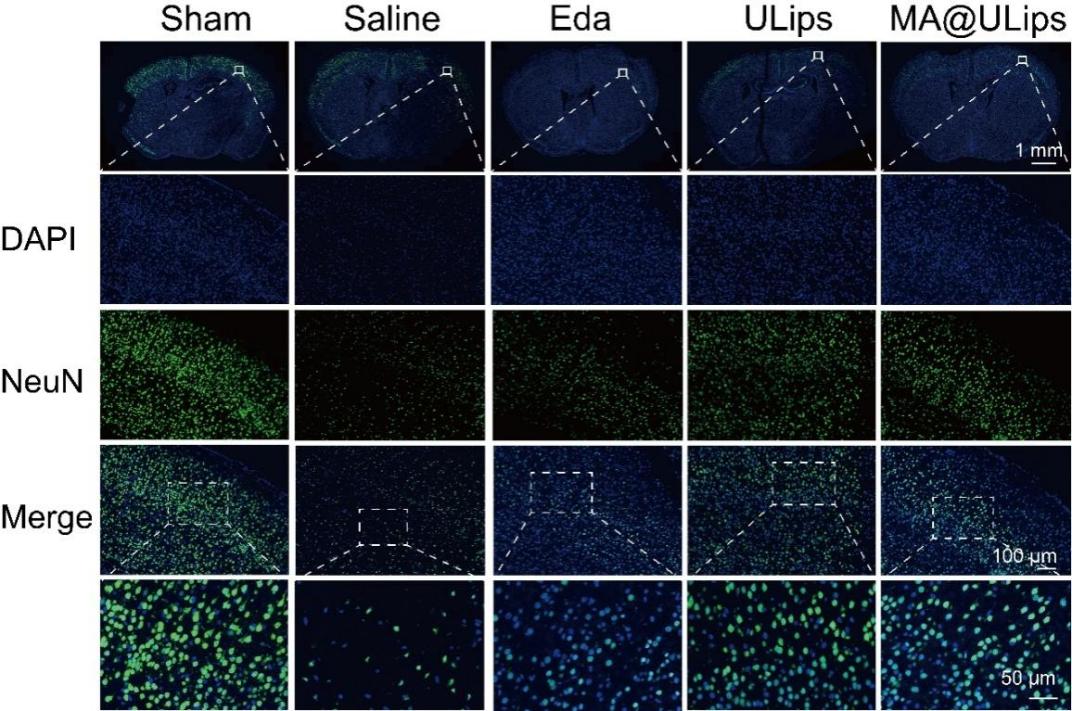


**Fig. S47.** Immunofluorescence staining of NeuN in ischemic brains after various treatments.


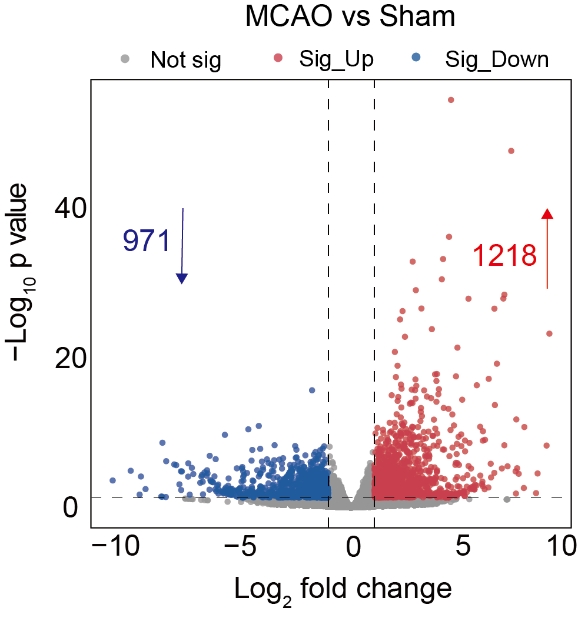


**Fig. S48.** Volcano diagram of differentially expressed genes between the MCAO group and the sham group.


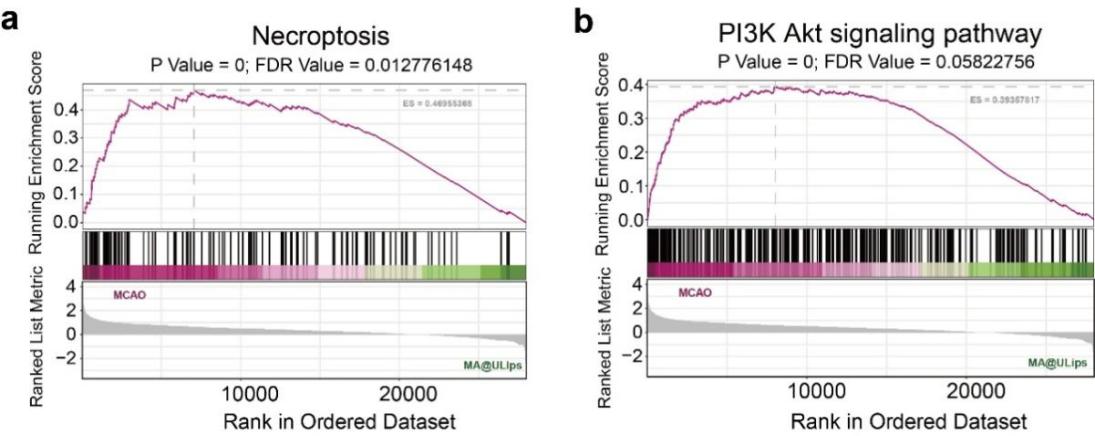


**Fig. S49.** GSEA of necroptosis and PI3K Akt signaling pathway.

**
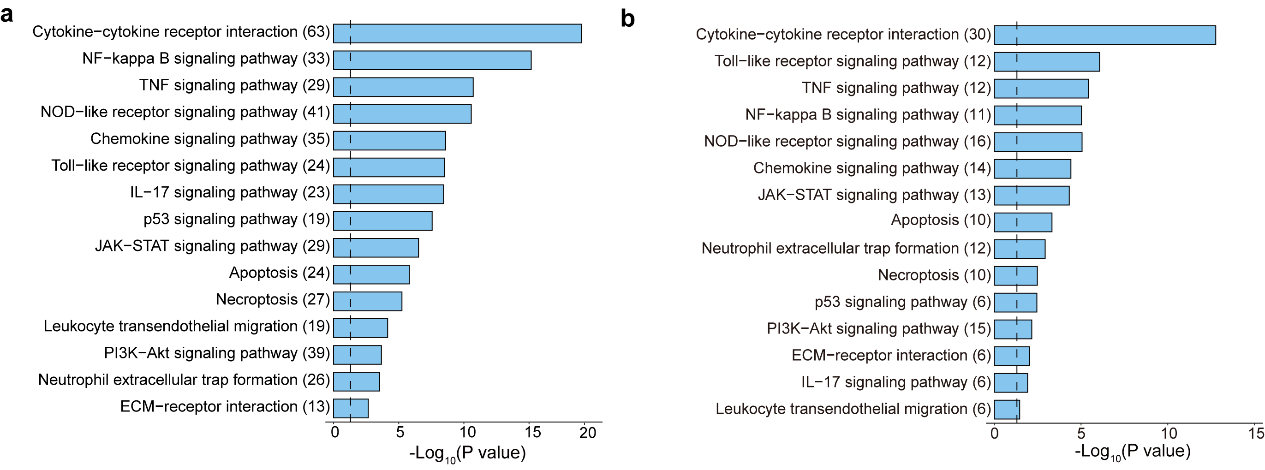
**

**Fig. S50.** Upregulated pathways (a) between the MCAO and the sham groups, and (b) between the MCAO and MA@ULips-treated groups.


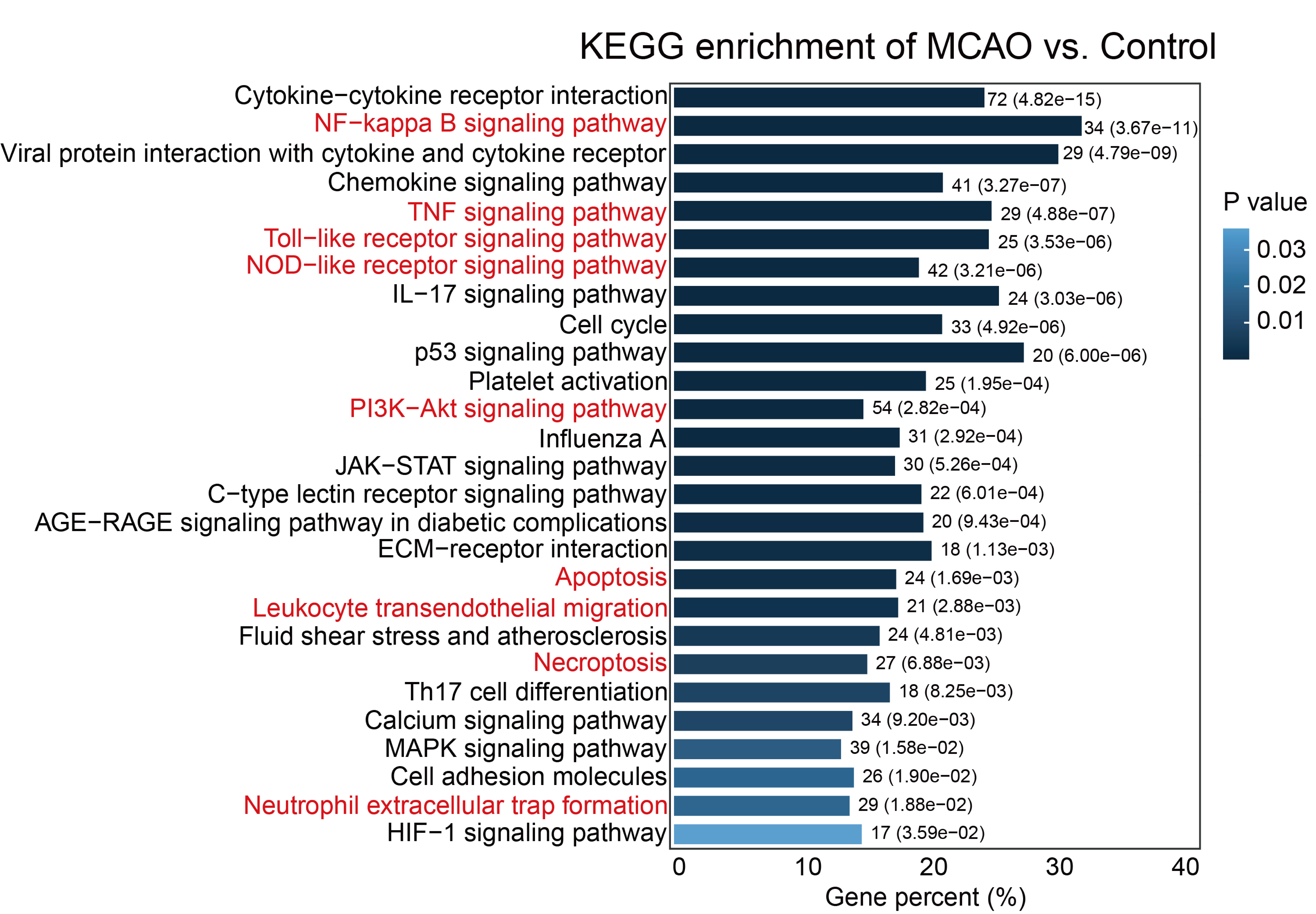


**Fig. S51.** KEGG analysis based on differentially expressed genes between MCAO and Control groups.


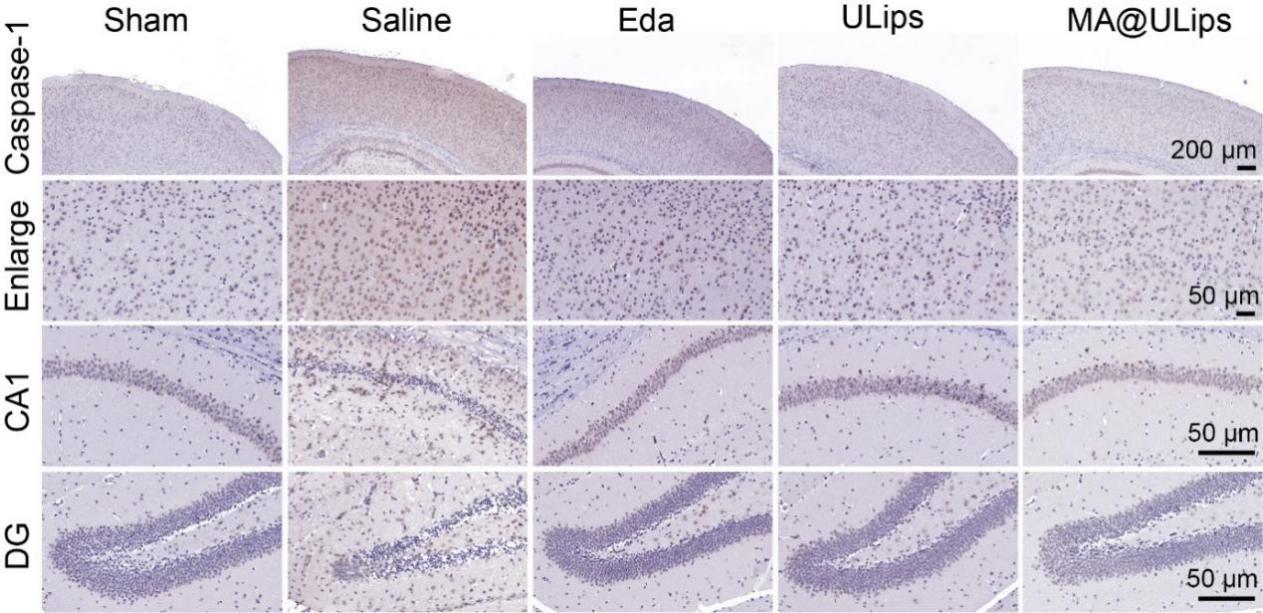


**Fig. S52.** Immunohistochemical staining for caspase-1 expression in the ischemic cortex and hippocampus area after various treatments.


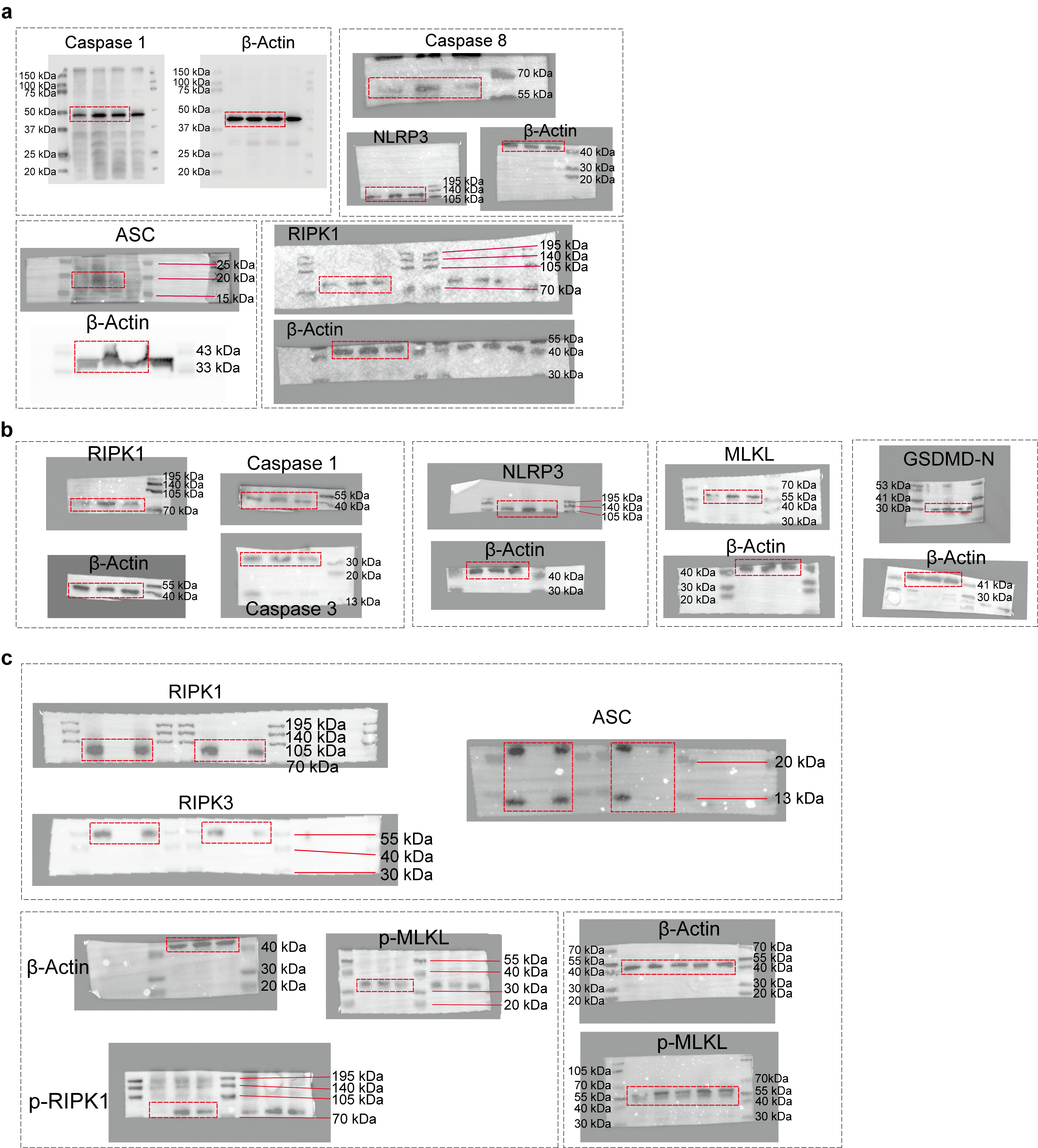


**Fig. S53.** Uncropped western blots for figure 4j (a), figure S33h (b) and figure S34 (c).
